# Heavy metal–induced stress in eukaryotic algae—mechanisms of heavy metal toxicity and tolerance with particular emphasis on oxidative stress in exposed cells and the role of antioxidant response

**DOI:** 10.1007/s11356-021-18419-w

**Published:** 2022-01-10

**Authors:** Beatrycze Nowicka

**Affiliations:** Department of Plant Physiology and Biochemistry, Faculty of Biochemistry, Biophysics and Biotechnology, Jagiellonian University, Gronostajowa 7, 30-387 Kraków, Poland

**Keywords:** Antioxidant enzymes, Eukaryotic algae, Heavy metals, Low-molecular-weight antioxidants, Toxicity mechanisms, Oxidative stress

## Abstract

*Heavy metals* is a collective term describing metals and metalloids with a density higher than 5 g/cm^3^. Some of them are essential micronutrients; others do not play a positive role in living organisms. Increased anthropogenic emissions of heavy metal ions pose a serious threat to water and land ecosystems. The mechanism of heavy metal toxicity predominantly depends on (1) their high affinity to thiol groups, (2) spatial similarity to biochemical functional groups, (3) competition with essential metal cations, (4) and induction of oxidative stress. The antioxidant response is therefore crucial for providing tolerance to heavy metal-induced stress. This review aims to summarize the knowledge of heavy metal toxicity, oxidative stress and antioxidant response in eukaryotic algae. Types of ROS, their formation sites in photosynthetic cells, and the damage they cause to the cellular components are described at the beginning. Furthermore, heavy metals are characterized in more detail, including their chemical properties, roles they play in living cells, sources of contamination, biochemical mechanisms of toxicity, and stress symptoms. The following subchapters contain the description of low-molecular-weight antioxidants and ROS-detoxifying enzymes, their properties, cellular localization, and the occurrence in algae belonging to different clades, as well as the summary of the results of the experiments concerning antioxidant response in heavy metal-treated eukaryotic algae. Other mechanisms providing tolerance to metal ions are briefly outlined at the end.

## Introduction

*Heavy metals* is a collective term describing metals and metalloids with a density higher than 5 g/cm^3^. Some of them are essential micronutrients, necessary in low concentrations and toxic when present in greater amounts. The others do not play any known positive role in living organisms (Nagajyoti et al. 2010). Heavy metals occur mainly in rocks and are released into the environment due to both natural processes and human activities. Natural sources of heavy metals are weathering of rocks and volcanic activity (Nagajyoti et al. 2010). Industrial sources of heavy metals include mining and smelting of metal ores, but also fossil fuel combustion and processes including the production of plastic, textiles, paper and electronics, as well as wood preservation. In agriculture, the production and application of fertilizers, pesticides, and herbicides result in the release of heavy metals into the environment. Other important sources of contamination are transport, domestic effluents, urban runoff, and corrosion of waste products (Pinto et al. 2003; Nagajyoti et al. 2010). Due to increased anthropogenic emissions, heavy metals have become significant pollutants posing a severe threat to water and land ecosystems and for human health (Nagajyoti et al. 2010).

**Fig. 1:**
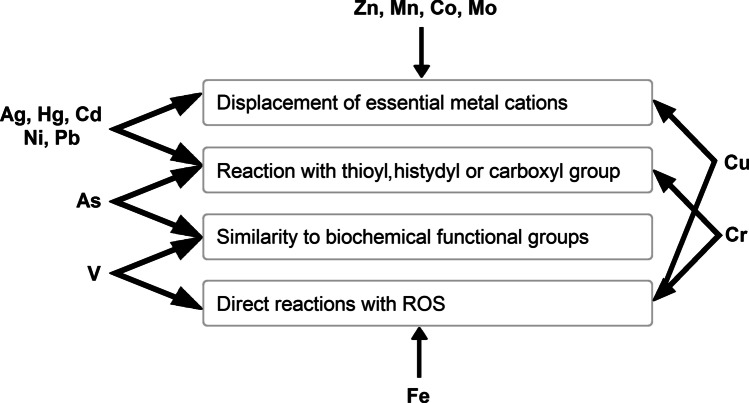
Major mechanisms of toxicity of certain heavy metals

Heavy metals and metalloids are accessible to living organisms in the form of water-soluble ions, which are taken into the cells by active transport and by endocytosis of metal-chelating proteins (Arunakumara and Zhang 2008). Essential and nonessential heavy metals may effectively compete for the same transmembrane carriers (Raskin et al. 1994). Heavy metal toxicity is a complex phenomenon due to its pleiotropic effects, leading to disturbance of various metabolic processes and ultrastructural changes in exposed cells (Nagajyoti et al. 2010). There are four main modes of toxic action of heavy metal ions: (1) reaction with thioyl, histidyl and carboxyl groups of proteins and low-molecular compounds such as glutathione (GSH), which may result in loss of activity, disturbed structure, and changes in regulation and signalling pathways, (2) displacement of essential metal cations, especially those present in active sites of various enzymes, which leads to the loss of activity of these proteins, (3) similarity to biochemical functional groups, mainly phosphate, (4) generation of reactive oxygen species (ROS) by autooxidation and Haber–Weiss cycling (Fig. 1) (Sharma and Dietz 2009; DalCorso 2012).

Excessive amounts of ROS disturb redox homeostasis and damage cell components. The situation when there is an overproduction of ROS is called oxidative stress. Redox-active heavy metals occur in cells in multiple oxidation states and directly react with ROS, leading to the conversion of less harmful ROS into more dangerous ones (Pinto et al. 2003). The induction of oxidative stress is considered the main mode of their toxicity (Stoiber et al. 2013). Nonredox-active metals (redox-inactive metals) usually occur in cells in one oxidative state and do not undergo redox cycling. However, these metals can induce oxidative stress indirectly, by disturbing metabolic processes such as respiration and photosynthesis, causing depletion of GSH or inhibition of antioxidant enzymes (Pinto et al. 2003; Stoiber et al. 2013). The antioxidant response is therefore essential to provide tolerance to the enhanced concentrations of heavy metal ions in the environment (Pinto et al. 2003).

Algae, especially those belonging to marine phytoplankton, are a group of organisms responsible for a large share of biomass production on the Earth (Pinto et al. 2003). Many water ecosystems are endangered by heavy metal contamination. Whereas land plants absorb heavy metals mainly by roots and are often able to limit the transfer of toxic ions to the shoots, in the case of algae, the whole surface of their organisms is exposed to heavy metal ions. The binding of heavy metals by cells causes biomagnification of these pollutants along the aquatic food chain. Algae are also used in biological systems of wastewater treatment (Danouche et al. 2021; Goswami et al. 2021). Therefore, research on the response of algae to heavy metal ions is important. One has to remember that *algae* is an ecological term including species belonging to distinct clades and varying in their chloroplast structure, cell wall composition, and phylogeny of their proteins (Keeling 2004). This variety also applies to antioxidant mechanisms, such as the presence and localization of certain antioxidant enzymes or the amounts of certain low-molecular-weight antioxidants (Asada et al. 1977; Brown and Miller 1992).

## Heavy metals and their toxicity

Considering the density criterion, 53 of the 90 naturally occurring elements are heavy metals. However, the majority of them are not available to living organisms either due to their presence in extremely low amounts or due to the insolubility of their compounds in water (Nies 1999; Schützendübel and Polle 2002). The remaining 17 elements are available to living cells in physiological conditions. These are Ag, As, Cd, Co, Cr, Cu, Fe, Hg, Mn, Mo, Ni, Pb, Sb, U, V, W, and Zn. Among them, Fe, Mn, and Mo are important micronutrients with low toxicity; Co, Cr, Cu, Ni, V, W, and Zn are trace elements displaying higher toxicity, while Ag, As, Cd, Hg, Pb, Sb, and U do not play physiological roles in photosynthetic eukaryotes (Nies 1999). Cd was observed to play a role of a cofactor in carbonic anhydrase in the diatom *Thalassiosira weissflogii* under Zn-limiting conditions, but it seems to be a rare case (Lane and Morel 2000). Heavy metals essential for plants are as follows: Co, Cu, Fe, Mn, Mo, Ni, and Zn (Nagajyoti et al. 2010). Heavy metals of the highest toxicity are as follows: Ag, Cd, Cr, Cu, and Hg (Ratte 1999).

Heavy metals were divided into redox-active and redox-inactive ones depending on the values of the redox potential of their ions. The physiological redox range of aerobic cells usually ranges from − 420 to + 800 mV. If the redox potential of certain heavy metal ion fits in this range, this ion can participate in redox reactions in the cell and therefore is redox-active (Schützendübel and Polle 2002). Chemical properties are a consequence of the atomic structure of an element. Elements with filled orbital *d*, such as As, Cd, Hg, Pb, Sb, and Zn, belong to the redox-inactive ones. Among the rest, the most important redox-active ones are Cu, Cr, and Fe (Nies 1999; Schützendübel and Polle 2002).

### Silver

Silver (Ag) in ionic form Ag^+^ is one of the most toxic heavy metals (Ratte 1999). In the past, it was extensively used in photography; nowadays, this metal is used in electronics (Purcell and Peters 1998). Recently, the contamination of the environment with Ag is due to the common use of silver nanoparticles in food production, cosmetics, antimicrobial agents, clothing, water filters, detergents, and many other goods. Nanoparticles display broad-spectrum antimicrobial properties; they are also harmful to other living organisms. Ag-containing nanoparticles are significantly less toxic than Ag^+^, but they are known to release Ag^+^ to the environment. They also display some toxicity unrelated to ion release and resulting from their ability to disturb cell membranes (Marambio-Jones and Hoek 2010). Hopefully, dissolved Ag^+^ ions are prone to complexation or precipitation in the form of insoluble salts. Algae are able to bioconcentrate Ag^+^ mostly via binding to the cell surface. Well-known toxic action of Ag^+^ results from efficient inhibition of enzyme activity due to binding to the thiol groups (Ratte 1999). In such a way, Ag^+^ inhibits the respiratory electron transport chain. The binding of Ag^+^ to transport proteins leads to proton leakage and collapse of the proton motive force (Marambio-Jones and Hoek 2010). Ag^+^ is capable of competitive substitution of Cu^+^ in plastocyanin, which results in the disturbance or inactivation of the photosynthetic electron transport chain (Yan and Chen 2019). In bacteria, Ag^+^ was also shown to inhibit phosphate uptake. What is more, Ag^+^ may inhibit DNA synthesis and increases the frequency of DNA mutations (Marambio-Jones and Hoek 2010; Moreno-Garrido et al. 2015). Exposure to Ag^+^ leads to a decrease in chlorophyll (Chl) content. An important mode of Ag^+^ toxicity is causing oxidative stress leading to lipid peroxidation, DNA damage, and alteration of cell structure (Yan and Chen 2019).

### Arsenic

A metalloid arsenic (As) is an element relatively abundant in the environment. Over 200 As-containing minerals have been found in nature. Natural processes are the major source of this pollutant; however, human activity also adds to the pool (Farooq et al. 2016). Arsenic is released into the environment as a result of smelting, mining, and use of arsenicals as herbicides, pesticides, feed additives, and wood preservatives (Farooq et al. 2016; Geng et al. 2017). This element can occur in four valency states − 3, 0, + 3, and + 5. Elemental As is very rare and As (− 3) is present only at low pH and in reducing environments. The dominant forms of inorganic As are arsenate (As + 5) and arsenite (As + 3), the latter being reported to be 60 times more toxic than the former. As may also occur in organic compounds (i.e., methylarsonic acid), which are far less toxic than inorganic ones (Neff 1997). Some bacteria are able to use As compounds as electron acceptors in anaerobic respiration, whereas others may use them as electron donors (Nies 1999; Verbruggen et al. 2009). Due to its similarity to phosphate, arsenate is taken into the cells via phosphate transporters. Arsenite is known to enter the cells via aquaglyceroporins and hexose permeases (Wang et al. 2015). The main mechanism of As (+ 5) toxicity is related to the substitution for phosphate in phosphorylation reactions, whereas As (+ 3) toxicity is probably primarily due to high sulphydryl reactivity. Both As (+ 3) and (+ 5) are mutagenic (Verbruggen et al. 2009). Enhanced ROS formation was also observed during As-exposure (Wang et al. 2015). Algae are able to accumulate arsenic compounds (Neff 1997). As ions may be bound to the cell surface or complexed with phytochelatins inside the cells. Arsenite may be oxidized to less toxic arsenate. On the other hand, arsenate can be reduced to arsenite and then exported from the cell, methylated or complexed and sequestrated in vacuoles. Methylated As may undergo further bioconversion to arsenosugars or arsenolipids (Wang et al. 2015).

### Cadmium

Cadmium (Cd) is a nonessential element, highly toxic for all living organisms (Ackova 2018). It is more mobile than many other heavy metals due to the relatively good solubility of its salts in water (Kalaivanan and Ganeshamurthy 2016). In fresh waters, Cd binds to sediments less strongly than Pb, Hg, or Cu (Prasad 1995). The main natural sources of Cd are volcanoes and weathering of rocks (Tran and Popova 2013). Anthropogenic activities release to the environment 3–10 times more Cd than natural processes (Sarkar et al. 2013). This metal is a by-product of Zn and Pb mining and smelting. Cd is widely used in electroplating, as well as in paints, plastic stabilizers and batteries (Prasad 1995; Stohs and Bagchi 1995). It often occurs as contamination in phosphate fertilizers (Tran and Popova 2013). Important sources of Cd input to the marine environment include industrial discharges, domestic waste and atmospheric deposition (Benavides et al. 2005).

Cd toxicity is thought to result from its reactivity towards thiol groups and His residues, interaction with Ca and Zn metabolism, as well as the ability of Cd to cause membrane damage (Nies 1999; Küpper and Andresen 2016). Cd exposure leads to lipid peroxidation. In the experiments on rats, the application of CdCl_2_ caused an increase in the measured Fe content. It was hypothesized that Cd^2+^ may displace Fe ions from their binding sites, which results in Fe-mediated lipid peroxidation (Stohs and Bagchi 1995). The replacement of Zn^2+^ in Cu/Zn superoxide dismutase (SOD) leads to the loss of function of this important antioxidant enzyme (Küpper and Andresen 2016). Cd was also shown to cause GSH depletion in several plants (Benavides et al. 2005).

Cd damages photosynthetic apparatus targeting light-harvesting complexes and both photosystems (DalCorso 2012). Cd disturbs PS II on its acceptor and donor sides, by interaction with Mn cluster, non-heme Fe, and Q_B_ binding pocket (Parmar et al. 2013). This metal inhibits Chl biosynthesis and enzymes involved in CO_2_ fixation (Nagajyoti et al. 2010). Inhibition of Rubisco is caused by the replacement of Mg^2+^ in the catalytic centre of this enzyme. Cd^2+^ may also replace Mg^2+^ in Chl. Cd-Chl quickly dissipates almost all absorbed excitation energy as heat and does not interact properly with Chl-containing protein complexes due to lower Cd affinity for axial ligands (Küpper and Andresen 2016). Alteration of chloroplast structure was also observed in Cd-exposed plants (Tran and Popova 2013).

Cd was reported to disturb respiration in plants and algae (He et al. 2017). This element is known to inhibit many enzymes, such as nitrate reductase, nitrite reductase, glutamine synthetase, glutamate synthetase, carbonic anhydrase, or root Fe^3+^ reductase, an enzyme important for root Fe uptake (DalCorso 2012; Parmar et al. 2013; Ackova 2018). Cd-exposure leads to the decrease in the activity of enzymes important for sulphate assimilation: ATP-sulphurylase and O-acetylserine sulphurylase. The replacement of Zn^2+^ in zinc finger transcription factors with Cd^2+^ results in changed gene expression. What is more, Cd causes DNA strand breaks, DNA–protein crosslinks, chromosomal aberrations, and inhibition of mitosis (DalCorso 2012; Nazar et al. 2012).

In plants, Cd^2+^ is taken mostly by the Ca^2+^ and Zn^2+^ uptake systems and by proteins involved in the transport of other divalent cations (Küpper and Andresen 2016; Ismael et al. 2019). In these organisms, Cd interferes with the uptake, transport, and use of various nutrients (including K, Ca, Fe, Mg, Mn, Zn, P, and S) and disturbs water balance (Nazar et al. 2012; Küpper and Andresen 2016; Ackova 2018). It causes the stomata to close independently of water status, most probably due to interference with Ca^2+^ (DalCorso 2012). Higher plants are known to protect themselves from Cd by binding Cd^2+^ ions extracellularly in roots and intracellularly by phytochelatins, metallothioneins, GSH, and organic acids. The sequestrated Cd is stored in vacuoles (Benavides et al. 2005; Tran and Popova 2013; Ismael et al. 2019). Visible symptoms of Cd toxicity are chloroses, leaf rolling, browning of root tips, growth inhibition, and finally death (Nagajyoti et al. 2010).

### Cobalt

Cobalt (Co) naturally occurs in the Earth’s crust in minerals, where it is mainly in the + 2 oxidation state (Nies 1999). The most important anthropogenic sources of Co are smelting activities, industrial waste, and the use of fertilizers (Palit et al. 1994; Li et al. 2009). The physiological role of this element is related to its occurrence in cofactor B_12_. Enzymes containing this metal have been also discovered (Nies 1999). Algae are able to accumulate Co^2+^ and large uptake of this element may limit the growth of these organisms (Palit et al. 1994). The knowledge concerning the phytotoxic action of Co^2+^ is scarce. In higher plants, the excess of Co resulted in growth inhibition, decrease in Fe content, disturbed transport of other nutrients, such as P, S, Mn, Zn, and Cu, and decrease in Chl content and catalase (CAT) activity (Nagajyoti et al. 2010). Co applied in high concentrations was shown to inhibit RNA synthesis and activity of PS II, nitrate reductase, and phosphoenol pyruvate carboxylase crucial for CO_2_ assimilation in C_4_ and CAM plants. It was also shown to disturb the mitotic spindle (Palit et al. 1994). The toxic action of Co^2+^ was postulated to result from competitive interactions with other metal ions (Liu et al. 2000).

### Copper

Copper (Cu) is widely distributed in nature and is an essential element (Stohs and Bagchi 1995). However, in higher concentrations, it is toxic, especially for photosynthetic organisms, which display metabolic disturbances when Cu intracellular content is only slightly higher than the optimal level. Cu is one of the most toxic heavy metals to aquatic plants and algae, due to the fact that it is more mobile in water than in the soil, where most Cu ions are bound to soil components. Microalgae are probably the organisms most sensitive to Cu toxicity (Fernandes and Henriques 1991). In the open oceans, organisms rather suffer from the deficiency of nutrients, but in the Sargasso Sea Cu is naturally abundant enough to reach toxic levels. In freshwater ecosystems, the increased Cu content is mostly anthropogenic (Küpper and Andresen 2016). Enhanced mining, smelting, and other industrial activities result in contamination with Cu (Nagajyoti et al. 2010). The application of Cu-containing pesticides and fungicides is a source of contamination of arable land (Yruela 2009; Küpper and Andresen 2016).

Cu occurs in 0, + 1, and + 2 oxidation states (Flemming and Trevors 1989). The unique electron structure of this element permits the direct interaction of this metal with spin-restricted ^3^O_2_ (Harris and Gitlin 1996). The electrochemical potential of Cu^2+^/Cu^+^ is − 268 mV, which is within the physiological range and facilitates the interconversion of these ions (Nies 1999). Due to its redox properties, Cu is a prosthetic group in many enzymes catalysing redox reactions, such as cytochrome oxidase (mitochondrial complex IV) or Cu/ZnSOD, as well as in proteins functioning as electron carriers, such as plastocyanin or auracyanins, the latter present in green filamentous bacteria (Nagajyoti et al. 2010; Nowicka and Kruk 2016). In higher plants, chloroplasts contain 35–90% of total foliar Cu and about half of chloroplast Cu is present in plastocyanin (Fernandes and Henriques 1991). Cu is crucial for the functioning of photosynthesis, respiration, and many other metabolic processes. This element is also a structural component in some regulatory proteins (DalCorso 2012). However, the above-mentioned properties make Cu easily undergo unwanted and uncontrolled redox cycling in living cells. Well-known reactions are as follows:

$${\mathrm{Cu}}^{2+} + {{\mathrm{O}}_{2}}^{\cdot -} \to {\mathrm{Cu}}^{+} + {\mathrm{O}}_{2}$$

$${\mathrm{Cu}}^{+} + {\mathrm{H}}_{2}{\mathrm{O}}_{2} \to {\mathrm{Cu}}^{2+} + {\mathrm{OH}}^{-} + {\mathrm{OH}}^{\cdot }$$

Cu^2+^ may also be reduced by Asc. Therefore, Cu ions are able to directly catalyse the formation of the most dangerous ROS, ${\mathrm{OH}}^{\cdot }$. The capacity to produce ROS is thought to be the main mechanism of Cu toxicity (Rowley and Halliwell 1983; DalCorso 2012).

Under lower, but still excessive concentrations, a prime target of Cu toxicity is the light phase of photosynthesis (Küpper and Andresen 2016). Cu inhibits O_2_ evolution in PS II by interaction with Tyr_Z_ and Tyr_D_ in PS II core peptides. When applied in very high concentrations, it enhances degradation of extrinsic proteins of Oxygen Evolving Complex (Yruela 2005; DalCorso 2012). Cu may also disturb PS II activity via interacting with non-heme Fe, cyt *b*_559_ and at sites close to pheophytin, Q_A_ and Q_B_ binding pockets (Burda et al. 2003; Yruela 2005). This element is known to hamper the function of LHC II antennae due to the substitution of Mg^2+^ in Chl that leads to thermal dissipation of the captured excitons (Küpper and Andresen 2016). Cu inhibits enzymes crucial for CO_2_ assimilation in the dark phase of photosynthesis, such as Rubisco and phosphoenolpyruvate carboxylase of C_4_ plants. Exposure to toxic concentrations of Cu ions causes the damage to the chloroplast structure (DalCorso 2012). This effect was postulated to result from both lipid peroxidation and the disturbance of biosynthesis of photosynthetic machinery (Yruela 2005).

Cu-toxicity effects observed in higher plants are stunted growth, reduction in Chl content, disruption of nitrogen metabolism, and disturbance of nutrient uptake (DalCorso 2012; Küpper and Andresen 2016). In particular, the ability of Cu to induce Fe-deficiency was postulated. Cu is a strong activator of phytochelatin synthesis, but phytochelatin-deficient mutants showed relatively little Cu sensitivity (Yruela 2009). In brown algae exposed to toxic concentrations of Cu^2+^ these ions were sequestered inside the cell, chelated with phenolic compounds (Smith et al. 1986). The most important defence mechanisms in green and red marine macroalgae are as follows: binding of Cu ions to cell walls and epibionts, synthesis of phytochelatins and metallothioneins, as well as the enhancement of the antioxidant response (Moenne et al. 2016). Diatoms were reported to bind Cu in polyphosphate bodies in vacuoles. Some green algae and diatoms respond to Cu by releasing Cu-complexing compounds into the water (Fernandes and Henriques 1991).

### Chromium

Chromium (Cr) may occur in several oxidation states; however, the most stable and common are Cr (+ 3) and Cr (+ 6). The latter is considered the most toxic form of Cr and usually occurs as oxyanions, chromate (CrO_4_^2−^) and dichromate (Cr_2_O_7_^2−^). On the other hand, Cr (+ 3) most often occurs as a trivalent cation in oxides, hydroxides, and sulphates, and is much less mobile (Nies 1999; Cervantes et al. 2001). Cr is the 7^th^ most abundant element on the Earth. It is very widely used, mostly in alloys, but also in chemical industrial processes, such as electroplating, pigment production, leather tanning and wood treatment (Stohs and Bagchi 1995; Cervantes et al. 2001). As a result of these applications, Cr has become a serious environmental pollutant.

Cr is a highly toxic nonessential metal for microorganisms and plants (Cervantes et al. 2001). Chromate is taken into the cells via the sulphate uptake system (Nies 1999). Nonspecific anion carriers also play a role in Cr (+ 6) import (Stohs and Bagchi 1995). In the case of Cr^3+^, independent uptake mechanisms were observed in plants. Algae are able to accumulate Cr. It was shown that green algae retain more of this metal than brown or red algae (Cervantes et al. 2001). Cr toxicity is related to the redox reactions of its ions inside the cells. Reduction of Cr (+ 6) to lower oxidation states, reported in many biological systems, results in the formation of free radicals. Among cellular compounds and processes able to reduce Cr (+ 6), there are such crucial and abundant ones as NAD(P)H, FADH_2_, GSH, Asc, cytochrome P-450, several pentoses, and the respiratory electron transport chain (Cervantes et al. 2001). Cr (+ 3) may be reduced by NADH and Cys. Cr (both + 6 and + 3) may be also reduced by O_2_^•−^. The examples of Cr redox reactions are as follows:

$$\mathrm{Cr }(+6) + {\mathrm{e}}^{-} \to \mathrm{ Cr }(+5)$$

$$\mathrm{Cr }(+5) + {\mathrm{H}}_{2}{\mathrm{O}}_{2} \to \mathrm{ Cr }(+6) + {\mathrm{OH}}^{-} + {\mathrm{OH}}^{\cdot }$$

$$\mathrm{Cr }(+3) + {\mathrm{e}}^{-}\to \mathrm{ Cr }(+2)$$

$$\mathrm{Cr }(+2) + {\mathrm{H}}_{2}{\mathrm{O}}_{2} \to \mathrm{ Cr }(+3) + {\mathrm{OH}}^{-} + {\mathrm{OH}}^{\cdot }$$

Therefore, Cr exposure results in the formation of extremely dangerous ${\mathrm{OH}}^{\cdot }$. Reduced Cr forms may also react with LOOH, which leads to the generation of ${\mathrm{LO}}^{\cdot }$, a radical able to induce lipid peroxidation (Stohs and Bagchi 1995). Oxidative damage of DNA is considered a mechanism responsible for the genotoxic action of Cr. Cr (+ 3) may react with the carboxyl and thiol groups of enzymes disturbing their structure and function (Cervantes et al. 2001). Cr (+ 6) is able to inhibit mitochondrial complexes I and IV, and damage the oxygen-evolving complex in PS II (Singh et al. 2013). Cr-induced stress leads to the decrease in photosynthetic and respiration rates, disturbance of chloroplasts’ ultrastructure, and cytoskeleton alterations (Cervantes et al. 2001; Nagajyoti et al. 2010). In higher plants, Cr was shown to disturb the uptake of various macro- and micronutrients. This effect can be partially attributed to the inhibition of certain cation-ATPases by Cr (+ 6) and Cr (+ 3) (Shanker et al. 2005; Singh et al. 2013). In microorganisms, Cr-resistance mechanisms include biosorption, diminished accumulation, reduction of Cr (+ 6) to Cr (+ 3), precipitation, and efflux (Cervantes et al. 2001).

### Iron

Iron (Fe) is the only macronutrient of heavy metals (Nies 1999). The most common oxidation states of Fe are + 2 and + 3. In aerobic conditions, Fe^2+^ ions are prone to oxidation to Fe^3+^ (Küpper and Andresen 2016). Fe^3+^ forms iron hydroxides and salts of very low solubility, therefore it is not easily available to living organisms (Nies 1999; Küpper and Andresen 2016). Fe^2+^ serves as an electron donor for some chemosynthetic bacteria, while Fe^3+^ may play the role of electron acceptor in microbial anaerobic respiration (Nies 1999; Schoepp-Cothenet et al. 2013). In higher plants, Fe toxicity symptoms occur only under flooded conditions, when anaerobic bacteria cause an increase in the content of Fe^2+^ in the soil (Nagajyoti et al. 2010). This makes Fe-toxicity an important stress factor limiting rice production in some areas (Fageria et al. 2008). In the oceans, Fe is always deficient (Küpper and Andresen 2016).

The redox properties make Fe a crucial constituent of several enzymes and electron-carrier proteins, for example, this element is present in haem and Fe-S clusters (Nagajyoti et al. 2010). Similar to Cu, the redox properties of the Fe^3+^/Fe^2+^ couple make Fe both useful and dangerous for living organisms. Free Fe ions undergo redox cycling in cells, resulting in the formation of ${\mathrm{OH}}^{\cdot }$ and ${\mathrm{RO}}^{\cdot }$, the latter may cause re-initiation of lipid peroxidation in membranes.

$${\mathrm{Fe}}^{3+} + {{\mathrm{O}}_{2}}^{\cdot -} \to {\mathrm{Fe}}^{2+} + {\mathrm{O}}_{2}$$

$${\mathrm{Fe}}^{2+} + {\mathrm{H}}_{2}{\mathrm{O}}_{2} \to {\mathrm{Fe}}^{3+} + {\mathrm{OH}}^{-} + {\mathrm{OH}}^{\cdot }$$

$${\mathrm{Fe}}^{2+} +\mathrm{ ROOH }\to {\mathrm{Fe}}^{3+} + {\mathrm{OH}}^{-} + {\mathrm{RO}}^{\cdot }$$

Thus, the major cause of Fe toxicity is its prooxidant action (Stohs and Bagchi 1995; Vranová et al. 2002; Niki 2009; DalCorso 2012). Excessive Fe reduces photosynthetic activity and water transpiration in land plants (DalCorso 2012). The characteristic visual symptom of Fe toxicity in rice is bronzing of leaves resulting from the accumulation of oxidized polyphenols (DalCorso 2012). In some species of higher plants, Fe toxicity is associated with Zn deficiency (Kalaivanan and Ganeshamurthy 2016).

### Mercury

Mercury (Hg) is considered to be the most toxic heavy metal for microorganisms (Ratte 1999). Among heavy metals, Hg is unique due to its existence in different forms: Hg^2+^, Hg^+^, Hg^0^, and organomercurials like methyl-, ethyl-, and phenyl-Hg (Patra and Sharma 2000; Nagajyoti et al. 2010). Organomercurials are the most toxic form of Hg (Mahbub et al. 2017). Hg^2+^, which is a form common in the environment, is soluble, highly reactive, and can be accumulated in higher plants and aquatic organisms (Nagajyoti et al. 2010; DalCorso 2012). Hopefully, in the soil, it occurs mostly bound to minerals and soil organic matter (Mahbub et al. 2017). In water ecosystems, Hg toxicity is affected by temperature, salinity, dissolved O_2_, and water hardness (Boening 2000). Interconversions of various Hg forms occur in water and soil due to the activity of prokaryotes. One of the processes performed by these microorganisms is the biomethylation of this metal (Wood and Wang 1983). Another one is the reduction of Hg^2+^ to Hg^0^. Such a reaction was also observed to occur in many phytoplankton species (Küpper and Andresen 2016). Mercury has the potential for biomagnification in food chains (Shrivastava et al. 2015). About one-third of Hg emissions into the environment results from human activity (Kalaivanan and Ganeshamurthy 2016). The predominant sources of Hg contamination are mining, smelting, coal burning, and industrial waste (Chen and Yang 2012; DalCorso 2012). This metal is also released into the environment with sludge and fungicides (Kalaivanan and Ganeshamurthy 2016).

Hg does not play any known physiological role (Küpper and Andresen 2016). The main cause of the high toxicity of Hg^2+^ is its high affinity to thiol groups and its similarity to Zn (Stohs and Bagchi 1995; Küpper and Andresen 2016). This element was also postulated to be harmful due to the affinity to phosphate groups including those in ATP (Patra and Sharma 2000). It can also react with carboxyl, amide, and amine groups (Azevedo and Rodriguez 2012). Hg ions are readily taken by plant roots, but the majority of them remain in these organs bound to the cell walls (Chen and Yang 2012). Hg concentration in shoots appears to depend largely on the uptake of volatile Hg^0^ by leaves (Patra and Sharma 2000). In higher plants, Hg^2+^ is known to bind to aquaporins that, among other effects, induce stomata closure. Hg disturbs mitochondrial activity and induces oxidative stress (Nagajyoti et al. 2010). The inhibition of light and dark phases of photosynthesis by Hg and the ability of this metal to replace Mg in Chl was also reported (Kalaivanan and Ganeshamurthy 2016). The inhibition of PS II by Hg was postulated to occur at its donor side (Patra and Sharma 2000). Hg exposure also leads to chromosomal damage and disturbance of mitosis (DalCorso 2012). Similar to other heavy metals, in higher plants, Hg causes growth reduction, decrease in Chl content, and disturbance of nutrient balance (Shrivastava et al. 2015). Exposure to Hg induces the synthesis of protective thiol compounds (GSH, phytochelatins) and Pro in plants (Küpper and Andresen 2016). Hg efflux system present in bacteria has been characterized (Patra and Sharma 2000). The binding of Hg ions to phytochelatins and converting Hg^2+^ into dissolved gaseous Hg^0^ and metacinnabar was observed in phytoplankton species, green alga *Chlorella autotrophica*, dinoflagellate *Isochrysis galbana*, and diatom *Thalassiosira weissflogii* (Wu and Wang 2014).

### Manganese

Manganese (Mn) exists in various oxidation states, from + 2 to + 7, with the Mn^2+^ cation being the predominant form (Nies 1999). Mn is a common metal in the Earth’s crust and is released into the environment mainly due to natural processes. However, human activities, such as mining, smelting, and some agricultural practices result in an increase in Mn content in certain soils (Paschke et al. 2005). The occurrence of Mn in a particular oxidation state depends on soil pH and redox conditions (Li et al. 2019). More soluble and due to it more bioavailable Mn (+ 2) becomes more abundant below pH 5.5, while less soluble Mn (+ 3) and Mn (+ 4) become more abundant above pH 6.5 (DalCorso 2012). Mn is absolutely crucial for oxygenic photosynthesis because Mn cluster is a site of H_2_O oxidation in PS II. This element is also a cofactor of enzymes such as MnSOD, Mn-catalase, phosphoenol pyruvate carboxykinase, pyruvate carboxylase, malic enzyme, isocitrate lyase, RNA polymerases and many others (Millaleo et al. 2010; DalCorso 2012; Li et al. 2019). Mn also plays the role of enzyme activator (Li et al. 2019). Mn ions are used by some bacteria as electron acceptors in anaerobic respiration (Nies 1999). The toxicity of this element is relatively low (Nies 1999). When applied in excess to higher plants, Mn causes chloroses, necroses, browning of tissues, and inhibition of Chl synthesis. Another common symptom, called “crinkle-leaf”, occurs in young leaves (Nagajyoti et al. 2010). Excessive Mn may interfere with the absorption and utilization of other nutrients, for example, it is known to induce Fe, Ca, and Mg deficiency (El-Jaoual and Cox 1998; Paschke et al. 2005; Kalaivanan and Ganeshamurthy 2016). The occurrence of oxidative stress and lipid peroxidation was also observed in Mn-exposed plants (DalCorso 2012). In plants, tolerance to Mn has been attributed to restricted absorption and transport, and greater tolerance to high Mn levels within plant tissues (El-Jaoual and Cox 1998). The latter is thought to result from sequestration by organic compounds in metabolically less-active cells or organelles (Millaleo et al. 2010).

### Molybdenum

Molybdenum (Mo) occurs mostly as molybdate, oxyanion containing Mo on the + 6 oxidative state, but this element can also exist on + 4 oxidation state (Nies 1999; Evans and Barabash 2010). Mo is an important micronutrient present in enzyme cofactors (Nies 1999). Mo-containing enzymes participate in nitrogen metabolism (e.g., nitrogenase, nitrite reductase), sulphur metabolism, purine catabolism, and hormone biosynthesis (McGrath et al. 2010). Mo is used in the metallurgy and chemical industry and the contamination with this metal is mostly observed in soils around urban complexes and industrial sites (Evans and Barabash 2010). The toxicity of Mo is considered to be low and it has not been extensively investigated. Higher plants exposed to excessive Mo display chlorosis (Singh et al. 2010). Application of toxic concentration of Mo-containing salts to *Euglena gracilis* resulted in the abnormal cell division (Colmano 1973).

### Nickel

Nickel (Ni) is abundant in rocks as a free metal and as a complex with other metal ions such as Fe (DalCorso 2012). Ni has several oxidation states ranging from –1 to + 4, but in soil, water, and biological systems, it occurs mostly in Ni^2+^ cationic form (Nies 1999; Shahzad et al. 2018). Anthropogenic activities including mining, smelting, burning fossil fuels, electroplating, cement industry, transport, and disposal of batteries result in contamination with Ni (DalCorso 2012; Shahzad et al. 2018). Ni is a micronutrient needed for the proper function of some enzymes. Well-known examples of such enzymes are urease and glyoxalase I occurring in plants and microorganisms (Shahzad et al. 2018). Other Ni-containing enzymes are present in microorganisms: NiFe hydrogenases, acetyl-S-CoA synthase in anaerobic prokaryotes, CO dehydrogenase, Ni-dependent SOD, peptide deformylase, acireductone dioxygenase, and methyl-coenzyme-M reductase with its Ni-tetrapyrrole cofactor F_430_ occurring in methanogenic archaebacteria (Macomber and Hausinger 2011; DalCorso 2012; Shahzad et al. 2018).

In higher plants, Ni toxicity results in growth retardation, chloroses, necroses, and impairment of water balance, nutrient uptake and translocation (Seregin and Kozhevnikova 2006; Nagajyoti et al. 2010). Other symptoms observed in plants exposed to excessive Ni were chromosome aberrations and disturbed structure of the chloroplast and nucleus (Seregin and Kozhevnikova 2006). The competition of Ni^2+^ with other metal cations was postulated to be important for the toxic action of this heavy metal (Shahzad et al. 2018). The replacement of Mg^2+^ in Chl by Ni^2+^ was observed. The excited state of Ni-Chl is very unstable what leads to the thermal dissipation of all absorbed energy (Küpper and Andresen 2016). Ni can also displace Mg from enzymes such as Rubisco and inhibit PS I and PS II activity (DalCorso 2012). The inhibition of Calvin cycle enzymes other than Rubisco by Ni was also observed (Shahzad et al. 2018). Ni^2+^ may replace other metal divalent ions, for example, Ca^2+^ in oxygen-evolving complex in PS II, or Fe^2+^ in *E. coli* iron- and α-ketoglutarate-dependent dioxygenases (Macomber and Hausinger 2011; Sreekanth et al. 2013). Exposure to this element results in the occurrence of oxidative stress and lipid peroxidation. Free Ni ions are not thought to directly react with ROS in cells (Shahzad et al. 2018). Considering binding to certain chemical groups, Ni^2+^ would rather be bound to aromatic nitrogen than thiols (Seregin and Kozhevnikova 2006). In bacteria and yeast, Ni is detoxified by sequestration and efflux (Nies 1999). In higher plants, chelation by organic acids and sequestration in the vacuole was observed (Shahzad et al. 2018).

### Lead

Lead (Pb) is one of the most abundant heavy metals in terrestrial and aquatic environments. Anthropogenic release of this element has been a significant source of Pb contamination. Pb is released as a result of mining, smelting, metal plating, paper production, disposal of municipal sewage sludge, and use of Pb-containing fuels, explosives, and paints. Pb is one of the most serious hazards to human health (Yadav 2010; DalCorso 2012; Kaur 2014). In soil, this element may occur as Pb^2+^, free or complexed with inorganic and organic compounds, or adsorbed onto particle surfaces. Because of strong binding with organic and colloidal material, only a small amount of Pb in the soil is soluble (Pourrut et al. 2011). In sea water, Pb is not so dangerous due to its low solubility and therefore, low bioavailability (Nies 1999). Pb is a nonessential element. In plant roots, Ca^2+^ ion channels play a role in Pb uptake. Hopefully, only a limited amount of this element is translocated to the shoots (Pourrut et al. 2011).

Primary toxic effect of Pb^2+^ results from an extensive reaction with thiol groups leading to the inhibition of enzyme activity (Ackova 2018). Pb may also interact with carboxyl and amine groups and displace other metals from metalloenzymes (Pourrut et al. 2011). It can replace Mn in PS II. Pb is known to strongly inhibit Chl biosynthetic enzymes and many enzymes of the Calvin cycle, which leads to a decrease in photosynthetic rate (Sharma and Dubey 2005; DalCorso 2012). The inhibition of carotenoid and plastoquinone (PQ) synthesis by Pb was also reported (Pourrut et al. 2011). Another toxic effect of Pb is interfering with the alignment of microtubules on the mitotic spindle (DalCorso 2012). Defect in mitosis in response to Pb-exposure occurs at low concentrations of its salts applied, therefore this effect was postulated to be environmentally the most relevant (Küpper 2017). Pb is also known to induce oxidative stress and, as a result, to cause lipid peroxidation (Yadav 2010; Kaur 2014). In higher plants, Pb- exposure leads to the disturbance of morphology, photosynthesis, mineral nutrition, and water balance (Yadav 2010). Chloroses and growth inhibition are other symptoms observed (DalCorso 2012). Pb-resistance in bacteria is based mainly on efflux (Nies 1999), while higher plants are known to bind Pb^2+^ ions in the cell wall or complex it with phytochelatins, GSH or amino acids, and sequester these complexes in vacuoles and chloroplasts (Sharma and Dubey 2005).

### Vanadium

Vanadium (V) exists in nature in a range of oxidation states from + 2 to + 5. Under environmental conditions, in the solution, the most common forms are vanadyl (V + 4) and oxyanion vanadate (V + 5). The former occurs under moderately reducing conditions, the latter is common under aerobic conditions at pH higher than 4 (Larsson et al. 2013). The toxicity of V compounds usually increases with increasing valence. V is widely distributed in nature. There are about 65 known V-bearing minerals, and rock weathering is the main source of this element (Madejón 2013; Imtiaz et al. 2015). The most important anthropogenic sources of V are associated with the burning of fossil fuels, mining, and use of this element in alloys and as a catalyst in the chemical industry (Madejón 2013; Larsson et al. 2013). There are few examples of the physiological role of V. Some N_2_-fixing bacteria synthesize alternative V-dependent nitrogenase in the situation of Mo deficit (Madejón 2013). Optional replacement of Mo with V in nitrate reductase was observed in bacteria *Pseudomonas isachenkovii* (Rehder 2015). Vanadate is also a prosthetic group in V-dependent haloperoxidases occurring in some bacteria, fungi, as well as green, red, and brown macroalgae (Wever and Kustin 1990). There are also known prokaryotes using vanadate as an electron acceptor in anaerobic respiration (Nies 1999).

Vanadate is structurally similar to phosphate and may be taken by phosphate uptake systems (Nies 1999). Due to this similarity, vanadate is able to inhibit phosphate metabolizing systems (Larsson et al. 2013). It is known to be bound by ATPases what leads to the inhibition of these enzymes (Nies 1999). The V-evoked disturbance of transmembrane transport and kinase-dependent signal transduction was also observed (Imtiaz et al. 2015). Another mechanism of V toxicity is related to redox reactions and ROS generation. Similar to Cr, Cu, or Fe, V may undergo redox cycling. It was shown in in vitro experiments that V (+ 5) may be reduced to V (+ 4) by O_2_^•−^ or flavoenzymes using NADPH as an electron donor. V (+ 4) then reacts with H_2_O_2_ what results in OH• formation (Stohs and Bagchi 1995). The oxidation of V (+ 4) to V (+ 5) by O_2_ leading to the formation of O_2_^•−^, and the participation of V (+ 4) in H_2_O_2_ generation have also been proposed to occur (Imtiaz et al. 2015).

### Zinc

Zinc (Zn) occurs exclusively as the Zn^2+^ (Nies 1999). It is usually abundant in soils, in the mineral components such as oxides, phosphates, carbonates, sulphides, sulphates and silicates (DalCorso 2012). Anthropogenic sources of Zn release to the environment are mining, smelting, burning fossil fuels, limestone topping, and use of phosphate-based fertilizers (DalCorso 2012). The ratio of Zn emissions arising from anthropogenic to natural inputs was estimated to exceed 20:1 (Broadley et al. 2007). The sources of Zn contamination are often associated with the sources of Cd, Cu and Pb (Tsonev and Cebola Lidon 2012). Zn is an essential micronutrient playing a role in many crucial processes, such as enzyme activation and metabolism of proteins, lipids, nucleic acids, and carbohydrates. It is a cofactor of several enzymes and a component of many transcription factors (DalCorso 2012). In the majority of organisms, Zn is the second most abundant transition metal after Fe and the only metal represented in all six enzyme classes (Broadley et al. 2007). The toxicity of Zn is rather low. In many parts of the world, Zn deficiency is more often than toxicity (Küpper and Andresen 2016).

Zn toxicity originates mostly from the replacement of other weakly bound divalent metal cations. This element may replace Mg in Chl. Zn-bacteriochlorophyll occurs naturally in photosynthetic anoxygenic bacteria living in a highly acidic environment due to the stability of this pigment in acidic conditions (Nowicka and Kruk 2016). Zn-Chl is more prone to heat dissipation of its excited states than Chl. What is more, due to the diminished tendency of Zn-Chl to bind axial ligands, protein complexes that evolved to bind Chl would not fold properly and be stable when binding Zn-containing pigments (Küpper and Andresen 2016). The inhibitory action of Zn^2+^ on PS II was postulated to result from the replacement of Mn^2+^ or Ca^2+^ in the Mn cluster, while the reduction of Rubisco carboxylase activity most probably is an effect of Mg^2+^ substitution (Küpper and Andresen 2016). The inhibition of PS II on its donor side was also reported (Tsonev and Cebola Lidon 2012). Zn-induced stress in plants leads to chloroses, increased anthocyanin synthesis, necroses, and inhibition of growth and photosynthesis (DalCorso 2012; Küpper and Andresen 2016). Zn was shown to cause Fe^2+^, Mn^2+^, and Cu^2+^ deficiency, which was proposed to result from the hindered transport of these ions (Yadav 2010). Zn is not a redox-active metal, but it is able to induce oxidative stress (DalCorso 2012).

## Oxidative stress

### Reactive oxygen species

ROS are inevitable by-products of aerobic metabolism (Halliwell 2006). They include both radical and nonradical forms, which easily react with organic molecules leading to damage of cell components (Gechev et al. 2006). Aerobic organisms have evolved various antioxidant mechanisms, but also have learned how to use ROS for their benefit, as signalling molecules and in response to pathogen attacks (Van Breusegem et al. 2008). Excessive ROS formation often occurs under stress conditions, as a result of disturbance of metabolism (Gechev et al. 2006).

Atomic oxygen in its ground state has an unusual electron configuration. It is diradical as it has two unpaired electrons with parallel spins in two antibonding orbitals π*2p. In the external magnetic field, it has three energy levels; therefore, it is called triplet oxygen (^3^O_2_). This configuration makes ^3^O_2_ less reactive because the majority of chemical compounds have paired antiparallel electrons in their molecular orbitals (Halliwell 2006). The excitation of ^3^O_2_ causes spin reversal of one of the unpaired electrons that leads to the formation of singlet oxygen (^1^O_2_). There are two singlet states of O_2_: ^1^*Σ*_g_^+^O_2_ having electrons of opposite spins still in separate orbitals, and ^1^*Δ*_g_O_2_ having paired electrons in one of the π*2p orbitals. The ^1^*Σ*_g_^+^ state is very short-lived and it undergoes conversion to ^1^*Δ*_g_ state of lower energy. The latter has a lifetime long enough (4 μs in water) to react with other molecules. Paired electrons make ^1^O_2_ much more reactive than ^3^O_2_ (Triantaphylidès and Havaux 2009). It reacts with compounds containing unsaturated bonds, such as photosynthetic pigments, leading to the formation of cycloadducts, hydroperoxides and endoperoxides. Membrane lipids usually contain this kind of bonds; therefore, ^1^O_2_ causes lipid peroxidation (Triantaphylidès and Havaux 2009). It also oxidizes sulphides to sulphoxides. Considering proteins, amino acid residues susceptible to oxidation by ^1^O_2_ are Trp, Tyr, His, Met and Cys, while in nucleic acids this ROS predominantly oxidizes guanine. ^1^O_2_ is considered the major ROS responsible for leaf damage and light-induced loss of PS II activity (Triantaphylidès and Havaux 2009; Nowicka and Kruk 2013).

**Fig. 2:**
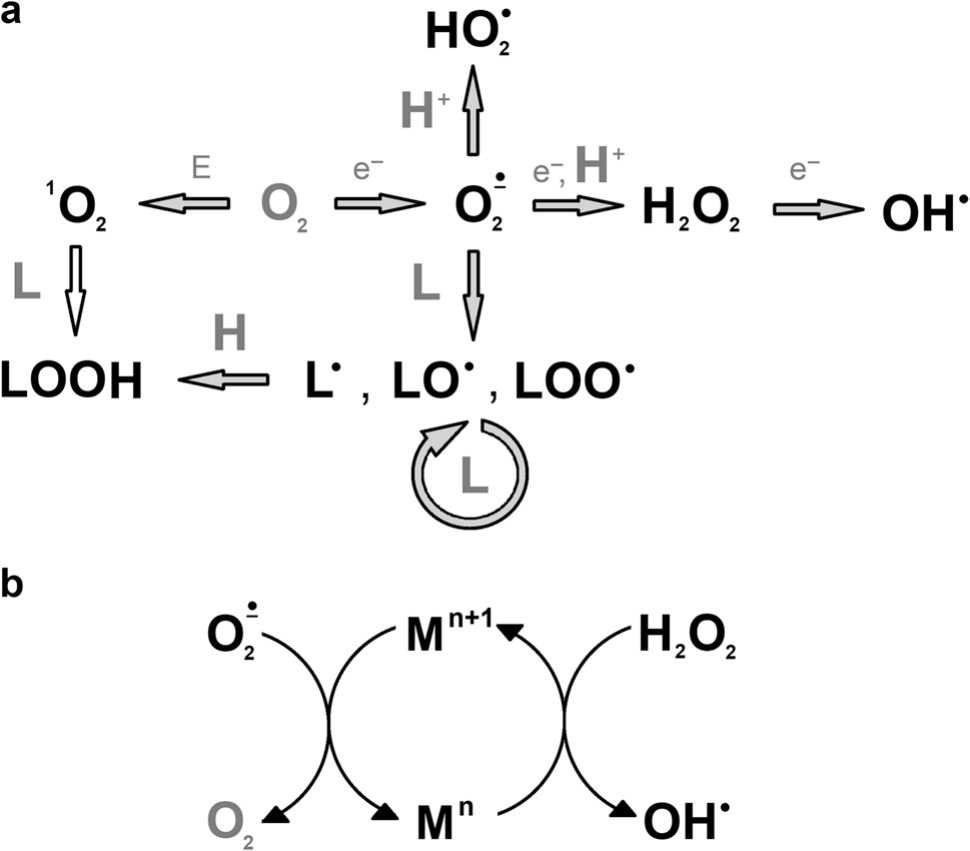
Reactive oxygen species, their inter-conversions, and reactions with lipids (**a**) and with ions of redox-active metals (**b**). ROS and lipid radicals formed during lipid peroxidation are marked in black bold font. Empty arrows indicate both non-enzymatic and enzyme-catalysed reactions. E, excitation energy; L, lipid

Molecular oxygen can also be reduced. Full four-electron reduction results in the formation of one water molecule, while all its intermediates belong to ROS (Fig. 2a). The first, one-electron reduction, requires energy, while the next steps may occur spontaneously (Edreva 2005). The product of one-electron reduction, superoxide anion (O_2_^•−^), is known to damage Fe-S clusters in enzymes. It can also reduce transition metals (e.g., Fe^3+^, Cu^2+^) and react with Cys thiol groups. Other amino acids particularly susceptible to O_2_^•−^ are His, Met and Trp. The reaction of O_2_^•−^ with compounds containing double bonds results in the formation of hydroperoxides. The reaction of O_2_^•−^ with nitric oxide (NO) leads to the formation of highly oxidizing peroxynitrite (ONOO^−^) (Van Breusegem et al. 2001; Nowicka and Kruk 2013). In low pH O_2_^•−^ is protonated to hydroperoxide radical (HO_2_^•^). Being not charged, HO_2_^•^ can diffuse in biological membranes and initiate lipid peroxidation (Gechev et al. 2006).

The product of two-electron reduction of O_2_, hydrogen peroxide (H_2_O_2_), is relatively stable, but due to lesser reactivity, it has a diffusion range greater than that of ^1^O_2_ or O_2_^•−^. Being electrically neutral, H_2_O_2_ can diffuse across membranes (Gechev et al. 2006). H_2_O_2_ reacts with thiol, indole, imidazole, phenol, thioester, and methionyl groups. It also damages the Mn cluster in PS II and haem groups. The reaction of H_2_O_2_ with transition metals (e.g., Fe^2+^, Cu^+^) results in the formation of (Fig. 2b). Such a reaction, where H_2_O_2_ reacts with Fe^2+^, is called the Fenton reaction, whereas the whole cycle of OH• generation in the presence of Fe ions and H_2_O_2_ is known as the Haber–Weiss reaction (García-Caparrós et al. 2020). Analogical reaction, where Cr ions acted as catalysts, was postulated to occur in the chloroplasts of Cr-treated soybean (Balasaraswathi et al. 2017). Free metal ions tend to bind to the surface of proteins and DNA, where they can participate in OH• generation (Edreva 2005; Nowicka and Kruk 2013). OH• is the most reactive ROS, able to react with any molecule in its vicinity at a rate limited only by diffusion. Due to it, the destructive action of OH• is practically limited to the place of its formation (Halliwell 2006).

Other types of ROS are ozone (O_3_) and compounds formed as a result of the reaction of any of the above-mentioned forms with organic molecules, such as alkoxy radicals (RO^•^, where R can be a fatty acid residue), peroxy radicals (ROO^•^), and hydroperoxides (ROOH) (Nowicka and Kruk 2013).

### ROS formation in cells

There are three main types of ROS-producers in photosynthetic organisms: electron-transport chains, some enzymes, such as NADPH oxidase or xanthine oxidase, and photosensitizers, particularly chlorophylls (Edreva 2005). Considering the sites of ROS production, the most important ones are chloroplasts, mitochondria, and peroxisomes (Van Breusegem et al. 2008). In plant green tissues, chloroplasts are the main ROS-formation sites due to the functioning of the photosynthetic electron transport chain and the high concentration of O_2_ in these organelles (Gechev et al. 2006). ROS generation in chloroplasts is enhanced when the dark phase reactions of photosynthesis are slowed down, for example under stress conditions. In such a case, photosynthetic chain elements become over-reduced (Edreva 2005). When the electron transfer reactions to further acceptors are limited, ^1^O_2_ is formed in PS II reaction centre due to the photosensitizing action of excited Chl (Krieger-Liszkay 2005). Photosensitized ^1^O_2_ production was also observed in isolated antennae complexes (Triantaphylidès and Havaux 2009). Excessive reduction of photosynthetic chain elements leads to increased electron leakage resulting in O_2_^•−^ formation. This leakage occurs mostly from Fe-S clusters of PS I and reduced ferredoxin (which is called Mehler reaction), but it also takes place at the receptor side of PS II (Edreva 2005; Gechev et al. 2006). The process of energy spill-over from PS II to PS I, triggered by the reduction of the PQ pool was proposed to limit ^1^O_2_ generation in red algae (Kowalczyk et al. 2013; Fu et al. 2020). There has been no direct experimental evidence to date for ^1^O_2_ generation in *Euglena* species. This was postulated to result from an effective antioxidant mechanism, in particular the high content of carotenoids and tocopherols (Toc) in these microorganisms (Ishikawa et al. 2017). The rate of H_2_O_2_ generation in chloroplasts isolated from *Euglena* was similar to that observed in chloroplasts from higher plants (Ishikawa et al. 2017).

Mitochondria, which are considered to be the main source of ROS in animal cells, are thought to play a minor role in photosynthetic organisms. Although, it is believed that their participation in ROS formation may be dominant in the dark and in non-green tissues (Navrot et al. 2007). Major ROS type produced in these organelles is O_2_^•−^ formed as a result of electron leakage from the respiratory electron transport chain, particularly at the level of respiratory Complexes I and III. What is more, when the ubiquinone pool is over-reduced, ubiquinol may directly reduce O_2_ to O_2_^•−^ (Navrot et al. 2007). The presence of alternative oxidase in the majority of photosynthetic eukaryotes is thought to play a role in limiting ROS production in the respiratory electron transport chain because this enzyme transfers electrons directly from ubiquinol to O_2_ (Ishikawa et al. 2017). Other mitochondrial enzymes, such as monoamine oxidase and nicotinamide adenine dinucleotide phosphate oxidase, are also known to produce ROS, but their contribution to total mitochondrial ROS levels are significantly lower than that resulting from the activity of the electron transport chain (Zhang et al. 2020).

Various enzymatic reactions occurring in peroxisomes result in H_2_O_2_ formation. In particular, this ROS is the product of a reaction catalysed by glycolate oxidase, a key enzyme of the photorespiratory pathway. Another process resulting in H_2_O_2_ production is β-oxidation of fatty acids. Peroxisomes may also be a source of O_2_^•−^ (Del Río et al. 2006). Many algae contain carbon concentration mechanisms, which enable more effective CO_2_ binding and limit photorespiration (Barrett et al. 2021). Similarly to plants, their peroxisomes perform β-oxidation and contain various oxidases (Ugya et al. 2020). Photorespiration in euglenids differs from that of higher plants. These microorganisms possess glycolate dehydrogenase in mitochondria instead of peroxisomal glycolate oxidase; what is more, glyoxylate is processed in another way in further steps of the pathway (Ishikawa et al. 2017).

In many sites in plant cell, O_2_^•−^ and H_2_O_2_ can be formed enzymatically what plays different roles, such as response to pathogen or cell wall lignification (Nowicka and Kruk 2013). These types of ROS are also produced in the cell walls of algae. The production of H_2_O_2_ in the endoplasmic reticulum of microalgae results from the activity of protein disulphide isomerase (Ugya et al. 2020).

Interestingly, ROS production in microalgae depends on the cell size. Larger species generate more ROS and the direct relationship between cell size and the amount of produced O_2_^•−^ was shown. The amount of generated ROS is also dependent on density and growth phase. It is more pronounced when the cell density is low. Due to increased metabolic activity, microalgae tend to produce more ROS during the exponential phase of growth than in the other stages (Ugya et al. 2020). Many phytoplankton species produce extracellular ROS, which is thought to play a role both in inter-species interactions, as well as in algal growth and development (Hansel and Diaz 2021).

### Destructive action of ROS on cell components

The most important biomolecules vulnerable to oxidative damage are proteins, DNA, and lipids. In the case of proteins, ROS can oxidize amino acid residues, as well as cofactors and prosthetic groups, such as pigments, haems, Fe-S clusters and many others (Nowicka and Kruk 2013). The oxidation of Cys groups may lead to the inactivation of certain proteins, as well as to changes in their tertiary and quaternary structure. Changes in protein structure may also result from the oxidation of other amino acid residues. Protein damage very often leads to the loss of enzyme activity. Other consequences are aggregation or degradation of proteins, fragmentation of peptide chains, or the formation of bonds between proteins and other biomolecules (Kohen and Nyska 2002).

Oxidation of DNA results in modification of nitrogenous bases and sugar residues, loss of purines, and strand breakage (Kohen and Nyska 2002). It is worth mentioning that chloroplasts and mitochondria, where the majority of ROS is formed, contain their own genetic material not associated with histones, which makes it even more easily accessible for oxidative compounds. Studies concerning oxidative damage of algal DNA have shown that both the sugar moieties and the bases were prone to oxidation by ROS, in particular by OH^•^ (Ugya et al. 2020).

Damage to lipids occurs due to lipid peroxidation. Molecules particularly susceptible to oxidation are those containing polyunsaturated fatty acid residues (PUFA) (Niki 2009). Such residues are important for maintaining sufficient membrane fluidity; therefore, they are present in high amounts in biological membranes. Lipid peroxidation changes membrane properties reducing its fluidity and increasing its permeability, which leads to the disturbance of its organization and to functional loss (Nowicka et al. 2013). What is more, secondary products of this process, such as aldehydes, hydroxydialdehydes, ketones, cyclic peroxides, epoxides, ethers, and isoprostanes may cause damage to proteins or DNA. A well-known example of such a reaction is cross-linking of proteins by malonyldialdehyde (MDA) (Kohen and Nyska 2002; Halliwell 2006; Niki 2009). There are three different mechanisms of lipid peroxidation, i.e., nonenzymatic free radical-mediated, nonenzymatic free radical-independent, and enzymatic one. Free radical-mediated lipid peroxidation can be ﻿initiated by OH^•^, HO_2_^•^, carbon-centred radicals (R^•^), RO^•^, ROO^•^, NO_2_^•^ and perferryl radicals. It is a self-propagating chain reaction; therefore, even low amounts of radical initiators have the potential to cause extensive damage (Nowicka et al. 2013). Lipid radicals, which are intermediates of radical lipid peroxidation, may also react with membrane proteins (Nowicka and Kruk 2013). Free radical-independent nonenzymatic peroxidation is a direct reaction of ^1^O_2_ with PUFA leading to the formation of lipid hydroperoxides and cyclic peroxides (Niki 2009). In higher plants, ^1^O_2_ was reported to be responsible for over 80% of nonenzymatic lipid peroxidation in leaves (Triantaphylidès et al. 2008). Lipid hydroperoxides, similarly to H_2_O_2_, may react with metal ions resulting in the formation of RO^•^ (Niki 2009).

## Cellular antioxidants

### Hydrophilic low-molecular-weight antioxidants

The antioxidant function of ascorbate (Asc) has been extensively studied. In plant cells, this compound is the most abundant water-soluble antioxidant; in leaves, its concentration may be 5–10 times higher than that of GSH (Smirnoff 2005; Kaur and Nayyar 2014). Asc is present in the cytosol, plastids, mitochondria, peroxisomes, nucleus, vacuole, and apoplast (Gechev et al. 2006; Gest et al. 2013). In higher plants, the intracellular concentration of Asc ranges from 20 mM in the cytosol to 20–300 mM in chloroplasts, which may contain up to 30–40% of the cellular Asc pool (Triantaphylidès and Havaux 2009; Ahmad et al. 2010). Peroxisomes are another compartment with high Asc content, while vacuoles contain the lowest concentration of this antioxidant (Zechmann 2018). Asc can be synthesized in a few different pathways. Plants are known to have more than one, in the case of algae, the occurrence of a certain pathway depends on the clade (Kaur and Nayyar 2014). The D-mannose/L-galactose pathway occurs in green and red algae, whereas D-galacturonate pathway is present in algae with secondary plastids: cryptophytes, haptophytes, stramenopiles, and euglenids (Tamaki et al. 2021). Cyanobacteria either do not synthesize Asc or contain very low amounts of it, green algae and lower plants contain minor amounts of this antioxidant, whereas the highest Asc content was observed in higher plants. Particularly high Asc levels occur in the leaves of alpine plants, where Asc may constitute up to 19% of the leaf carbon pool (Gest et al. 2013). Considering algae, Asc was detected in the examined species of green and red algae, as well as in euglenids, cryptophytes, haptophytes, diatoms, and some other clades of photosynthetic stramenopiles (Brown and Miller 1992; Bilodeau and Chevrier 1998). *Euglena* was shown to accumulate millimolar concentrations of Asc (Ishikawa et al. 2017). The content of this antioxidant varied depending on a species, but also on the growth phase (Brown and Miller 1992). No Asc was detected in glaucophyte *Cyanophora paradoxa* (Wheeler et al. 2015).

At physiological pH, Asc is predominantly present in the form of ascorbate anion, which readily loses an electron from its *ene*-diol group (Smirnoff 2005). Therefore, Asc is an effective reductant able to directly scavenge O_2_^•−^, H_2_O_2_, ROO^•^, and ^1^O_2_ (Sirikhachornkit and Niyogi 2010; Ahmad et al. 2010). It is a reducing cofactor of H_2_O_2_ detoxifying enzyme ascorbate peroxidase (APX) (Hajiboland 2014). The rate of the reaction catalysed by APX is orders of magnitude higher than the rate of direct scavenging of H_2_O_2_ by Asc (Tamaki et al. 2021). Asc also plays a role in the regeneration of lipophilic antioxidants, such as Toc and carotenoids (Smirnoff 2005). Asc oxidation product, monodehydroascorbate (MDHA) is a radical stabilized by delocalisation of electrons around the central carbon ring and its three carbonyl groups (Gest et al. 2013). MDHA can disproportionate into Asc and dehydroascorbate (DHA). Both MDHA and DHA can be re-reduced enzymatically by the respective reductases (Ahmad et al. 2010). Under normal physiological conditions, Asc constitutes the majority of total ascorbate pool (Asc + MDHA + DHA) (Hajiboland 2014). In the case of inactivation of the oxygen-evolving complex in PS II, Asc may function as an alternate electron donor that slows down donor side-induced photoinactivation of PS II (Kaur and Nayyar 2014). On the other hand, Asc may also act as a pro-oxidant, for example, it can reduce Fe^3+^ and Cu^2+^ (Smirnoff 2005). Apart from participation in antioxidant defence, Asc is a cofactor of enzymes, such as violaxanthin de-epoxidase important for photoprotection in higher plants and some algae (Noctor 2006; Ahmad et al. 2010). Other examples of Asc-dependent enzymes are those participating in ethylene, gibberellin, flavonoids, and hydroxyproline biosynthesis (Kaur and Nayyar 2014). Asc is a precursor for the synthesis of oxalate and tartrate (Waśkiewicz et al. 2014a). It plays a role in redox sensing and regulation of plant growth and development (Noctor 2006; Gest et al. 2013). The role of Asc in plant stress response has been widely examined (Venkatesh and Park 2014). The application of an inhibitor of Asc biosynthesis to *Scenedesmus quadricauda* supressed an increase in Asc content in Cd-exposed algae and enhanced oxidative symptoms (Kováčik et al. 2017).

GSH is a tripeptide (γ-Glu-Cys-Gly) not synthesized on ribosomes. It is the major low-molecular-weight thiol in both prokaryotes and eukaryotes, where it often represents the major pool of nonprotein reduced S (Mallick and Mohn 2000; Sirikhachornkit and Niyogi 2010). Glutathione biosynthetic pathway is conserved in all biological kingdoms (Tamaki et al. 2021). In higher plants, GSH is synthesized in the cytosol and chloroplasts. This compound was detected in the cytosol, endoplasmic reticulum, nucleus, plastids, mitochondria, peroxisomes, vacuole, and apoplast (Gechev et al. 2006; Banerjee and Roychoudhury 2019). The highest GSH concentration (1–4 mM) occurs in chloroplasts (Ahmad et al. 2010). Under physiological conditions, this compound predominantly exists in a reduced form, while under oxidizing conditions it forms glutathione disulphide (GSSG). GSSG is reduced back to GSH by glutathione reductase (GR) (Sirikhachornkit and Niyogi 2010). The thiol group of Cys makes GSH prone to react with electrophiles and to bind metal ions (Ahmad et al. 2010). This compound is considered crucial for antioxidant defence. It scavenges H_2_O_2_, O_2_^•−^, ^1^O_2_ and organic radicals (Ahmad et al. 2010; Pikula et al. 2019). It is a reducing cofactor of several enzymes involved in ROS detoxification, such as DHA reductase necessary for Asc recycling, and GSH peroxidase (GPX) detoxifying H_2_O_2_ and lipid hydroperoxides (Gechev et al. 2006; Banerjee and Roychoudhury 2019). GSH is also a cofactor used for enzymatic reduction of oxidized thiol groups in proteins (Waśkiewicz et al. 2014b). It is a transport form of Cys and the main storage form of reduced nonprotein S. This compound is also a precursor for phytochelatin biosynthesis (Noctor 2006; Hajiboland 2014). What is more, glutathione-S-transferases (GST) catalyse the conjugation of GSH to xenobiotics and endogenous metabolites. GSTs comprise a diverse group, which members vary in their activities and functions. Some of them are crucial for intracellular detoxification processes, others play a role in the transport of flavonoids, signalling and ROS scavenging (Banerjee and Roychoudhury 2019). GSH may be posttranslationally conjugated to proteins that prevents proteolysis under oxidative stress and plays a role in signalling. It was postulated that glutathionylation is driven by the higher production of ROS (Sirikhachornkit and Niyogi 2010). In *Chlamydomonas reinhardtii*, 10 Calvin cycle enzymes were S-glutathionylated in response to oxidative stress, which is thought to be a mechanism of regulation of Calvin cycle under oxidative stress conditions (Zaffagnini et al. 2012). The glutathionylation was shown to play a role in the regulation of triacylglycerols accumulation in Cd-exposed *Auxenochlorella protothecoides* (Xing et al. 2021). Apart from being reductant and co-substrate, GSH is known to be involved in cellular signalling, playing a role in redox sensing in certain cell compartments (Foyer and Noctor 2005). The signalling function of GSH/GSSG couple is related to stress response, but also to the regulation of growth and development (Szőllősi 2014). GSH also participates in NO signalling as it reacts with NO to form S-nitrosoglutathione, a compound considered to be a stable transport form of NO (Foyer et al. 2005). The accumulation of GSH is commonly observed in plants under stress (Waśkiewicz et al. 2014b). Euglenids are known to contain GSH derivative called trypanothione (Škodová-Sveráková et al. 2020).

The accumulation of free Pro often occurs during the response of higher plants and green algae to various stress factors including toxic concentrations of heavy metal ions (Zhang et al. 2008). Pro content increased in red alga *Gracilaria corticata* during the response to salt stress and in heat-treated *Gracilaria tenuistipitata* (Chang and Lee 1999; Kumar et al. 2010a). Exposure to salt stress resulted in an induction of antioxidant response and an increase in Pro content in green microalgae *Chlorococcum humicola* and *Chlorella vulgaris* (Singh et al. 2018; Yun et al. 2019). The intracellular Pro level was also enhanced in diatom *Nitzschia palea* exposed to toxic concentrations of Cd^2+^ and Cu^2+^, and in brown alga *Ectocarpus siliculosus* exposed to salt stress. In diatoms, the accumulation of Pro was less pronounced than in green algae treated with Cd^2+^ and Cu^2+^ (Wu et al. 1995; Dittami et al. 2011). Pro is an osmolyte important for the protection of plants exposed to drought or salt stress. This imino acid was also proposed to function as a metal chelator and molecular chaperone stabilizing protein structure (Liang et al. 2013; Hossain et al. 2014). Pro accumulation was postulated to play a role in buffering cytosolic pH, balancing cell redox status, and storing C and N (Verbruggen and Hermans 2008). Pro is considered to be a regulatory molecule able to activate molecular or physiological responses (Zhang et al. 2008). Exogenously added Pro was shown to enhance the antioxidant response in plant cells exposed to various stress factors, such as salt, heat, or Cd^2+^ (Hossain et al. 2014; Rejeb et al. 2014). Under stress conditions, transgenic plants and *C. reinhardtii* with enhanced Pro synthesis displayed the increased activity of antioxidant enzymes, increased content of GSH and Asc, and decreased level of MDA when compared to stressed controls (Siripornadulsil et al. 2002; Hossain et al. 2014). Apart from the stimulation of the antioxidant response, Pro is able to directly scavenge ^1^O_2_ and radicals (Rejeb et al. 2014). This imino acid was shown to effectively scavenge organic radicals generated in vitro, but not O_2_^•−^ (Kaul et al. 2008). Scavenging of H_2_O_2_ by Pro is possible, but very slow when compared to the reactions with other low-molecular-weight antioxidants, therefore, Pro is not considered to contribute significantly to cellular H_2_O_2_ detoxification (Liang et al. 2013). In higher plants, Pro occurs in cytosol, mitochondria, and chloroplasts (Das and Roychoudhury 2014). It can be synthesized in two pathways, glutamate cycle and ornithine cycle (Meena et al. 2019).

**Fig. 3:**
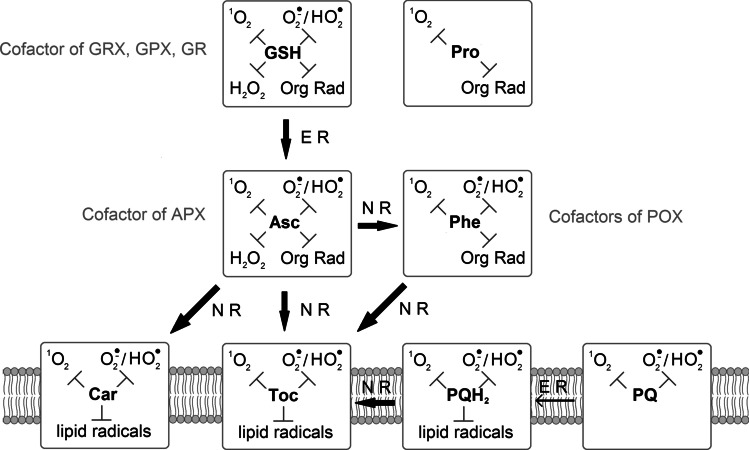
Major hydrophilic and hydrophobic low-molecular-weight antioxidants occurring in photosynthetic organisms and the ability of these compounds to detoxify ROS and organic radicals. Thick arrows symbolize participation in non-enzymatic (NR) or enzymatic (ER) regeneration of other antioxidants. Narrow arrow symbolizes enzymatic reduction of PQ to PQH_2_. Cellular localization of particular compounds and enzymes was described in the text. All the compounds shown react with OH^•^. APX, ascorbate peroxidase; Asc, ascorbate; Car, carotenoids; GPX, glutathione peroxidase; GR, glutathione reductase; GRX, glutaredoxin; GSH, glutathione; Org Rad, organic radicals; Phe, phenolic compounds; POX, peroxidase using phenolic compound as a reductant; PQ, plastoquinone; PQH_2_, plastoquinol; Pro, proline; Toc, tocopherol

Antioxidant functions are also displayed by phenolic compounds. These are diverse secondary metabolites including more than 8000 compounds divided into 10 groups (Martins et al. 2016; Rezayian et al. 2019). In in vitro systems, polyphenols were reported to be more effective in the scavenging of free radicals, both organic and inorganic, than Toc and Asc. Antioxidant properties of phenolic compounds arise from the ability to donate an electron or hydrogen atom. The resulting polyphenol-derived radicals are relatively stable due to the delocalisation of the unpaired electron (Rice-Evans et al. 1997). They may be re-reduced enzymatically or non-enzymatically by Asc (Szőllősi 2014). The antioxidant potential of phenolic compounds depends on the number and the arrangement of hydroxyl groups in their molecules (Fernandez-Panchon et al. 2008). Phenolics may chelate transition metal ions (Rice-Evans et al. 1997). Binding of metal ions and scavenging of radicals such as O_2_^•−^ and ROO^•^ enable phenolic compounds to inhibit lipid peroxidation. However, under certain conditions, i.e., high phenolics concentration, high pH, and the presence of redox-active heavy metals, these compounds may behave as pro-oxidants (Ahmad et al. 2010). Phenolic compounds are also able to quench ^1^O_2_ and are involved in H_2_O_2_ detoxification (Gechev et al. 2006; Triantaphylidès and Havaux 2009). In cells of higher plants, phenolics occur in many compartments. Apart from vacuoles and the cell wall, these compounds are present in the cytosol, endoplasmic reticulum, chloroplasts, and nucleus (Agati et al. 2012). The participation of phenolic compounds in plant stress responses has been widely documented (Agati et al. 2012). Considering other photosynthetic eukaryotes, it was shown that microalgae contain phenolics belonging to subgroups of flavonoids, such as isoflavones, flavanones, flavanols and dihydrochalcones. Usually, the content of these compounds was lower or equal to the minimum amounts found in land plants (Rezayian et al. 2019). However, in some species of microalgae: *Chlorella* sp., *Desmodesmus* sp., *Dunaliella* sp. (green algae), *Nannochloropsis* sp., and *Phaeodactylum* sp. (stramenopiles), phenolic compounds were shown to be the major contributors to the total antioxidant activity (Safafar et al. 2015). The content of phenolic compounds was significantly correlated with the antioxidant activities in diatoms *Chaetoceros calcitrans*, *Skeletonema costatum*, *Odontella sinensis*, *Phaeodactylum tricornutum*, and haptophyte *Isochrysis galbana* (Foo et al. 2017). Nowadays, phenolic compounds are thought to play a role in the antioxidant protection of algae and in the formation of an adaptive response to oxidative stress. The presence of phenolic compounds in microalgae depends on the medium used and growth conditions. The pathways of their biosynthesis in microalgae are being investigated (Zolotareva et al. 2019). Brown algae contain many polyphenols such as phlorotannins, which can constitute up to 25% of their dry biomass. These compounds very efficiently bind divalent metal ions, therefore they may reduce the toxicity of certain heavy metals (Rezayian et al. 2019; Zolotareva et al. 2019). Seasonal variations of phlorotannin content were observed in brown alga *Cystoseira foeniculacea*. These compounds were the most abundant in summer, which probably is related to the response to increased temperature and light exposure (Kozak et al. 2020). Red algae are capable of accumulating large amounts of polyphenols, including bromophenols, which protect these organisms from being eaten but also display antioxidant properties. Bromophenols were also found in species belonging to brown and green algae (Zolotareva et al. 2019). The major low-molecular-weight antioxidants are shown in Fig. 3.

Other low-molecular-weight hydrophilic compounds have been reported to function as antioxidants, for example, it was shown that vitamin B_6_ can efficiently scavenge ^1^O_2_ (Triantaphylidès and Havaux 2009). Dimethylsulphoniopropionate and its enzymatic cleavage product dimethylsulphide were postulated to play antioxidant functions in marine microalgae such as diatoms and coccolithophores (Sunda et al. 2002). Ovothiol, a histidine-derived thiol with antioxidant properties was found in *Euglena* (Ishikawa et al. 2017). Compounds belonging to mycosporin-like amino acids occur in a wide variety of marine organisms including algae. Their main function is protection from UV, but some of them have antioxidant properties (Coulombier et al. 2021).

### Lipophilic low-molecular-weight antioxidants

Lipophilic antioxidants belong to the groups of isoprenoid chromanols, isoprenoid quinols, and carotenoids (Fig. 3). Isoprenoid chromanols and quinols are amphipathic compounds, which molecules are comprised of a polar head group (a chromanol or quinol ring, respectively) and an apolar prenyl side–chain that anchors them in lipid bilayers (Nowicka and Kruk 2010; Szymańska et al. 2017). These properties make isoprenoid chromanols and quinols crucial for the protection of membranes and lipid storage sites (Kruk et al. 2016).

The most important and most common isoprenoid chromanols are Tocs and tocotrienols (T_3_s), the former containing a fully saturated isoprenoid side-chain derived from phytyl diphosphate and the latter containing an unsaturated chain derived from geranylgeranyl diphosphate (Szymańska et al. 2017). According to the distribution of methyl substituents in the chromanol ring, we distinguish α, β, γ, and δ forms of Toc and T_3_. Isoprenoid chromanols with longer, nonaprenyl side-chains were also discovered. These are plastochromanol (PC-8) and its oxidized derivative hydroxy-plastochromanol (Kruk et al. 2014). The predominant Toc form in cyanobacteria and higher plants is α-Toc. This compound was also shown to occur in the examined green, red, and brown algae (Antia et al. 1970; Sánchez-Machado et al. 2002; Nowicka et al. 2020). Some isoprenoid chromanols (like γ-Toc or PC-8) occur in minor amounts in leaves. The main source of other Toc forms and T_3_s are seeds, where the chromanol composition depends on the species. In higher plants chromanols are synthesized and occur in plastids; in seeds they can also be found in oleosomes (Szymańska et al. 2017). The results of the experiments on *Euglena gracilis* suggest that this protozoan is able to synthesize α-Toc both in chloroplasts and mitochondria (Kusmic et al. 1998). Considering their antioxidant properties, the most extensively studied compounds are Tocs, examined in this respect both in vitro and in vivo. It was shown that under certain conditions α-Toc is able to scavenge O_2_^•−^, to quench and scavenge ^1^O_2_, and to efficiently inhibit lipid peroxidation by scavenging lipid radicals (Mène-Saffrané and DellaPenna 2010; Kruk et al. 2016). Tocopheroxyl radicals, formed as a result of radical scavenging, can be re-reduced non-enzymatically to the corresponding Tocs by Asc, isoprenoid quinols or phenolic compounds (Kruk et al. 2016). Other isoprenoid chromanols, among them PC-8, also display pronounced antioxidant properties (Nowicka et al. 2013; Kruk et al. 2014). The participation of Tocs in the acclimation of higher plants to various stress factors has been widely documented (Munné-Bosch 2005). The content of Toc in algae varies depending on a species (Jayasree et al. 1985; Safafar et al. 2015).

Concerning isoprenoid quinones, the majority of the research focused on their function in photosynthesis and respiration. The quinone ring can undergo two-step reversible reduction and protonation leading to a quinol form, which makes these compounds very useful as electron and proton carriers in various electron transport chains. Isoprenoid quinones also play a role as enzyme cofactors and in signalling. Photosynthetic eukaryotes contain PQ pool in their plastids and ubiquinone in their mitochondria (Nowicka and Kruk 2010). Apart from the above-mentioned roles, these compounds are effective antioxidants, especially in their reduced, quinol form. Similar to Tocs, they are able to inhibit lipid peroxidation, quench and scavenge ^1^O_2_, and scavenge inorganic free radicals, such as O_2_^•−^ or perferryl radical (Gruszka et al. 2008; Nowicka and Kruk 2010; Nowicka et al. 2013; Kruk et al. 2016). What is more, they play a role in Toc recycling (Nowicka et al. 2013). Radical scavenging leads to the formation of semiquinone forms, which may disproportionate to quinols and quinones (James et al. 2004). The latter is effectively re-reduced enzymatically (Nowicka and Kruk 2010). Quinone forms also display antioxidant properties, such as scavenging of ^1^O_2_ and O_2_^•−^; however, they are less pronounced when compared to those displayed by quinols (Gruszka et al. 2008; Nowicka and Kruk 2010; Nowicka et al. 2013; Kruk et al. 2016).

There are more than 750 different carotenoids found in nature. In photosynthetic organisms, the main function of these compounds is light harvesting and photoprotection (Nowicka and Kruk 2016). Carotenoids belong to terpenoids and usually are synthesized by condensation of 8 isoprenoid units. Shorter compounds made of 6 units were discovered in heliobacteria. Carotenoids are subdivided into carotenes, which are hydrocarbons, and xanthophylls containing in their molecules also oxygen atom(s). Carotenoids differ in the degree of saturation, the presence or absence of ring(s) at their ends, and the presence and distribution of various substituents (Nowicka and Kruk 2016). Algae belonging to different clades vary with their carotenoid composition, therefore these pigments are valuable chemotaxonomic biomarkers (Takaichi 2011; Tamaki et al. 2021). Most microalgae contain β-carotene and zeaxanthin. Green algae have also lutein, neoxanthin, and violaxanthin, which is similar to higher plants, as well as other carotenoids, such as loroxanthin, siphonaxanthin, and astaxanthin. Macrophytic red algae contain lutein as their major carotenoid, whereas in unicellular red algae zeaxanthin dominates. Fucoxanthin and diadinoxanthin are the major carotenoids in stramenopiles. These pigments also occur in haptophytes; however, diadinoxanthin is not as widespread as in the former group. Peridinin is a characteristic xanthophyll of dinoflagellates. Cryptophytes contain α-carotene and unique acetylene xanthophylls. Euglenids synthesize pigments characteristic for green lineage, but also those present in heterokonts, such as diadinoxanthin (Mc Gee and Gillespie 2019; Tamaki et al. 2021). Carotenoids occur in plastids. In chloroplasts, they are bound to photosystems and antennae, but some fraction diffuses freely in membranes. These pigments are known to modify membrane fluidity and enhance its stability. Antioxidant properties of carotenoids are based on their ability to quench ^1^O_2_ and scavenge ROO^•^ and O_2_^•−^. What is more, these compounds are able to quench the triplet excited states of Chl that prevents ^1^O_2_ formation (Ahmad et al. 2010; Latowski et al. 2014; Tamaki et al. 2021). Some carotenoids have been reported to be more effective in ROO^•^ and OH• scavenging than α-Toc. In microalgae able to accumulate lipid droplets, carotenoids have been also shown to create a sunscreen layer of oil droplets (Zhang et al. 2020). It is thought that carotenoids contribute significantly to the total antioxidant capacity of microalgae (Safafar et al. 2015; Foo et al. 2017).

### Major antioxidant enzymes responsible for direct ROS detoxification

SODs are enzymes able to detoxify O_2_^•−^; therefore, they are thought to be the first line of antioxidant enzymatic defence in cells. As these enzymes dismutate O_2_^•−^ to H_2_O_2_ and O_2_, they do not need any additional reductant (Rezayian et al. 2019). SODs are a group of metalloisoenzymes classified depending on the metal ion(s) in their active centre into Cu/ZnSOD, MnSOD, FeSOD, and NiSOD. The last type was discovered in bacteria belonging to *Streptomyces* and cyanobacteria, whereas the other types are more widely distributed (Barondeau et al. 2004; Habibi 2014). Additionally, so-called cambialistic SODs were discovered in some archaeans. These enzymes are able to use both Fe or Mn as a prosthetic group, depending on the availability of certain metal ions (Wolfe-Simon et al. 2005). Recently, copper-only SOD, phylogenetically related to Cu/ZnSOD, was discovered in some fungi and oomycetes (Peterson et al. 2016). FeSOD and MnSOD usually are homodimers or homotetramers, Cu/ZnSODs are homodimeric or monomeric, while NiSODs are hexamers. FeSOD and MnSOD are considered to be more ancient types than Cu/ZnSOD, because the first two types widely occur both in eukaryotes and prokaryotes, while the third one has been detected predominantly in eukaryotes (Barondeau et al. 2004; Szőllősi 2014). FeSOD and MnSOD are structurally similar to each other and most probably have arisen from the same ancestral enzyme. It was hypothesized that FeSOD is the most ancient type, while MnSOD evolved when the raise of O_2_ in the environment led to the decrease in Fe^2+^ availability (Alscher et al. 2002). Different SOD types vary in sensitivity to certain inhibitors, i.e., Cu/ZnSODs are inhibited by CN^−^ and H_2_O_2_, FeSODs are inhibited by H_2_O_2_, while MnSODs are not inhibited by the above-mentioned compounds (Mallick and Mohn 2000; Habibi 2014). Cu/ZnSODs are very stable (Wolfe-Simon et al. 2005). In higher plants, Cu/ZnSODs are localized in the cytosol, chloroplasts, and peroxisomes, while FeSODs were found in chloroplasts of some species. MnSODs occur in mitochondria and peroxisomes (Alscher et al. 2002; Sirikhachornkit and Niyogi 2010; Ahmad et al. 2010). *A. thaliana* genome contains three FeSOD genes, three Cu/ZnSOD genes, and one MnSOD gene (Habibi 2014).

Cyanobacteria typically contain NiSOD alone or combinations of NiSOD and MnSOD or FeSOD and MnSOD (Wolfe-Simon et al. 2005). Land plants and charophycean algae contain Cu/ZnSOD, FeSOD, and MnSOD, while the other green algal clades are thought to have FeSOD and MnSOD only, localized in chloroplasts and mitochondria, respectively (Wu et al. 2009). Interestingly, NiSOD homologue was found in the genome of green microalga *Ostreococcus tauri* (Schmidt et al. 2009). MnSOD was isolated from red algae *Porphyridium cruentum* and *Porphyra yezoensis* (Grace 1990). Red algae, as well as diatoms, are thought to retain MnSOD as their sole SOD type. In the examined diatom genomes, pseudogenes displaying homology to genes encoding FeSOD were found. There are data concerning FeSOD occurrence in haptophytes (Wolfe-Simon et al. 2005). Recently, occurrence of Cu/ZnSOD homologues in the genome of red alga *Gracilariopsis chorda* was reported. It was also suggested that SOD-encoding genes of brown algae have multiple origins and are much more diversified than those of green and red algae (Liu and Wang 2020). The presence of Cu/ZnSOD, FeSOD, and MnSOD was reported for dinoflagellates (Okamoto and Colepicolo 1998; Wang et al. 2019). Literature data supports the occurrence of two SOD types in *E. gracilis*, FeSOD and MnSOD, the latter was found in the thylakoid fraction of the cell extract (Kanematsu and Asada 1979). An increase in SOD activity was observed both in higher plants and algae during the response to various stress factors (Mallick and Mohn 2000; Wolfe-Simon et al. 2005; Ahmad et al. 2010; Cruces et al. 2017).

CATs are widely distributed among aerobes, and they also occur in some anaerobic organisms (Mallick and Mohn 2000; Mhamdi et al. 2010). CATs are the only enzymes able to directly dismutate H_2_O_2_ to O_2_ and H_2_O, therefore they do not need any additional reductant (Mallick and Mohn 2000). CATs have high V_max_ and turnover numbers, but their affinity to the substrate is low when compared to APX or PRXs. Therefore, CATs are considered to be crucial for H_2_O_2_ detoxification in the situation when this ROS is formed in high amounts (Feierabend 2005; Mhamdi et al. 2010). There are three major groups of CATs:

1. Heme-containing, usually tetrameric “typical” monofunctional CATs
2. Heme-containing bifunctional catalase-peroxidases phylogenetically related to APX, found in some archaea, bacteria, and fungi
3. Non-heme Mn-containing CATs occurring in archaea and bacteria (Feierabend 2005; Whittaker 2012).

Heme-containing CATs are inhibited by O_2_^•−^. This inhibition is reversible, however, under certain conditions, such as high O_2_^•−^ concentration, enzyme cannot quickly revert to the active form and is inactivated. CAT inactivation may also occur when the enzyme is exposed to very high H_2_O_2_ concentrations (Feierabend 2005). When H_2_O_2_ concentration is low, “typical” CAT may reduce H_2_O_2_ and oxidize other substrates, such as methanol, ethanol, Asc, formaldehyde, and formic acid (Mallick and Mohn 2000; Ahmad et al. 2010). The increase in CAT activity is often observed during the stress response (Feierabend 2005).

Higher plants generally contain multiple forms of CAT (Feierabend 2005). Plant CATs are present predominantly in peroxisomes, where they detoxify H_2_O_2_ released during photorespiratory glycolate oxidation and by some other reactions, as well as in glyoxysomes, where H_2_O_2_ is formed during β-oxidation of fatty acids. These enzymes were also found in mitochondria. There were reports concerning CAT activity in apoplast and chloroplasts; however, in the latter case, the purity of the obtained fractions was questioned. CAT of yeast *Saccharomyces cerevisiae* was reported to be present in mitochondria, peroxisomes, and cytosol (Feierabend 2005; Mhamdi et al. 2010). Cyanobacteria contain a typical and bifunctional CATs, the presence of a certain type depends on a species. Homologues of Mn-containing CATs were also found in the genomes of these prokaryotes (Mhamdi et al. 2010; Whittaker 2012). CAT encoding genes were found in examined members of green and red algae (Škodová-Sveráková et al. 2020). *C. reinhardtii* has one CAT gene and synthesizes three CAT isoforms, which are dimeric and were reported to be localized in mitochondria (Kato et al. 1997; Michelet et al. 2013). However, the most recent results of the experiments with confocal imaging show that *C. reinhardtii* CAT isoforms are rather targeted to peroxisomes and endoplasmic reticulum (Kato et al. 2021). Phylogenetic analyses showed that euglenids studied so far do not have genes encoding CAT (Škodová-Sveráková et al. 2020). CAT activity was measured in six species of marine diatoms, but there were also reports on the lack of CAT activity in certain diatom species (Feierabend 2005; Nguyen-Deroche et al. 2012; Manimaran et al. 2012; Anu et al. 2016). The genes of bifunctional CATs were found in analysed genomes of stramenopiles including diatom and brown algal species (Zámocký et al. 2012). CAT activity was measured in the marine brown macroalga *Padina tetrastromatica* (Maharana et al. 2010). In the mixotrophic dinoflagellate *Prorocentrum micans* CAT homologue was expressed under conditions favouring autotrophic growth (Shim et al. 2011).

APX detoxifies H_2_O_2_ using Asc as a reductant. Two Asc molecules are oxidized to MDHA during the reduction of one H_2_O_2_ to H_2_O (Mallick and Mohn 2000). APX is a heme peroxidase belonging to the same superfamily as cytochrome *c* peroxidase and bifunctional catalase-peroxidases (Maruta et al. 2016). APX has a high affinity to H_2_O_2_, therefore it is considered to be involved in the modulation of ROS signalling (Ahmad et al. 2010). In higher plants, APX participates in water-water cycle in chloroplasts and Asc-GSH cycles in those cell compartments which contain also reductases of MDHA, DHA and GSH (Ahmad et al. 2010). As plant chloroplasts do not contain CAT, APX is considered to be the main H_2_O_2_ detoxifying enzyme in these organelles (Sirikhachornkit and Niyogi 2010). Plant APXs include thylakoid and microsomal (i.e., present in peroxisomes and glyoxysomes) membrane-bound forms and stromal, mitochondrial, cytosolic and apoplastic soluble forms (Ahmad et al. 2010; Imahori 2014). In some species thylakoid and stromal APXs are products of the same gene undergoing alternative splicing, in other species they are encoded by different genes (Mittler and Poulos 2005). Plant APXs usually are homodimers. APX izoenzymes differ in molecular weight, stability and optimal pH. Chloroplastic types of APX need Asc for their stability and are very sensitive to inactivation (Ahmad et al. 2010; Imahori 2014). *A. thaliana* genome contains nine genes for APX, rice contains eight, while tomato has seven (Gechev et al. 2006; Gest et al. 2013). In higher plants, the expression of APX is relatively high even in optimal conditions and it is dramatically enhanced during the response to almost all biotic and abiotic stresses studied (Mittler and Poulos 2005). The pattern of expression changes varies depending on the APX isoenzyme and the type of stress (Ishikawa and Shigeoka 2008; Caverzan et al. 2012; Anjum et al. 2016).

APX is absent in prokaryotes, but it is widely distributed in plants and eukaryotic algae (Ishikawa and Shigeoka 2008; Gest et al. 2013). Genomes of green and red algae contain APX homologues, usually one gene per species (Maruta et al. 2016). Red algae *Galdieria partita* and *G. sulphuraria* contain two cytosolic APXs (Rezayian et al. 2019). *C. reinhardtii* contains three APX isoforms, among them APX1 and APX2 were predicted to be dual-targeted to chloroplasts and mitochondria, while APX4 is thought to be chloroplastic enzyme (Kuo et al. 2020). One APX was found in *C. vulgaris* (Takeda et al. 1998). Diatoms, brown algae, and cryptophytes were reported to contain APX-encoding genes. In most cases, these algae contain one gene per species, but there are exceptions, i.e., the cryptophyte *Guillardia theta* and diatom *P. tricornutum* have two genes of APX. Most probably one of the pair is targeted to the chloroplast and another to the cytosol (Maruta et al. 2016). APX was found in members of dinoflagellates and euglenids, in the latter group, this enzyme is localized in the cytosol (Gest et al. 2013). Passardi et al. (2007) reported the occurrence of APX-encoding genes in the examined genomes of glaucophytes, haptophytes and chlorarachniophyta, but in the paper by Maruta et al. (2016), these clades were described to lack APX. This discrepancy probably results from methodology used. Maruta et al. excluded genes with sequences changed in sites crucial for APX activity. The authors explained that this was the reason for the exclusion of APX-like protein found in chlorarachniophyte species with known genomes (Maruta et al. 2016). In algae, the expression of APX and other H_2_O_2_ detoxifying enzymes was shown to depend on the availability of micronutrients needed for the synthesis of a certain type of enzyme (Ishikawa and Shigeoka 2008). Both APX activity and the efficiency of enzymatic processes of Asc recycling were shown to play a role in the tolerance of *C. reinhardtii* to high light (Yeh et al. 2019; Kuo et al. 2020). Constitutive high activity of APX and GR, as well as large Asc pool were observed in Antarctic alga *Chlamydomonas* sp. UWO 241 (Stahl-Rommel et al. 2021).

### Other enzymes involved in ROS detoxification

**Fig. 4:**
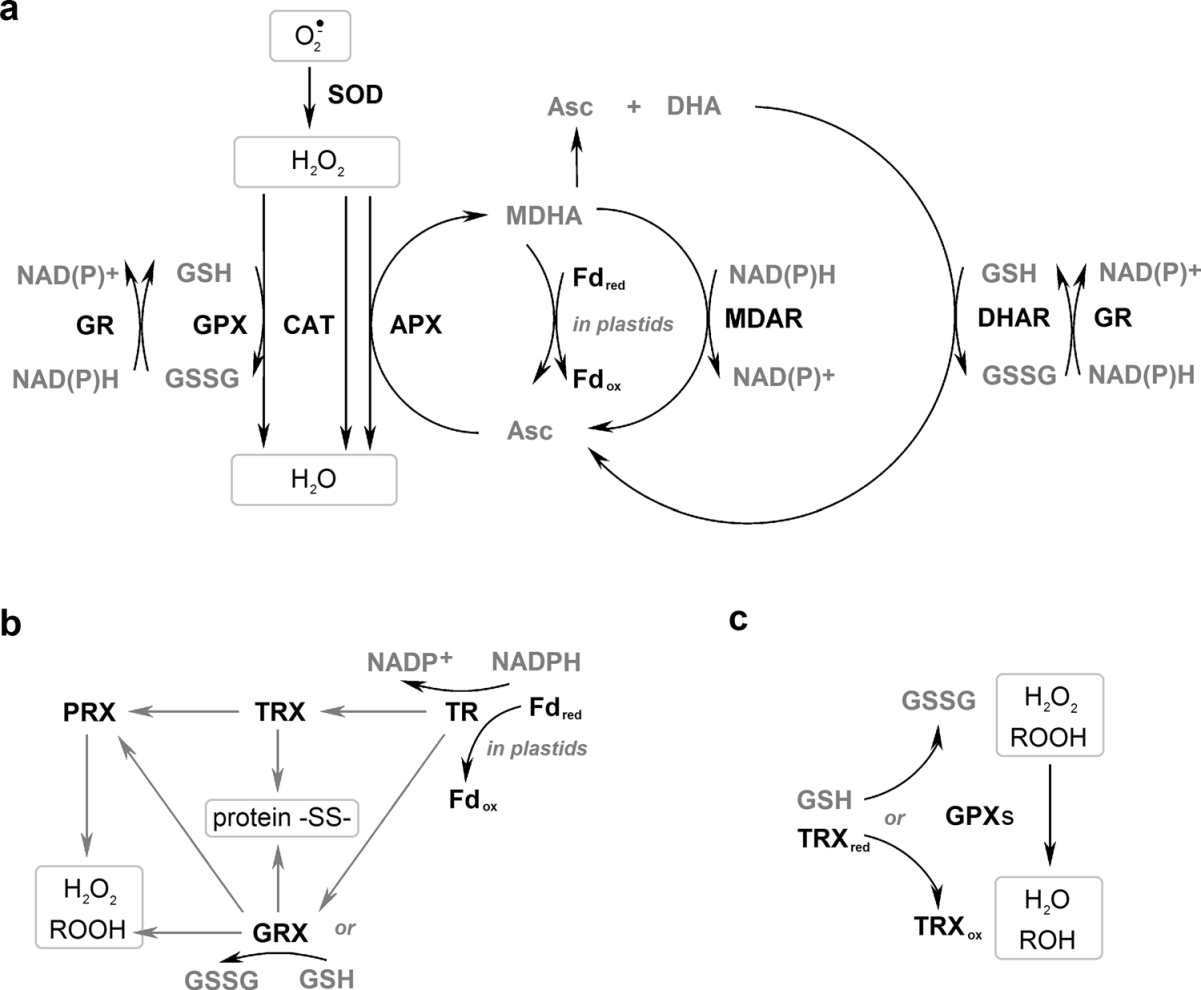
Major reactive oxygen species-detoxifying enzymes and recycling of their cofactors (**a**), thioredoxin-peroxiredoxin-glutaredoxin system (**b**), and the versatility of reactions catalysed by various glutathione peroxidases (**c**). Enzyme cofactors are marked by grey font. Grey arrows are used to show reduction of peroxides or oxidized thiol groups of proteins. APX, ascorbate peroxidase; Asc, ascorbate; CAT, catalase; DHA, dehydroascorbate; DHAR, dehydroascorbate reductase; Fd_red_, reduced ferredoxin, Fd_ox_, oxidized ferredoxin; GPX, glutathione peroxidase; GR, glutathione reductase; GRX, glutaredoxin; GSH, glutathione; GSSG, glutathione disulphide; MDHA, monodehydroascorbate; MDHAR, monodehydroascorbate reductase; PRX, peroxiredoxin; SOD, superoxide dismutase; TRX, thioredoxin; TR, thioredoxin reductase

ROS-detoxifying enzymes are shown in Fig. 4. Apart from SODs, CATs, and APX, there is also a wide array of other enzymes involved in antioxidant defence, including glutathione peroxidases (GPXs) and proteins belonging to thioredoxins (TRXs), peroxiredoxins (PRXs) and glutaredoxins (GRXs), which occur both in prokaryotes and eukaryotes (Rouhier and Jacquot 2002; Lemaire 2004).

GPXs are a family of multiple isozymes catalysing the reduction of H_2_O_2_ and ROOH to water and alcohol, respectively, using GSH or TRX as a reductant, depending on the GPX type. GPXs are classified into three types: selenium-dependent GPX, nonselenium-dependent phospholipid hydroperoxide GPX, and GSTs showing glutathione peroxidase activity. Enzymes belonging to these classes differ in their structures and catalytic mechanisms (Ahmad et al. 2010; Wakao and Niyogi 2021). In higher plants, a number of cysteine-containing enzymes were found, while selenium-dependent ones are rare. GPXs containing Cys in their active site have lower activities than those containing selenocysteine (Sirikhachornkit and Niyogi 2010; Anjum et al. 2012). *A. thaliana* genome encodes eight GPXs, while *C. reinhardtii* has five, among which one displays enhanced expression in response to ^1^O_2_ and high light. Among GPXs of *C. reinhardtii*, two (GPX1 and 2) belong to selenium and three (GPX3-5) to non-selenium types (Sirikhachornkit and Niyogi 2010; Tamaki et al. 2021). In higher plants, these enzymes occur in the cytosol, chloroplasts, mitochondria, endoplasmic reticulum, peroxisomes, and apoplast (Gechev et al. 2006; Anjum et al. 2012). Plant GPXs were postulated to participate not only in ROS scavenging, but also in redox signalling (Passaia and Margis-Pinheiro 2015). *C. reinhardtii* enzymes are thought to be targeted to mitochondria, chloroplasts and cytosol (Tamaki et al. 2021). *Euglena* genome contains four GPX homologues, one GPX isolated from this protozoan was shown to occur in cytosol (Ishikawa et al. 2017).

TRXs are small ubiquitous redox proteins that reduce disulphide bridges of their numerous target proteins by thiol-disulphide exchange reactions. TRXs often play regulatory roles, but they may also protect thiol-containing proteins enabling their re-reduction. What is more, TRXs can act as electron donors for PRXs. Higher plants synthesize several different TRXs, while *C. reinhardtii* contains eight, *Euglena* eleven, and cyanobacterium *Synechocystis* sp. contains four genes encoding these enzymes (Sirikhachornkit and Niyogi 2010; Ishikawa et al. 2017). In eukaryotes, different TRXs are targeted to different cell compartments, such as cytosol, chloroplasts, mitochondria, nucleus, and plasma membrane (Gechev et al. 2006; Geigenberger et al. 2017). The majority of plant TRXs are located in plastids (Dos Santos and Rey 2006). These enzymes are reduced by certain thioredoxin reductases using NADPH (in extraplastidial compartments) or reduced ferredoxin (in plastids) as electron donors (Geigenberger et al. 2017).

GRXs are small redox proteins belonging to the thioredoxin superfamily (Lemaire 2004). Similar to TRXs, *A. thaliana* genome contains much more GRX encoding genes (above 30) than that of *C. reinhardtii* (8) and *Synechocystis* sp. (3) (Sirikhachornkit and Niyogi 2010). Wider analysis carried out by Couturier et al. (2009) showed that the genomes of examined higher plants contained 30–40 GRXs, the examined moss and lycophyte contained 15 and 16 GRX genes, respectively, examined algae (eight species of green algae, one red alga, one haptophyte, and two diatom species) had 4 to 10 GRXs depending on a species, while in cyanobacteria (38 genomes analysed) there were 2 to 7 GRX genes per species (Couturier et al. 2009). *Euglena* genome was shown to contain 12 GRX-encoding genes (Ishikawa et al. 2017). These enzymes are categorized into two major groups on the basis of the number of Cys residues in their active site. Later, six GPXs classes have been distinguished on the basis of their sequences. Their distribution differs in various systematic groups. Phylogenetic analyses of known genomes showed the occurrence of GRX classes I, II, V, and VI in cyanobacteria (usually one species contains two or three GPX genes); classes II and IV in eukaryotic algae; and classes I, II, III, and IV in land plants (Couturier et al. 2009; Mondal et al. 2020). GRXs catalyse thiol-disulphide redox reactions using GSH as a reductant via monothiol or dithiol mechanism depending on the enzyme type. Many GRXs may also be re-reduced by thioredoxin reductases (Couturier et al. 2009). Some GRXs may reduce DHA, ROOH, and PRXs. GRXs are also thought to play a role in deglutathionylation and in the assembly of Fe-S clusters (Rouhier et al. 2008; Sirikhachornkit and Niyogi 2010). In higher plants, most of these enzymes are present in the cytosol, but they also occur in chloroplasts, mitochondria, endoplasmic reticulum, and plasmalemma (Dietz 2005; Gechev et al. 2006).

PRXs are enzymes structurally similar to TRXs. They are able to reduce H_2_O_2_, ROOH, and peroxynitrite. The role of PRXs in redox signalling has been also postulated (Dietz 2005). PRXs were subdivided into four types on the basis of their sequence and catalytic mechanisms. In their active sites, PRXs contain thiol groups, which undergo oxidation to sulphenic acid followed by a reaction leading to the formation of a disulphide bridge. The active form of PRX is regenerated by interactions with TRXs, GRXs, or cyclophilins (Sirikhachornkit and Niyogi 2010). *A. thaliana* genome contains ten PRX genes. A similar number of homologues were found in *Populus trichocarpa* and *Oryza sativa*, while in cyanobacterium *Synechocystis* sp. PCC6803 there were four PRX genes. In the brown alga *E. siliculosus* only one PRX gene was found (Dietz 2011). *C. reinhardtii* contains seven PRXs, probably targeted to chloroplasts, cytosol, and mitochondria (Dayer et al. 2008). Four PRX genes were found in *Euglena*; their products are supposed to be localized in the cytosol, chloroplasts and mitochondria (Ishikawa et al. 2017). In higher plants, PRXs are present in cytosol, plastids, mitochondria, and nucleus (Gechev et al. 2006; Sirikhachornkit and Niyogi 2010). The most abundant PRX isoform belongs to plastid targeted ones (Dos Santos and Rey 2006). Some PRXs are bound to thylakoid membranes (Sirikhachornkit and Niyogi 2010).

Other antioxidant enzymes worth mentioning are lipid hydroperoxide reductase occurring in the chloroplast envelope and guaiacol peroxidases (Gechev et al. 2006; Sirikhachornkit and Niyogi 2010). The latter occurs in the cytoplasm, mitochondria, vacuoles, and cell walls of higher plants. They are heme-containing enzymes able to detoxify H_2_O_2_ using various substrates (preferentially aromatic compounds) as reductants. These peroxidases can also produce ROS. They play various roles; i.e., they participate in lignin biosynthesis and pathogen defence (Gechev et al. 2006; Hajiboland 2014). Euglenids contain a unique H_2_O_2_ detoxification system based on glutathione analogue called trypanothione, trypanothione reductase, tryparedoxin, and tryparedoxin peroxidase (Škodová-Sveráková et al. 2020).

### Enzymes participating in ascorbate and glutathione recycling

Asc and GSH are crucial for ROS detoxification; therefore, enzymes participating in their recycling are very important elements of antioxidant defence. There are numerous routes of Asc regeneration. MDHA can be re-reduced by reduced ferredoxin (in plastids), by cytochrome *b*_561_ (in plasmalemma and tonoplast), and by monodehydroascorbate reductase (MDHAR). MDHARs are FAD-containing enzymes using NADPH or NADH as electron donors (Smirnoff 2005; Noctor 2006). In higher plants, these enzymes were found in the cytosol, mitochondria, chloroplasts, peroxisomes, and plasma membrane (Smirnoff 2005). Plastidic and mitochondrial MDHARs use NADPH, while enzymes found in the cytosol and plasmalemma use NADH (Mittler et al. 2004; Khan et al. 2011). MDHARs can also reduce phenoxyl radicals (Smirnoff 2005). *A. thaliana* and tomato genomes contain five and two genes of MDHAR, respectively (Noctor 2006; Gest et al. 2013). MDHAR activity was detected in cyanobacteria, but not in all strains examined. It was also reported to occur in green algae *Dunaliella salina* and *C. vulgaris*, and in the red alga *G. partita* (Gest et al. 2013). *C. reinhardtii* contains one MDHAR, most probably localized in the cytosol (Yeh et al. 2019). *E. gracilis* is thought to have one MDHAR present in the cytosol (Shigeoka et al. 1987b). MDHAR-encoding genes were found in many (but not all) green and red algae, and in brown alga *E. siliculosus*. They were absent in analysed genomes of diatoms, haptophytes, cryptophytes, glaucophytes, and chlorarachniophytes (Wheeler et al. 2015). One has to remember that in the case of some algal clades, genomes have been sequenced only for one or a few representatives.

If MDHA is not reduced rapidly, it spontaneously disproportionates to Asc and DHA. The latter may be reduced to Asc by DHA reductase (DHAR) using GSH as a reductant. The ability to reduce DHA to Asc is also displayed by some GRXs and GSTs (Noctor 2006). Other thiol-containing enzymes were also reported to reduce DHA (Smirnoff 2005). *A. thaliana* contains five genes of DHAR, which products are targeted to cytosol, mitochondria, and chloroplasts (Gechev et al. 2006). DHAR activity was discovered in some cyanobacteria and the above-mentioned algae *D. salina* and *G. partita*, but not in *C. vulgaris* (Gest et al. 2013). *E. gracilis* and *C. reinhardtii* were reported to have one DHAR; in *Euglena* species it is localized in the cytosol, while the enzyme of green alga most probably is targeted to chloroplasts (Shigeoka et al. 1987b; Lin et al. 2016). Considering DHARs, analysis of genomes showed the occurrence of their genes in some green and red algae, diatoms, *E. siliculosus,* and cryptophyte *G. theta*. No DHAR homologues were found in members of glaucophytes, haptophytes, and chlorarachniophytes (Wheeler et al. 2015).

Re-reduction of oxidized GSH is catalysed by GR. GR is a highly conserved flavoprotein oxidoreductase using NADPH as an electron donor and present both in prokaryotes and eukaryotes (Ahmad et al. 2010; Rezayian et al. 2019). In higher plants, this enzyme is encoded by more than one gene: *A. thaliana* and wheat genomes contain two genes encoding GR, while in rice and *P. trichocarpa* three genes were found. Plant GRs are localized predominantly in chloroplasts, but also in the cytosol, mitochondria, and peroxisomes (Gechev et al. 2006; Rao and Reddy 2008; Ahmad et al. 2010; Anjum et al. 2012). In plant photosynthetic tissues, more than 80% of GR activity was reported to be of chloroplastic isoform (Gill et al. 2013). In *C. reinhardtii* two GRs were discovered, most probably one is localized in the plastid and one in the cytosol (Serrano and Llobell 1993). *E. gracilis* contains one GR localized in the cytosol (Shigeoka et al. 1987a). Two GR genes were found in the genome of diatom *P. tricornutum* (Arias et al. 2010). The expression of GR in higher plants and the activity of this enzyme are increased in response to various stress factors (Gill et al. 2013; Habibi 2014). It was shown that GR plays a role in the tolerance of *C. reinhardtii* to photo-oxidative stress (Lin et al. 2018).

### Indirect mechanisms playing a role in response to oxidative stress

The direct scavenging of ROS and efficient recycling of the low-molecular-weight antioxidants is crucial to provide the tolerance to oxidative stress, but there are other important, indirect mechanisms. ROS cause the oxidation of cellular compounds; therefore, there is a need for resynthesis, repair or degradation of oxidized proteins, nucleic acids and lipids. The misfolding of proteins in cells exposed to heavy metals results from both oxidation and direct interaction between metal ions and some amino acids. Thus, the protective action of heat shock proteins (HSPs) is an important element of the response to heavy metal-induced stress. HSPs play a role in accurate folding, transport and assembly of nascent proteins, restore proper structures of misfolded proteins, prevent protein aggregation and promote selective degradation of misfolded or denatured proteins (Hasan et al. 2017). Proteomic and transcriptomic analyses showed that HSPs, in particular these belonging to sHSPs, HSP60 and HSP70 classes, are induced in heavy metal-exposed plants and that there is a correlation between their accumulation and tolerance to heavy metals (Hasan et al. 2017). The results of the experiments on algae let us to conclude that HSPs play a protective role also in these organisms. The expression of genes encoding HSP70s was enhanced in diatom *Ditylum brightwellii* and dinoflagellate *Alexandrium pacificum* exposed to Cu (both species) or Ni (*A. pacificum*) (Wang et al. 2021a). An increase in the expression of the gene encoding a member of HSP90 class was observed Cu-exposed dinoflagellate *Prorocentrum donghaiense*, whereas genes encoding sHSPs were up-regulated in green algae *Closterium ehrenbergii* (Zhang et al. 2019; Abassi et al. 2019). Interesting results confirming the role of HSPs in response to oxidative stress were obtained by Sathasivam and Ki (2019), who observed an increase in the expression of the gene encoding HSP70 in green alga *Tetraselmis suecica* exposed to Cu, but not Cd.

Proteins that cannot be repaired have to be degraded. This can be achieved by ubiquitination and degradation in proteasomes or by autophagy. Both of these processes were shown to play a role in the cellular response to heavy metal-induced stress in plants (Hasan et al. 2017). Autophagy enables the degradation of aggregated proteins and whole damaged organelles. It may also play a role in the degradation of membrane transporters what leads to decreased metal ion uptake (Hasan et al. 2017). The autophagic activity was observed in Cr-exposed green alga *Dictyosphaerium* sp. and Zn-exposed *C. vulgaris* (Papini et al. 2018). Autophagy was also postulated to play a role in the recycling of lipids, as the inhibition of this process in *Chlorella zofingiensis* resulted in the decrease in total fatty acid content by about 20% (Zhang et al. 2020).

## Antioxidant response and heavy metal toxicity in algae

The results of the experiments in which algae were exposed to heavy metal ions and the antioxidant response were monitored were collected in Table 1. In the majority of cases, the application of heavy metal salts in toxic concentrations resulted in the occurrence of oxidative stress. Acclimation to heavy metal-induced stress was usually accompanied by the increase in the content of low-molecular-weight antioxidants and the activity of antioxidant enzymes (Table 1). Usually, the higher the heavy metal salt concentration applied, the more pronounced increase in antioxidant content or enzyme activity was observed, but only to the certain threshold, depending on the sensitivity of examined species to the metal tested. Application of heavy metal salt concentrations high enough to cause severe stress results in antioxidants depletion (Elbaz et al. 2010; Piotrowska-Niczyporuk et al. 2015; Nowicka et al. 2016a, 2020; Cheng et al. 2016; Moussa et al. 2018; Lu et al. 2021; Ajitha et al. 2021).

**Table 1:** Oxidative stress markers and antioxidant response in algae exposed to heavy metal-induced stress. ↑, increase; ↓, decrease; no information about the trend means that there were no statistically significant differences between heavy metal-treated algae and untreated control or there were no changes in time, = mark was used when there were no differences for all samples tested. Element name in [] means concentration of heavy metal salt used in the experiment. *APX*, ascorbate peroxidase; *Asc*, ascorbate; *Car*, carotenoids; *CAT*, catalase; *Chl*, chlorophyll; *DHA*, dehydroascorbate; *DHAR*, dehydroascorbate reductase; *GPX*, glutathione peroxidase; *GR*, glutathione reductase; *GSH*, glutathione; *GST*, glutathione-S-transferase; GSSG, glutathione disulphide; *LOOH*, lipid hydroperoxide; *MDHAR*, monodehydroascorbate reductase; *PC-8*, plastochromanol-8; *POD*, peroxidase; *PQ*, plastoquinone; *PQH*_*2*_, plastoquinol; *PQ*_*tot*_, sum of PQ and PQH_2_; *Pro*, proline; *PRX*, peroxiredoxin; *ROS*, reactive oxygen species; *SOD*, superoxide dismutase; *TBARS*, thiobarbituric acid-reactive substances being lipid peroxidation products; *α-Toc*, α-tocopherol; *γ-Toc*, γ-tocopherol; *TRX*, thioredoxin

Species	Heavy metal tested	Experimental conditions	Measured markers of oxidative stress	Antioxidant response: antioxidant content and enzyme activity	Reference
**Archaeplastida: green algae**					
*Chlamydomonas reinhardtii*	Hg, Cd	CdCl_2_ 120 µM HgCl_2_ 5 µM Samples taken at t = 0, 3, 8, 18, 24, 45 h of exposure		Total soluble thiols: ↑ later ↓, for Hg maximum obtained after 3 h, for Cd after 24 h GSH: ↑ for the first 3 h, then ↓ in Hg-exposed algae	Howe and Merchant (1992)
*C. reinhardtii*	Cd	CdCl_2_ 150 µM Exposure time 4–5 d		Amount of APX, MnSOD, GST: ↑ Amount of FeSOD: ↓ Amount of chloroplast PRXs: some ↑, some ↓	Gillet et al. (2006)
*C. reinhardtii*	Hg	HgCl_2_ 1, 2, 4, 6, 8 µM Exposure time: 30 min for ROS formation, 24 h for enzyme activity, 96 h for TBARS measurements. Pro measured at t = 0, 60, 120, 180, 240 min	ROS formation, TBARS: ↑	CAT, APX, SOD: ↑ and ↓ for highest [Hg] applied Pro measurements in time: ↑ during 1^st^ h of exposure then ↓	Elbaz et al. (2010)
*C. reinhardtii*	Hg	HgCl_2_ 4 µM Exposure time: 30 min for ROS formation, 6 h for other measurements	ROS formation, TBARS: ↑	Pro, SOD, APX: ↑ CAT: =	Wei et al. (2011)
*C. reinhardtii*	Cu	CuSO_4_ 10, 50, 100, 150, 200 µM Exposure time 2 d	Lipid peroxidation measured independently as thermoluminescence and TBARS: ↑	α-Toc: ↑ then ↓ for 100 µM and higher [Cu] CAT: ↓ for 150 µM and higher [Cu] SOD: =	Luis et al. (2006)
*C. reinhardtii*	Cu	CuSO_4_ 5 µM Exposure time: 30 min for ROS formation, 6 h for enzyme activity, 24 h for TBARS measurements	ROS formation, TBARS: ↑	SOD, CAT: ↑ APX: =	Zheng et al. (2011)
*C. reinhardtii*	Cu	CuSO_4_ 50, 100, 250 µM Samples taken after 1, 3, 5 d of exposure	TBARS: ↑ For 250 µM [Cu] ↑ in TBARS content in time was observed, for other [Cu] applied, the highest TBARS level was observed after 3 days of exposure	SOD, GST, GPX: ↑ for 100 and 250 µM [Cu] POD: similar trend to other enzymes, except 100 µM [Cu] after 3 d Usually there were no changes in GPX and GST activity in time, SOD activity ↓ between 3 and 5 d, POD activity ↑ between 3 and 5 d GSH content: ↑ for 100, 250 µM [Cu] in samples taken after 1 and 5 d; ↓ after 3 d	Jiang et al. (2016)
*C. reinhardtii*	Ag, Cd, Cr, Cd, Hg	CuSO_4_, K_2_Cr_2_O_7_ 5, 10, 20, 50 µM CdCl_2_ 10, 20, 40, 100 µM HgCl_2_ 1, 3, 5, 10 µM AgNO_3_ 3, 5, 10 µM Exposure time 14 d	LOOH: ↑ in Cr- and Cd-exposed algae	Car/Chl ratio: slight ↑ in Cr-exposed algae α-Toc: ↑ in Cr- and Cd-exposed algae; ↓ in Ag-exposed algae; ↑ then ↓ for the highest [Cu] and for 5, 10 µM [Hg] in Cu- and Hg-exposed algae, respectively γ-Toc: ↑ in Cu- Cr- and Cd-exposed algae; ↓ in Ag-exposed algae; ↑ then ↓ for highest [Hg] in Hg-exposed algae PQ_tot_: ↑ in Cd-, Cr- and Hg-exposed algae; ↓ in Ag-exposed algae Share of PQH_2_ in PQ pool: ↑ in Cd-, Cr- and Hg-exposed algae; ↓ in Ag- and Cu- exposed algae	Nowicka et al. (2016a)
*C. reinhardtii*	Ag, Cd, Cr, Cd, Hg	CuSO_4_, K_2_Cr_2_O_7_, CdCl_2_ 200 µM HgCl_2_, AgNO_3_ 20 µM Samples taken at t = 0, 2.5, 5, 7.5 h	LOOH: ↑ in Cu-, Cr-, Hg-, Ag-exposed algae	Car, γ-Toc, PC-8, PQ_tot_: ↓ in Ag- and Cu- exposed algae α-Toc: ↓ in Ag-, Cr- and Cu-exposed algae Share of PQH_2_ in PQ pool: rapid ↓ in Ag-, Cr- and Cu-exposed algae; ↓ in Cd- and Hg-exposed algae	Nowicka et al. (2016b)
*C. reinhardtii*	Cr, Cd	K_2_Cr_2_O_7_ 4, 8 µM CdCl_2_ 22, 26 µM Exposure time 14 d	O_2_^•−^: ↑, more pronounced in Cr-exposed algae	Car/Chl ratio, α-Toc, PQ_tot_, share of PQH_2_ in PQ pool, PC-8, Asc, total soluble nonprotein thiols, Pro, APX: ↑ γ-Toc: ↑ in Cr-exposed algae CAT: ↑ in algae exposed to 8 µM [Cr] SOD: ↑ in Cr-exposed, ↓ in Cd-exposed algae	Nowicka et al. (2020)
*C. reinhardtii*	Cu	CuSO_4_ 20, 25 µM Exposure time 14 d Samples taken at t = 7, 10, 14 d	TBARS: ↑ after 7 days of exposure	Car/Chl ratio: ↓ for 25 µM [Cu] after 7 d α-Toc, γ-Toc, PQ_tot_, PQH_2_: ↓ PC-8: ↑ after 7 d; ↓ after 10, 14 d Asc + DHA: ↑ after 7 d; ↓ after 10 d; ↑ for 25 µM [Cu] after 14 d DHA: ↑, the most significant after 7 d Total soluble nonprotein thiols: ↓ after 10 d Pro: ↓ after 10 d for 25 µM [Cu] SOD: ↑ after 7 d for 20 µM [Cu]; ↓ after 7, 10 d for 25 µM [Cu] CAT: ↑ after 7 d for 20 µM [Cu]; ↓ after 14 d APX: ↑ after 7 d for all [Cu], for 20 µM [Cu] after 10 d; ↓ for 20 µM [Cu] after 14 days	Nowicka et al. (2021)
*C. reinhardtii*	Ni	NiSO_4_ 60, 90, 120, 125, 130, 135 µM, exposure time 6 d for TBARS measurements 125 µM NiSO_4_, exposure time: 8 h for ROS formation, 48 h for measurements of antioxidants	TBARS: ↑ for 90 µM and higher [Ni] H_2_O_2_, O_2_^•−^: ↑	Pro, nonprotein thiols: ↑ Asc: ↓	Zheng et al. (2013)
*C. reinhardtii*	Ag	Ag nanoparticles 1, 5, 10, 30, 50 mg/dm^3^ Exposure time 24, 48, 72 h	ROS formation: ↑ for 5 mg/dm^3^ and higher [Ag] after 24 h, for 10 mg/dm^3^ and higher [Ag] after 48, 72 h TBARS: ↑ for 5 mg/dm^3^ and higher [Ag]	SOD: ↑ for 30, 50 mg/dm^3^ [Ag] after 24 h, for 5 mg/dm^3^ and higher [Ag] after 48 h, for all [Ag] after 72 h SOD activity ↓ in time for all the series including control POD: ↑ for 50 mg/dm^3^ [Ag] after 24 h, for 30, 50 mg/dm^3^ [Ag] after 72 h There was significant ↑ in POD activity for 50 mg/dm^3^ [Ag] after 24 h and then ↓	Zhao et al. (2021)
*Chlamydomonas acidophila*	Cd, As	CdCl_2_ 0.1, 0.25, 0.5, 0.75, 1, 5, 10, 25, 50 µM NaAsO_2_ 0.1, 0.5, 1, 2.5, 5, 10, 20 µM Na_2_HAsO_4_ 0.1, 0.5, 1, 2.5, 5, 10, 20, 30, 50 µM Exposure time 24 h	O_2_^•−^: ↑ in As-exposed cells, more pronounced for As (III) treatment for 10 and 20 µM series		Díaz et al. (2020)
*Chlorella* sp.	Cd, Cu	CuSO_4_ 5, 10, 20, 40, 80, 160 µM CdSO_4_ 5, 10, 20, 40, 80, 160, 200 µM Exposure time 24 h		Pro: ↑ For 160 µM [Cu] the increase was less pronounced than for 40 and 80 µM [Cu]	Wu et al. (1995)
*Chlorella sorokiniana, Scenedesmus acuminatus*	Cu	CuCl_2_ 25, 50 µM Exposure time 7 d	H_2_O_2_, TBARS: ↑	APX, GR, SOD, GSH, Asc, polyphenols, Pro, Toc: ↑ GSH/(GSH + GSSG), Asc/(Asc + DHA) ratios: ↓ POD: ↑ in *S. acuminatus* GST, flavonoids: ↑ in *C. sorokiniana*	Hamed et al. (2017a)
*C. sorokiniana, S. acuminatus*	Zn	ZnCl_2_ 0.6, 1 mM Exposure time 7 d	H_2_O_2_, TBARS: ↑	SOD, GSH, Asc, Toc: ↑ Asc/(Asc + DHA) ratio: ↓ GR, GST, flavonoids, polyphenols: ↑ in *C. sorokiniana* Pro: ↑ in *S. acuminatus* POD, APX: slight ↑ marked as statistically not significant	Hamed et al. (2017b)
*C. sorokiniana*	Cd	Salt type not given 0.025, 0.05, 0.1, 0.2, 0.4 mg/dm^3^ Samples taken after 48, 72, 96 h	O_2_^•−^: ↑ for all [Cd] after 48, 72 h, for the highest [Cd] after 96 h TBARS: ↑ for 0.05 mg/dm^3^ and higher [Cd] after 48 h, for all [Cd] after 72, 96 h Changes in time: O_2_^•−^ ↓ and TBARS ↑ then ↓ in Cd-exposed algae	GSH: ↑ SOD: ↑ except the lowest [Cd] after 72 h CAT: ↑ for 0.05 mg/dm^3^ and higher [Cd] after 48, 72 h, for 0.4 mg/dm^3^ [Cd] after 96 h Changes in time: CAT and GSH ↓ in all series including controls; SOD ↓ for 0.05 mg/dm^3^ and higher [Cd]	Wang et al. (2018b)
*C. sorokiniana*	Cd, Cu, As	CuCl_2_ 500 µM CdCl_2_ 250 µM NaAsO_2_ 750 µM Na_2_HAsO_4_ 10 mM Exposure time 42 h		APX: ↓ in Cu and Cd-exposed algae; ↑ in As exposed algae CAT: ↑	León-Vaz et al. (2021)
*Chlorella vulgaris*	Cu	CuCl_2_ 0.25, 0.5, 1, 2, 2.5, 3 mg/dm^3^ Exposure time 72 h	TBARS: ↑ in all except the lowest [Cu]	Car: ↑ in all except the lowest [Cu] GSH: ↓ in all except the lowest [Cu] Pro: ↑ for 1 mg/dm^3^ and higher [Cu] Asc, APX: ↓ for 1 mg/dm^3^ and higher [Cu] CAT, GR: ↓ for 2 mg/dm^3^ and higher [Cu] SOD: ↑ for 2 mg/dm^3^ and higher [Cu]	Mallick (2004)
*C. vulgaris*	Cr, Cu, Ni, Zn	CuCl_2_ 2.5 µM K_2_Cr_2_O_7_ 5 µM NiCl_2_ 15 µM ZnSO_4_ 30 µM Samples taken at t = 0, 2, 4, 6, 8, 12, 18, 24 h For TBARS measurements, [heavy metals] 10 µM, exposure time 1 h	TBARS: ↑	Pro: ↑ in Ni- and Zn-treated algae; ↑ for the first 4 h then ↓ in Cu-treated algae; ↑ for the first 8 h then ↓ in Cr-treated algae	Mehta and Gaur (1999)
*C. vulgaris*	Cr	K_2_Cr_2_O_7_ 0.01, 0.1, 1, 5, 10, 15, 25, 50, 100 µg/cm^3^ Exposure time 120 h	TBARS: ↑, the most pronounced for 15 µg/cm^3^ [Cr]	Car: ↑ for 0.1–25; ↓ for 50, 100 µg/cm^3^ [Cr] SOD: ↑ for 0.01–5, the most pronounced for 1 µg/cm^3^ [Cr]; ↓ for 15 µg/cm^3^ and higher [Cr] CAT: ↑ APX, guaiacol peroxidase: ↑ for 0.1 µg/cm^3^ and higher [Cr], in the case of APX the most pronounced for 10 µg/cm^3^ [Cr]	Rai et al. (2013)
*C. vulgaris*	Cd, Cu	CdCl_2_ 1, 2 µM CuSO_4_ 0.5, 1.5 µM Applied alone or together in all combinations of concentrations Exposure time 48 h	TBARS: ↑ for 1.5 µM [Cu] and all the series with Cu + Cd	SOD: ↑ for 1.5 µM [Cu] applied alone or together with Cd POD: ↑ for 1.5 µM [Cu] applied alone or together with Cd, for 0.5 µM [Cu] + 1 µM [Cd] CAT: ↑ for 1.5 µM [Cu] + 1 µM [Cd]	Qian et al. (2011)
*C. vulgaris*	Cu, Cd, Zn	Cu: 2.5 ppm Cd: 5 ppm Zn: 50 ppm Salt type not given. Exposure time 7 d	TBARS: ↑, most pronounced for Cu-treatment	CAT, POD: ↑, most pronounced for Cu-treatment	El-Naggar and Sheikh (2014)
*C. vulgaris*	Cu	CuCl_2_: 0.2, 2, 5, 10, 25, 50 µM, exposure time 2 and 72 h for ROS measurements 1, 2, 3, 4, 5 µM, exposure time 72 h for other measurements	ROS formation, H_2_O_2_: ↑ for all [Cu] after 2 h, for 5 µM and higher [Cu] after 72 h OH^•^: ↑ for 10 µM and higher [Cu] after 2 h, for 5 µM and higher [Cu] after 72 h TBARS: ↑ for 2, 3, 4 µM [Cu]; ↓ for 5 µM [Cu]	SOD: ↑, most pronounced for 2, 3, 4 µM [Cu] CAT: ↑ for 2; ↓ for 5 µM [Cu]	Chen et al. (2016)
*C. vulgaris*	Cr	K_2_Cr_2_O_7_ 0.5, 1, 2, 5 mg/dm^3^ Exposure time 12 d	TBARS: ↑	SOD: ↑ for 0.5, 1; ↓ for 2, 5 mg/dm^3^ [Cr] CAT: ↑ for 0.5, 1; ↓ for 5 mg/dm^3^ [Cr]	Lu et al. (2021)
*C. vulgaris*	Cd	CdSO_4_ 0.5, 1, 3, 5, 7 mg/dm^3^ Exposure time 18 d	H_2_O_2_, O_2_^•−^: ↑ for two highest [Cd]	Car: ↓ for two highest [Cd] CAT: ↑ for 0.5, 1, 3; ↓ for 5, 7 mg/dm^3^ [Cd] SOD: ↑ for 0.5; ↓ for 5, 7 mg/dm^3^ [Cd] GR: ↑ for 0.5, 1; ↓ for 7 mg/dm^3^ [Cd] POD: ↑	Cheng et al. (2016)
*C. vulgaris*	Cd	CdCl_2_ 0.5, 1.5, 3, 5 mg/dm^3^ For SOD and POD measurements samples taken after 2, 4, 6 d TBARS and CAT measured after 6 d	TBARS: ↑	SOD: ↑ POD: ↑ for 0.5, 1.5, 3; ↓ for 5 mg/dm^3^ [Cd] CAT: ↑ for 3, 5 mg/dm^3^ [Cd] Changes in time: SOD ↑ in Cd-exposed algae, POD ↑ for 0.5 – 3 mg/dm^3^ [Cd] and ↓ between 4 and 6 d for the highest [Cd]	Geng et al. (2021)
*C. vulgaris*	Hg, Zn	ZnCl_2_ 0.06, 0.12, 0.24, 0.48, 0.96, 1.92 mM HgCl_2_ 1.9, 3.8, 7.6, 15.2, 30.4, 60.8 µM Applied alone or together Samples taken after 48 h and 7 d ROS formation measured for 0.06, 0.48, 1.92 mM [Zn] and 1.9, 15.2, 60.8 µM [Hg]	ROS formation: ↑	CAT: ↑ SOD: ↑ for three lowest and ↓ for the highest [Zn], [Hg], [Zn + Hg] after 48 h; ↑ for the lowest [Zn], [Hg], [Zn + Hg] and ↓ for the three highest [Zn] [Hg], [Hg + Zn] applied after 7 d	Ajitha et al. (2021)
*Chlorella pyrenoidosa*	Cu	CuSO_4_ 0.18, 0.58, 1.08, 5.08, 10.08 mg/dm^3^ Exposure time 96 h	TBARS: ↑ for two highest [Cu]	SOD: ↑ for two highest [Cu]	Lu et al. (2015)
*C. pyrenoidosa*	Ni	NiCl_2_ 1, 5, 10, 20 mg/dm^3^ Samples taken after 1, 4, 7 d	ROS formation: ↑	SOD: ↑ for 5 mg/dm^3^ and higher [Ni] after 4, 7 d CAT: ↑ for 1 mg/dm^3^ [Ni] after 1 d and ↓ after 4, 7 d; ↑ for 5 mg/dm^3^ and higher [Ni] after 1, 4 d, for the highest [Ni] after 7 d Changes in time: SOD ↑ for 5 mg/dm^3^ and higher [Ni]; CAT ↑ for all series including control	Li et al. (2021a)
*Chlorella kessleri, Scenedesmus vacuolatus*	Cu	CuCl_2_ 6.2, 108 µM for *C. kessleri* 6.2, 108, 210, 414 µM for *C. vacuolatus* Exposure time 7 d	TBARS: ↑ in *S. vacuolatus*	SOD, CAT, GSH: ↑ in *S. vacuolatus*	Sabatini et al. (2009)
*Scenedesmus bijugatus*	Cu	CuSO_4_ 25, 50, 100 µM for enzyme activity measurements 100, 200, 400 µM for GSH and Asc measurements, exposure time 7 d 100, 200 µM for TBARS measurements, samples taken after 6 and 10 d	TBARS: ↑, more pronounced for longer exposure	APX, SOD, CAT: ↑ GPX: ↑ for 50, 100 µM [Cu] GSH, Asc: ↓	Nagalakshmi and Prasad (1998)
*S. bijugatus*	Cu	CuSO_4_ 50, 100, 200 µM Exposure time 3 d GSH level was also monitored in time at t = 0, 24, 48, 72 h For H_2_O_2_ measurements: [Cu] 0.2, 0.5, 1 mM, samples taken at t = 6, 12, 24, 48, 72 h	H_2_O_2_: ↑ Changes in H_2_O_2_ in time after Cu application: ↑ and later ↓, the decrease was smaller and occurred later for the highest [Cu] than for lower [Cu]	non-protein thiols, GSH, GR: ↓ GST, GPX: ↑ GSH changes in time: ↓, the higher [Cu], the faster	Nagalakshmi and Prasad (2001)
*Scenedesmus quadricauda*	Cd, Cu	CuCl_2_, CdCl_2_ 150 µM Exposure time 24 h	ROS: ↑ in Cu-treated algae	APX: ↑ in Cu-treated algae Car, soluble phenols: ↓ in Cu-treated algae	Štork et al. (2013)
*Scenedesmus obliquus*	Cu	CuSO_4_ 1, 2, 3 mg/dm^3^ Samples taken after 12, 24, 48 h		CAT: ↑, more pronounced for samples collected after 12 h than after 48 h APX: ↑ in all Cu-treated algae after 12, 24 h, for the highest [Cu] after 48 h GR: = GST: ↑ for 2, 3 mg/dm^3^ [Cu]	Dewez et al. (2005)
*S. obliquus*	Pb	Pb(NO_3_)_2_ 141 ppm Exposure time 18 d	H_2_O_2_, TBARS: ↑	SOD, CAT, APX, guaiacol peroxidase, GR, Pro, polyphenols: ↑ Asc: =	Danouche et al. (2020)
*S. obliquus*	Cd	CdCl_2_ 10 mg/dm^3^ Samples taken after 24, 48, 72 h		GSH changes in time: ↑ for 48 h, then ↓ SOD changes in time: ↑ for 24 h, then ↓ CAT changes in time: ↓ during 48 h for algae grown as biofilm and during 24 h for algae grown in suspension, slight ↑ for longer incubation	Ma et al. (2021)
*Scenedesmus* sp*.*	Cu, Zn	CuCl_2_ 2.5, 10 µM ZnCl_2_ 5, 25 µM Samples taken after 6 h and 7 d	TBARS: ↑	Car: = SOD: ↑ for all series except 5 µM [Zn] after 6 h CAT: ↑ for 25 µM [Zn] after 7 d, for 2.5 µM [Cu] APX: ↑ for 25 µM [Zn] after 7 d, for 2.5 µM [Cu]; ↓ for 10 µM [Cu] GR: ↓ for all algae except 5 µM [Zn] after 6 h Pro: ↑, more pronounced for Cu-treatment and more pronounced for longer exposure	Tripathi et al. (2006)
*Scenedesmus* sp*.* IITRIND2	Cd	CdCl_2_ 5, 10, 25 ppm Car level measured at 2, 4, 6, 8, 10, 12 d ROS formation and antioxidant response measured after 12 d	ROS formation, TBARS: ↑	Car: ↓ for 5, 10 ppm [Cd] after 6 d, for 10, 15 ppm after 8 d, for all Cd-exposed cells after 2, 10, 12 d Changes in time: ↓ during first 2 d then ↑ in Cd-exposed algae, ↑ during 4–10 d, than ↓ for all series including control Betaine, GR, APX, CAT: ↑ Pro: ↓ SOD: slight, statistically insignificant ↓ Total antioxidants: ↑ for 25 ppm [Cd]	Tripathi et al. (2021)
*Acutodesmus obliquus*	Pb	Pb(NO_3_)_2_ 0.01, 0.1, 1, 10, 100, 500 µM Samples taken after 1, 3, 5, 7 d	TBARS, H_2_O_2_: ↑ for all concentrations applied except 0.01 µM [Pb] after 1 d, for all [Pb] after longer exposure Both TBARS and H_2_O_2_ ↑ in time	Asc, GSH, SOD, APX, CAT, GR: ↑ for two lowest [Pb]; ↓ for 10 µM and higher [Pb] If there was an ↑, it was most pronounced on 5 d, ↓ was most pronounced after 7 d	Piotrowska-Niczyporuk et al. (2015)
*S. capricornutum*	Cu, Pb	CuSO_4_, PbCl_2_ 5, 10, 25, 50, 75, 100 µM Exposure time 32 h For 100 µM [Cu] samples were taken also at t = 0, 0.5, 1, 4, 6, 8, 10, 12 h		APX: ↑ in Cu-exposed algae; ↑ for the lower [Pb], ↓ for the highest [Pb] APX measurements in time: ↑ between 4 and 8 h after Cu application	Sauser et al. (1997)
*S. capricornutum*	Cd, Cr, Cu, Zn	CdCl_2_ 0.12, 0.5, 1.9 µM K_2_Cr_2_O_7_ 2.7, 11, 41 µM Cu(NO_3_)_2_ 0.08, 0.32, 1.3 µM ZnCl_2_ 0.15, 0.60, 2.5 µM Samples taken after 6 and 72 h	ROS formation: ↑ for the highest [Cd] and [Zn] for both incubation times, for the highest [Cu] and [Cr] after 72 h	GSH: ↑ for 0.5, 1.9 µM [Cd] after 72 h, for 1.3 µM [Cu] after 72 h, for 2.5 µM [Zn] after 72 h; ↓ for 11, 41 µM [Cr]	Machado and Soares (2016)
*S. capricornutum*	Cu, Ni, Zn	CuCl_2_ 0.093, 0.149, 0.224, 0.261, 0.373, 0,745, 1.491 mg/dm^3^ NiCl_2_ 0.099, 0.148, 0.247, 0.395, 0.494, 0.741, 1.482 mg/dm^3^ ZnCl_2_ 0.096, 0.192, 0.240, 0.408, 0.648, 0.816, 1.583 mg/dm^3^ Exposure time 96 h	TBARS: ↑ for all [Cu], for 0.247 mg/dm^3^ and higher [Ni], for 0.192 mg/dm^3^ and higher [Zn]	Total soluble thiols: ↑ for 0.149 mg/dm^3^ and higher [Cu], for 0.395 mg/dm^3^ and higher [Ni], for 0.192 mg/dm^3^ and higher [Zn]	Filová et al. (2021)
*Monoraphidium convolutum*	Cr	K_2_Cr_2_O_7_ 0.1, 0.5, 1, 5, 10 mg/dm^3^ Exposure time 5 d	TBARS: ↑ for 0.1, 0.5, 10 mg/dm^3^ [Cr]	Non-protein thiols: ↑ GSH: ↑ for all [Cr] except 0.5 mg/dm^3^ [Cr] GSSG: ↑ for 0.5, 10 mg/dm^3^ [Cr] GR: ↑ for 1, 5, 10 mg/dm^3^ [Cr] APX: ↑ for 5, 10 mg/dm^3^ [Cr]	Takami et al. (2012)
*Dunaliella salina, Dunaliella tertiolecta*	Cu	CuCl_2_ 1, 5, 10, 20 µM Exposure time 24 h	TBARS: ↑	Car, APX: ↑	Nikookar et al. (2005)
*D. salina, D. tertiolecta*	Cr	K_2_Cr_2_O_7_ 4, 10, 20, 40, 60 ppm, exposure time 48 h for TBARS and H_2_O_2_ measurements 40 ppm, exposure time 24 h for SOD and -SH groups measurements	TBARS, H_2_O_2_: ↑, more pronounced for *D. tertiolecta* Protein –SH: ↓	SOD: ↓, more pronounced for *D. tertiolecta*	Arun et al. (2014)
*Dunaliella* sp*.*	Ni	NiSO_4_ 0.64, 1.29, 1.93, 3.23 mM Exposure time 7 d	TBARS: ↑	Car, SOD, CAT, GPX: ↑	Moussa et al. (2018)
*Coccomyxa subellipsoidea*	Cd	CdCl_2_ 10, 100 µM Exposure time 24 h		GSH, GSSG, GR: ↑ for 10; ↓ for 100 µM [Cd] Asc: ↓ for 100 µM [Cd] APX: ↓	Kováčik et al. (2015)
*C. subellipsoidea*	Pb, Hg	HgCl_2_, PbCl_2_ 100 µM Exposure time 24 h	ROS: ↑, more pronounced in Hg-exposed cells	SOD, CAT: ↑ in Hg-exposed algae APX: ↑, more pronounced for Hg-treatment MDHAR, DHAR: ↑ in Pb-exposed algae Asc: ↓ in Hg-exposed algae nonprotein thiols: ↓, more pronounced for Hg-treatment	Kováčik et al. (2017)
*Ulva lactuca*	Cu	CuSO_4_ 40, 120, 300, 420, 920 µg/dm^3^ Exposure time 7 d		GPX: ↑	Jervis et al. (1997)
*U. lactuca*	Cd	CdCl_2_: 0.1, 0.2, 0.3, 0.4, 0.5, 0.6, 0.7 mM for H_2_O_2_ and TBARS measurements 0.4 mM for other measurements Exposure time 4 d	H_2_O_2_: ↑ for 0.2 mM and higher [Cd] TBARS: ↑	Car/Chl ratio, Asc + DHA, GSH + GSSG, Asc/DHA ratio, GSH/GSSG ratio, SOD, APX, GR, GPX: ↑ CAT: ↓	Kumar et al. (2010b)
*Ulva fasciata*	Cu	CuSO_4_ 5, 10, 20, 50 µM Exposure time 4 d	H_2_O_2_: ↑ for 50 µM [Cu] TBARS: ↑ for 20, 50 µM [Cu]	Asc + DHA, GSH + GSSG, Asc/DHA ratio, GSH/GSSG ratio, MnSOD: = FeSOD, APX, GR: ↑ CAT: ↑ for 10 µM and higher [Cu]	Wu and Lee (2008)
*U. fasciata*	Cd	CdCl_2_ 5, 10, 20, 50 µM Exposure time 4 d	H_2_O_2_, TBARS: =	GSH + GSSG: ↓ for 50 µM [Cd] GSH/GSSG ratio: ↓ Asc + DHA, FeSOD: slight ↓ for 5, 10; ↑ for 20, 50 µM [Cd] Asc/DHA: ↑ for 10; ↓ for 50 µM [Cd] MnSOD: = APX, GR, CAT: ↑	Wu et al. (2009)
*Ulva compressa*	Cu	CuCl_2_ 10 µM ROS formation measured after 3 and 12 h Other parameters measured at t = 0, 0.25, 0.5, 1, 2, 3, 4, 5, 6, 7 d	ROS formation: ↑ O_2_^•−^: ↑, for the first 3 d slow, 3–5 days medium rate, 5–7 the fastest	APX: ↑ after 3 d, the fastest during 5–7 d GR: ↑ GST: rapid ↑ for the 1^st^ d, then ↓ till 5 d	Gonzalez et al. (2010)
*U. compressa*	Cu	CuCl_2_ 10 µM Exposure time 3 d	TBARS: ↑	APX, GST, PRX, TRX: ↑	Contreras-Porcia et al. (2011)
*U. compressa*	Cu	CuCl_2_ 10 µM Samples taken at t = 0, 1, 3, 5, 7 d		Asc: rapid ↓ during 1 d DHA: rapid ↓ during 1 d, then ↑ GSH: ↑ until day 5 GSSG: ↑	Mellado et al. (2012)
*Tetraselmis gracilis*	Cd	Cd (salt type not given): 1.5, 3 ppm Exposure time 6 months		SOD: ↑	Okamoto et al. (1996)
**Archaeplastida: red algae**					
*Gracilaria tenuistipitata*	Cd, Cu	For TBARS determination: CuSO_4_ 0.05, 0.1, 0.2, 0.5 ppm CdCl_2_ 0.1, 0.5, 1 ppm For other measurements: CuSO_4_ 0.2 ppm CdCl_2_ 1 ppm Exposure time 4 d	TBARS: ↑ for 0.5 ppm [Cu] and 1 ppm [Cd] Protein oxidation (measured as protein carbonyls): ↑	CAT, lutein, β-carotene: ↑ SOD, APX: ↑ in Cu-treated algae	Collén et al. (2003)
*Gracilaria domingensis*	Pb, Cu	CuCl_2,_ PbCl_2_ 5, 10 ppm Exposure time 7 d		Lutein: ↓ for Cu-treated algae; ↑ for Pb-treated algae β-carotene, GR: ↓ for Cu-treated algae	Gouveia et al. (2013)
*Gracilaria lemaneiformis, Gracilaria lichenoides*	Cu	CuSO_4_ 50, 100, 250, 500 µg/dm^3^ Exposure time 6 d	ROS formation: ↑ in *G. lemaneiformis* for all [Cu], in *G. lichenoides* for two highest [Cu] TBARS: ↑ in *G. lemaneiformis* for 100, 250, 500 µg/dm^3^ [Cu], in *G. lichenoides* for two highest [Cu]	*G. lichenoides*: SOD, GR: ↑, except for the lowest [Cu] APX: ↑ for two highest [Cu] *G. lemaneiformis* SOD: ↑ for 50, 100 µg/dm^3^ [Cu]; ↓ for 500 µg/dm^3^ [Cu] APX: ↑, except for the lowest [Cu] GR: ↑ for 100, 250 µg/dm^3^ [Cu]	Huang et al. (2013)
**Haptophytes**					
*Pavlova viridis*	Cu, Zn	CuSO_4_ 0.05, 0.1, 0.2, 0.5, 1, 3 mg/dm^3^ ZnSO_4_ 0.65, 1.3, 3.25, 6.5 mg/dm^3^ Exposure time 13–15 d	TBARS: ↑ for 0.5 mg/dm^3^ and higher [Cu], for two highest [Zn]	GSH: ↑ for 0.2 mg/dm^3^ and higher [Cu], for 1.3 mg/dm^3^ and higher [Zn] SOD: ↑ for 0.2 mg/dm^3^ and higher [Cu] CAT: ↑ GPX: ↑ for 1 and 3 mg/dm^3^ [Cu]; ↓ in Zn-exposed algae	Li et al. (2006)
*Pavlova viridis*	Co, Mn	CoCl_2_, MnCl_2_ 10, 20, 50, 100, 200 µM Exposure time 13–15 d	TBARS: ↑ in Co-exposed algae	Car: ↑ for 20, 50 µM [Co], for 10, 20 µM [Mn] SOD: ↑ for 20, 100 µM [Co]; ↓ 50 µM [Co] CAT, GSH: ↑ for all [Co], for two highest [Mn] GPX: ↑ for all [Co], for two highest [Mn]; ↓ for 20, 50 µM [Mn]	Li et al. (2007)
**Alveolata: Dinoflagellates**					
*Gonyaulax polyedra*	Cd, Cu, Hg, Pb	HgCl_2_, CdCl_2_, Pb(NO_3_)_2_, CuCl_2_ SOD activity was measured in algae exposed to sublethal (lower) or lethal concentrations: 5, 10 ppb [Hg], 0.1, 0.25 ppm [Cu], 0.5, 1 ppm [Cd], 2, 5 ppm [Pb], samples taken at t = 0, 1, 2, 6, 12, 24, 48, 96 h Dose dependent SOD changes were measured for 1–25 ppb [Hg]; 0.01–0.5 ppm [Cu], 0.5–5 ppm [Cd]; 0.5–10 ppm [Pb], exposure time 6 h Acute stress: 10 ppb [Hg], 0.25 ppm [Cu], 1 ppm [Cd], 5 ppm [Pb], exposure time 48 h Chronic stress: 5 ppb [Hg], 0.1 ppm [Cu], 0.5 ppm [Cd], 2 ppm [Pb], exposure time 30 d	TBARS: ↑	SOD changes in time, sublethal concentrations of heavy metals applied: rapid ↑ during the first 2 h, then slow ↑ until 48 h for Cu-exposed algae; ↑ for the first 12 h for Hg-, Cd-, Pb-treated algae, then slight ↓ in the case of Hg-treatment SOD changes in time, lethal concentrations of heavy metals applied: ↑ fast increase for 1 h then ↓ for Cu-treated algae; ↑ fast increase for 2 h then ↓ for Pb-treated algae; ↑ fast increase for 6 h then ↓ for Cd- and Hg-treated algae Dose dependent SOD activity: ↑, for concentrations higher than 10 ppb [Hg] there were no further dose-dependent increase, for other heavy metals applied the most pronounced increase was observed for 0.1 ppm [Cu], 2.5 ppm [Cd] and 5 ppm [Pb] SOD, acute stress: ↑ for Cd-, Cu-, Hg-treated algae; ↓ for Pb-treated algae SOD, chronic stress: ↑	Okamoto and Colepicolo (1998)
*Gonyaulax polyedra*	Cd, Cu, Hg, Pb	HgCl_2_, CdCl_2_, Pb(NO_3_)_2_, CuCl_2_ Acute stress: 10 ppb [Hg], 0.25 ppm [Cu], 1 ppm [Cd], 5 ppm [Pb], exposure time 48 h Chronic stress: 5 ppb [Hg], 0.1 ppm [Cu], 0.5 ppm [Cd], 2 ppm [Pb], exposure time 30 d	Lipid peroxidation (measured as conjugated dienes) and protein oxidation (measured as protein carbonyls): ↑ for all metals applied for acute stress treatment, in Pb-exposed algae for chronic stress	Acute stress: β-carotene: ↑ APX: ↑ in Hg- and Cd-exposed algae SOD: ↑ in Cd-exposed algae GSH/(GSH + 2GSSG) ratio: ↓ for all metals except Cu Chronic stress: β-carotene: = APX, SOD: ↑ GSH/(GSH + 2GSSG) ratio: ↑ in Hg-, Cd- and Cu-exposed algae	Okamoto et al. (2001a)
*Gonyaulax polyedra*	Cd, Cu, Hg, Pb	HgCl_2_ 0.04 µM CdCl_2_ 4.8 µM Pb(NO_3_)_2_ 18 µM CuCl_2_ 1.6 µM Exposure time 6 h		FeSOD (both protein content and activity): ↑	Okamoto et al. (2001b)
**Stramenopiles: diatoms**					
*Dityllum brightwellii*	Cu	CuSO_4_, in continuous culture [Cu] was increased stepwise starting from 3 nM to the final 126 nM at 121^st^ day, samples were taken at the end of the light photoperiod		SOD: ↑ GSH: ↓	Rijstenbil et al. (1994a)
*Phaeodactylum tricornutum*	Cu	Cu(NO_3_)_2_ 10 µM For enzyme activity determination, samples were taken at t = 0, 0.5, 1, 1.5, 2, 3, 4, 5, 6, 7, 24, 36, 48 h For TBARS determination, samples were taken at t = 0, 6, 24, 48 h	TBARS: ↑ in samples collected after 24, 48 h	SOD: ↑ for 24 h exposure and longer CAT: ↑ for 5 h exposure and longer APX, pyrogallol peroxidase: = GR: ↓ in the first 0.5 h of exposure; ↑ after 24 h and longer	Morelli and Scarano (2004)
*Odontella mobiliensis*	Cu	CuCl_2_ 21.5 ppb Exposure time 7 d	TBARS: ↑	CAT, POD: ↑ SOD: =	Manimaran et al. (2012)
*Chaetoceros calcitrans*	Cu	Cu (salt type not given): 50, 180, 450 µg/dm^3^ Samples were taken at t = 0, 24, 48, 72, 96, 120, 144, 168 h		SOD: ↑ for 180 µg/dm^3^ [Cu]; ↑ until 120 h then ↓ for 450 µg/dm^3^ [Cu] CAT: slight ↑ after 72 h for 50 µg/dm^3^ [Cu]; ↑ for 180 µg/dm^3^ [Cu]; ↑ till 96 h then ↓ for 450 µg/dm^3^ [Cu]	Anu et al. (2016)
*Amphora subtropica*	Ni	NiSO_4_ 0.64, 1.29, 1.93, 3.23 mM Exposure time: 7 d	TBARS: ↑	Car: ↑ SOD, CAT, GPX: ↑ then ↓ for 3.23 mM [Ni]	Moussa et al. (2018)
*Cyclotella* sp.	Cr	K_2_Cr_2_O_7_ 0.5, 1, 2, 5 mg/dm^3^ Exposure time 72 h	TBARS: ↑ for 1 mg/dm^3^ and higher [Cr]	SOD: ↑ for two lowest [Cr] CAT: ↑ for 1 mg/dm^3^ [Cr]	Li et al. (2021b)
**Stramenopiles: other**					
*Nannochloropsis oculata*	Cd	CdCl_2_ 10 µM For ROS determination, various photoperiods were applied (L:D 12:12, 16:8, 24:0); enzyme activities measured in algae grown at 12:12 L:D, exposure time 4 d	TBARS, H_2_O_2_: ↑, most pronounced for algae kept in continuous light	CAT, APX, guaiacol peroxidase: ↑ SOD, GR: ↓	Lee and Shin (2003)
**Excavata: Euglenids**					
*Euglena gracilis*	Cd	CdCl_2_ 50 µM Exposure time 4 d	TBARS: ↑	APX, GPX, GR, GSH + GSSG, UQ pool: ↑ GSH/(GSH + GSSG) ratio: ↓	Castro-Guerrero et al. (2008)
*Euglena gracilis,* Z-strain, G-strain	Cd, Hg, Pb	CdCl_2_, HgCl_2_, Pb(NO_3_)_2_ 50 ppm Algae were grown for 7 d, samples were taken at the time point of maximum accumulation of certain metal		Amount of GST: ↑ in Pb- and Cd-exposed Z-strain	Khatiwada et al. (2020)

The enhancement of the antioxidant response was often more pronounced in algae exposed to redox-active heavy metals than in algae treated with redox-inactive ones (Mehta and Gaur 1999; Nowicka et al. 2020). A similar effect was observed in higher plants (Collin et al. 2008). More pronounced formation of ROS in *C. reinhardtii* exposed to redox-active heavy metals (Cu, V), compared to redox-inactive ones (Zn, Cd) was reported by Stoiber et al. (2013). The exposure of red macroalga *G. tenuistipitata* to CuSO_4_ and CdCl_2_, applied in concentrations causing similar growth inhibition, resulted in more pronounced oxidative stress in the case of Cu treatment. What is more, SOD and APX activities were increased only in algae exposed to Cu^2+^ (Collén et al. 2003). Lipid peroxidation was more pronounced and the activity of H_2_O_2_-detoxifying enzymes was more enhanced in *C. vulgaris* exposed to Cu when compared to Cd- and Zn-treated algae (El-Naggar and Sheikh 2014). More pronounced pro-oxidant action of Cu when compared to Cd was also observed in *C. vulgaris* by Qian et al. (2011). Similar results were obtained for green microalgae belonging to the genus *Scenedesmus*, exposed to Cu and Cd or Cu and Zn (Tripathi et al. 2006; Štork et al. 2013). There was no increase in ^1^O_2_-specific products, PQ C and PC-OH in heavy metal-exposed *C. reinhardtii*, therefore it was concluded that the formation of this type of ROS was not increased in applied experimental conditions (Nowicka et al. 2016b, 2020). On the other hand, enhanced O_2_^•−^ generation was observed in stressed *C. reinhardtii*, and it was more pronounced in Cr-exposed than in Cd-exposed algae. The increase in the content of prenyllipid antioxidants, and hydrophilic ones Asc and Pro was higher in Cr-treated algae. In these algae, there was also an increase in SOD and CAT activity, which was not observed in Cd-exposed *C. reinhardtii* (Nowicka et al. 2020).

The sensitivity of given species to heavy metal applied is an important factor determining the response observed. However, this response is not a universal trend. *Chlorella kessleri* was shown to be more sensitive to Cu than *Scenedesmus vacuolatus*. Interestingly, the latter species accumulated more Cu ions and displayed higher MDA level. The antioxidant response of *S. vacuolatus* was more pronounced than that of *C. kessleri*, which was postulated to result in the increased tolerance of *S. vacuolatus* to Cu (Sabatini et al. 2009). *Scenedesmus acuminatus* turned out to be more tolerant to Cu than *Chlorella sorokiniana*, but in this case more tolerant alga accumulated less Cu and the activities of important antioxidant enzymes were either similar (APX, GR) or much lower (SOD) than in the less tolerant one (Hamed et al. 2017a). In Cu-exposed red algae *Gracilaria lemaneiformis* and *Gracilaria lichenoides*, more tolerant species *G. lichenoides* displayed lower intracellular Cu level and MDA content, and more effective antioxidant response (Huang et al. 2013).

The increase in the expression of genes encoding ROS-detoxifying enzymes (SOD, CAT, APX, GST, GPX, TRXs, putative PRX) and enzymes participating in the biosynthesis of low-molecular-weight antioxidants (*VTE3* needed for Toc and PQ synthesis, *P5CS* for Pro synthesis) was observed in heavy metal-treated *C. reinhardtii* (Lemaire et al. 1999; Jamers et al. 2006; Luis et al. 2006; Elbaz et al. 2010; Nowicka et al. 2016a). In *C. reinhardtii* exposed to 100 or 200 nM Ag^+^ for 1 h, the expression of genes encoding GST, GPX, MDHAR, DHAR, and TRX h1 was induced, whereas genes encoding SOD, CAT, APX and TRX x and f1 were downregulated (Pillai et al. 2014). The upregulation of the expression of APX, MDHAR and DHAR encoding genes, accompanied with the increase in the activity of their products was observed in Pb- or Hg-treated *Coccomyxa subellipsoidea* (Kováčik et al. 2017). An increase in the expression of genes encoding APX, CAT, and selected enzymes playing a role in GSH synthesis was observed in *C. sorokiniana* exposed to Cu, Cd and As (León-Vaz et al. 2021). Cd-treatment of the green macroalga *Ulva fasciata* resulted in an increase in the expression of genes encoding FeSOD and GR. The authors observed an increase in CAT and APX activity without changes in their expression levels what suggests that the posttranslational upregulation of enzyme activity is also important for the enhancement of antioxidant defence in response to heavy metal ions. Both the expression of *UfMnsod* and the activity of MnSOD remained unchanged in Cd-treated algae (Wu et al. 2009). On the other hand, the exposure of *U. fasciata* to Cu^2+^ resulted in an increase in the expression of all examined genes (*UfMnsod*, *UfFesod1*, *UfFesod2, Ufapx*, *Ufgr*, *Ufcat*), accompanied by an increase in the activities of their products. The only exception was MnSOD which activity remained unchanged in Cu-treated algae when compared to the control (Wu and Lee 2008). In *Ulva compressa*, the expression of genes encoding enzymes involved in antioxidant response (APX, PRX, TRX, GST) was induced in response to Cu during the first few days of exposure, but later it decreased. The expression of APX and TRX encoding genes reached its maximum earlier (day 3) than in the case of GST and PRX encoding genes (day 5). The increase in gene expression was accompanied with the increase in the activity of the above-mentioned enzymes (Contreras-Porcia et al. 2011). The expression of SOD encoding genes was upregulated in *C. ehrenbergii* exposed to Cu (applied as CuCl_2_ and CuSO_4_), Fe, Mn and Ni. The response of particular genes depended on heavy metal type, concentration of its salt, and time of exposure. Interestingly, significant differences were also observed between algae treated with various Cu salts (Wang and Ki 2020a, b). The expression of the gene encoding FeSOD in dinoflagellate *Gonyaulax polyedra* was upregulated in response to heavy metal ions tested (Hg^2+^, Cu^2+^, Cd^2+^, Pb^2+^) (Okamoto et al. 2001a). In another dinoflagellate species, *Prorocentrum minimum*, exposure to Cu^2+^ upregulated the expression of CAT encoding gene (Guo and Ki 2013). The SOD gene expression increased significantly in *E. gracilis* B-strain in response to Pb^2+^ and Hg^2+^ (Khatiwada et al. 2020). PRX gene was upregulated in Cu-treated brown alga *Scytosiphon gracilis*, while the genes encoding two FeSODs, GST, two GRXs and vanadium dependent bromoperoxidase were upregulated in Cu-treated brown alga *E. siliculosus* (Contreras et al. 2010; Ritter et al. 2014).

The activity of the rate-limiting enzyme of GSH biosynthetic pathway, γ-glutamylcysteine synthetase, was shown to increase in response to Cu-induced stress in green microalga *Scenedesmus bijugatus* (Nagalakshmi and Prasad 2001). Transgenic *C. reinhardtii* with overexpression of moth bean *P5CS* gene accumulated 80% more Pro and was more tolerant to Cd than the wild type. MDA content and GSH:GSSG ratio in Cd-exposed cells were lower in transgenic algae than in the wild type (Siripornadulsil et al. 2002). Exogenous Asc application alleviated Hg- and Pb-induced oxidative stress in green microalga *C. subellipsoidea* (Kováčik et al. 2017). Similarly, exogenously applied Pro partially protected against lipid peroxidation caused by Cu-, Cr-, Ni- or Zn-exposure (Mehta and Gaur 1999).

When stress is too severe, the decrease in antioxidants content occurs due to the fast oxidative degradation. In such a situation, the recycling and resynthesis mechanisms are not sufficient to maintain the stable level of these compounds. A decrease in α-Toc content in *C. reinhardtii* exposed to severe stress induced by application of Cu^2+^, Ag^+^, and Cr_2_O_7_^2−^ was accompanied by an increase in its oxidation product α-tocopheryl quinone. Such an increase was not observed in the case of Hg^2+^ and Cd^2+^ application, which did not cause α-Toc decrease (Nowicka et al. 2016b). In *U. fasciata,* the content of GSSG and DHA was similar to the control in algae exposed to lower CdCl_2_ concentrations (5 and 10 µM), whereas it was increased in algae treated with 20 and 50 µM CdCl_2_, suggesting enhanced GSH and Asc oxidation in the latter case (Wu et al. 2009). In heavy metal–exposed algae, GSH may be depleted as a result of induced synthesis of phytochelatins (Howe and Merchant 1992; Hu et al. 2001). Under stress conditions, the activity of antioxidant enzymes can be decreased due to the inhibitory action of heavy metal ions or ROS, i.e., Cd^2+^ inhibits Cu/ZnSOD, Cu^2+^, Zn^2+^ and Fe^3+^ inhibit GR (Nagalakshmi and Prasad 2001), H_2_O_2_ at higher concentration inhibits FeSOD, O_2_^•−^ inhibits heme-containing CAT. The ROS-induced degradation of antioxidant enzymes occurs under severe stress conditions.

The growth stage is also important for the observed response. In Cu-exposed *C. reinhardtii*, growth inhibition occurred during the exponential phase, and it was accompanied by an increase in MDA content. The activity of APX, SOD and CAT, and the content of PC-8 and total Asc were increased, while α-Toc and PQH_2_ levels were decreased in stressed algae. What is more, the big share of Asc pool was oxidized to DHA. The increased oxidative stress markers and the enhancement of antioxidant response were not observed at later stages of growth (Nowicka et al. 2021).

Low-molecular-weight antioxidants and antioxidant enzymes cooperate to detoxify ROS produced in heavy metal-exposed algae. Functional redundancy of various antioxidant systems has been observed. When *C. reinhardtii* was grown in the presence of toxic concentrations of heavy metal ions (Cu^2+^ and Cr_2_O_7_^2−^) and the inhibitor of the enzyme necessary for the synthesis of head-group precursor of PQ, Toc and PC-8, the culture growth rate did not differ from algae exposed to heavy metal ions in absence of the inhibitor. The decreased content of isoprenoid chromanols and PQ pool was compensated by the increased content of Asc and total soluble thiols, as well as the increased activity of SOD and APX (the latter only in Cd-exposed algae) (Nowicka et al. 2020).

The increase in antioxidant content, antioxidant enzyme activity, and the expression of related genes in response to heavy metal ions may be transient or it may be preceded by a decrease (Table 1). This should be remembered during the analysis of data collected at a single time point. Rijstenbil et al. (1994b) compared the response of two diatom species, *D. brightwellii*, and *Thalassiosira pseudonana* grown either in artificial medium or natural medium (sterile seawater) to Cd^2+^, Cu^2+^ and Zn^2+^. Among other parameters, they measured total nonprotein thiols, total GSH content, GSH:GSSG ratio, and SOD activity. Observed trends varied not only depending on a species, but also on the medium used (Rijstenbil et al. 1994b). Wang et al. (2018a) reported that the response of green algae *C. ehrenbergii* to Cu (photosynthetic pigment content, ROS formation, MDA level, SOD activity change) was not identical when CuCl_2_ and CuSO_4_ were applied.

The response to heavy metal ions also depends on the place from which certain algae were isolated. Sáez et al. (2015) investigated the antioxidant response to Cu-induced stress in three strains of brown alga *E. siliculosus*, of which two were isolated from Cu-contaminated sites (REP, Es524) and one from unpolluted waters (LIA). The increase in intracellular H_2_O_2_ was more pronounced in Cu-exposed LIA strain than in the other strains. The GSH:GSSG ratio decreased in LIA strain, while in two other strains it increased in response to Cu^2+^. Strains isolated from contaminated water had a higher basal level of SOD activity. There were also some differences between strains collected from polluted sites, for example, Es524 had higher basal CAT activity than two other strains and upregulated it in response to Cu^2+^, whereas in Cu-treated LIA and REP strains there was no increase in CAT activity (Sáez et al. 2015). The response to toxic concentrations of Cu^2+^ was also monitored in two species of brown algae, *Lessonia nigrescens* and the copper tolerant *Scytosiphon lomentaria*. More pronounced ROS formation and lipid peroxidation was observed in Cu-exposed *L. nigrescens*. On the other hand, the increase in the activity of antioxidant enzymes (CAT, APX, GPX, and DHAR) was faster and more pronounced in Cu-treated *S. lomentaria* (Contreras et al. 2009).

Gonzalez et al. (2010) showed in their experiments on Cu-treated *U. compressa* that Ca^2+^ release and ROS formation at the early stage of exposure regulate the differential activation of antioxidant enzymes. Further experiments in this model system showed that NO also plays a role in signalling in response to Cu^2+^-induced stress and that there is a cross-talk between Ca^2+^, H_2_O_2_ and NO (González et al. 2012). Exposure of *C. subellipsoidea* to Cd^2+^ resulted in NO formation. Exogenous application of NO donor modulated the response to Cd in these algae (Kováčik et al. 2015).

The participation of heme oxygenase and its product, CO, in the regulation of the response to heavy metal ions has been postulated (Wei et al. 2011). Transgenic *C. reinhardtii* overexpressing heme oxygenase-1 gene displayed improved tolerance to Hg-exposure, lower Hg accumulation, and less pronounced ROS-formation and lipid peroxidation when compared to Hg-treated wild type. A similar effect was achieved by exogenous CO application. APX and SOD activities in Hg-exposed algae pretreated with CO were not increased, whereas such an increase was observed in algae treated only with Hg^2+^ (Wei et al. 2011). *C. reinhardtii* pretreated with CO displayed increased tolerance to Cu^2+^ and alleviated oxidative stress symptoms in response to this heavy metal. SOD activity in algae exposed to CO and then to Cu^2+^ was similar to control, whereas lack of CO pretreatment led to the increase in SOD activity in response to Cu. On the other hand, the increase in APX activity in response to Cu was more pronounced in algae pretreated with CO when compared to *C. reinhardtii* exposed only to Cu^2+^ (Zheng et al. 2011).

Exogenous application of phytohormones belonging to auxins, cytokinins, gibberellin GA_3_, and jasmonic acid, as well as polyamine spermidine, modulated the response of *C. vulgaris* to Cd, Cu and Pb. Treatment with the regulatory compound usually resulted in a decrease in heavy metal-induced oxidative stress (H_2_O_2_ and MDA levels) and an increase in antioxidant response (carotenoids, Asc and GSH content, SOD, CAT and APX activity) when compared to algae exposed to heavy metal ions only. The reverse pattern of response was observed for jasmonic acid treatment, which led to the increase in oxidative stress and the decrease in antioxidant response in heavy metal-treated *C. vulgaris* (Piotrowska-Niczyporuk et al. 2012).

Knauert and Knauer (2008) monitored ROS formation in Cu-exposed *Pseudokirchneriella subcapitata* (*Selenastrum capricornutum*) and *C. vulgaris* in weak light (15 µmols photons/m^2^ s) or in darkness for 4.5 h. They observed an increase in ROS only under light conditions, which points to the role of photosynthesis disturbance in Cu-induced toxicity (Knauert and Knauer 2008). Exposure to both high light (HL) and toxic concentrations of heavy metal ions cause oxidative stress, therefore the research concerning the impact of the application of both of these stress factors has been carried out. Cheloni et al. (2014) observed that *C. reinhardtii* exposed to Cu^2+^ and HL accumulated more Cu in the cells, but displayed enhanced tolerance to Cu (monitored as growth rate, decrease in Chl fluorescence, ROS formation, lipid peroxidation) when compared to Cu-treated algae grown in low light. They also showed that HL-exposure upregulates the expression of genes encoding antioxidant enzymes. On the other hand, Cu^2+^ and UVB radiation acted synergistically (Cheloni et al. 2014). A synergistic effect was also observed for *C. reinhardtii* exposed to UV and Cd^2+^ (Korkaric et al. 2015). Nielsen and Nielsen (2010) reported that acclimation to HL enhanced tolerance of brown macroalga *Fucus serratus* to Cu^2+^.

It would be tempting to find a regular pattern of the antioxidant response of heavy metal-treated algae. However, the situation is much more complicated. Similar reviews containing a summary of the experiments carried out on plants confirmed the response of antioxidant defence to heavy metal-induced stress but did not show specific types of response for certain species or metal ions applied (Mourato et al. 2012; Sytar et al. 2013). In algae, a plethora of factors have an impact on the response: the concentration of heavy metal salt applied, sometimes even the type of heavy metal salt applied (chloride vs. sulphate), the species or strain used, the growth conditions (i.e., light intensity, photoperiod, medium type), the growth phase and the stage of the response. The cellular localization of certain protective mechanisms also has an impact, for example, FeSOD and MnSOD differed in their response to heavy metal-induced stress (Gillet et al. 2006; Wu and Lee 2008; Wu et al. 2009). The efficiency of other protective mechanisms, such as binding, bioprecipitation, sequestration and efflux have an impact on the actual concentration of heavy metal ions in sites, where they can disturb metabolism and enhance ROS generation. Therefore, the triggering of the enhancement of antioxidant response is a result of various other processes. The acclimation (increase in antioxidant defence) and exhaustion (decrease in defence) types of response seem to be a general trend, and the transition between the first and the second depends on the concentration of heavy metal applied vs sensitivity of certain species and the time of exposure.

## Other protective mechanisms

**Fig. 5:**
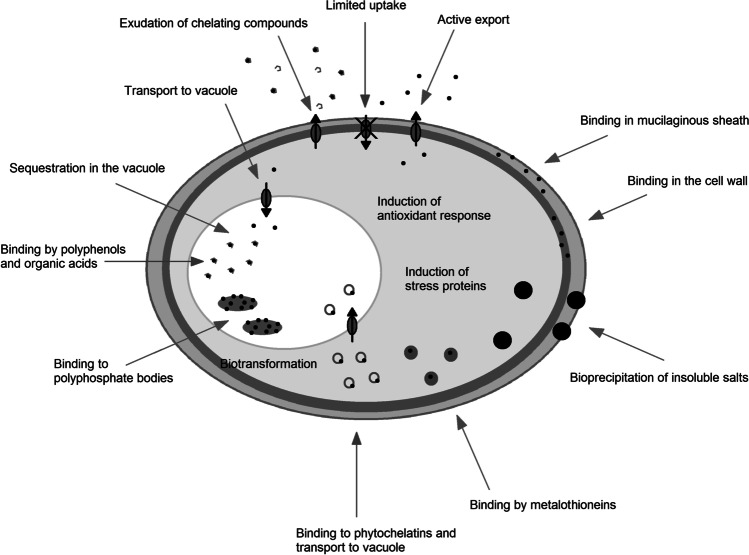
Major mechanisms of heavy metal detoxification in algae. In some species, heavy metal ions are sequestered not in the vacuole, but in plastids or mitochondria

Apart from antioxidant defence, living organisms have evolved several strategies to protect themselves from heavy metal toxicity (Fig. 5). Such metal detoxification strategies are binding of metal ions outside and inside of the cell, precipitation of insoluble metal complexes, reduced uptake, active export and bioconversion (Wood and Wang 1983; Gaur and Rai 2001). Mechanisms of active efflux and bioconversion have been extensively examined in prokaryotic organisms (Wood and Wang 1983; Nies 1999). These processes have been also reported to occur in some species of eukaryotic algae (Gaur and Rai 2001). Protective mechanism very important for both cyanobacteria and eukaryotic algae is based on the binding of metal ions to extracellular polymeric materials and cell walls. Many algal species secrete mucilaginous materials, usually polysaccharides able to efficiently bind metal ions. Sometimes even up to 80–90% of heavy metal ions accumulated by cells are bound on the cell surface. What is more, cell wall-deficient algal strains displayed decreased tolerance to heavy metals when compared to the wild type (Gaur and Rai 2001). Brown algae (*Phaeophyta*) are considered to be efficient heavy metal accumulators due to high levels of alginates and sulphated polysaccharides in their cell walls (Chekroun and Baghour 2013). These algae are also known to exude polyphenol compounds able to bind heavy metal ions. Some of these polyphenols serve as metal chelators also in cell walls and inside algal cells (Connan and Stengel 2011; Zolotareva et al. 2019).

Synthesis of peptides capable to bind metal ions is considered to be a preferential way of heavy metal detoxification inside the cell. Organometallic complexes are further partitioned inside vacuoles (Perales-Vela et al. 2006). Metal-binding peptides can be subdivided into two major groups: (1) phytochelatins, which are short-chain polypeptides synthesized enzymatically, (2) metallothioneins, which are gene-encoded and synthesized on ribosomes. Both types occur in algae (Perales-Vela et al. 2006; Balzano et al. 2020). Metallothioneins are small (less than 300 aa), cytosolic proteins containing a high proportion of Cys (15–35%) (Balzano et al. 2020). Phytochelatin structure can be written as (γ-Glu-Cys)_n_-Gly, where n is 2–11. Sometimes, other amino acids, such as Ala, Ser, Glu or Gln, occur in phytochelatins instead of Gly. Cd^2+^ ions are the most potent activator of phytochelatin synthesis, but other heavy metals, such as Ag^+^, Pb^2+^, Zn^2+^, Cu^2+^, Hg^2+^, Ni^2+^, Co^2+^, and Bi^3+^ are also known to induce it. Phytochelatins were also observed to be synthesized in response to metalloids such as As (Perales-Vela et al. 2006). However, in vivo studies confirmed the occurrence of phytochelatin complexes mainly for Cd^2+^ and Cu^2+^ (Gaur and Rai 2001). Phytochelatins have been discovered in various algal phyla: green and red algae, dinoflagellates, and clades belonging to heterokonts (Gaur and Rai 2001). In *C. reinhardtii,* about 70% of Cd^2+^ present in cells is bound to phytochelatins (Howe and Merchant 1992). Complexes of phytochelatins with heavy metal ions were observed in vacuoles of green algae and diatoms. On the other hand, *E. gracilis*, belonging to a clade unrelated to the above-mentioned algae, does not have plant-like vacuole serving as a reservoir organelle. In this species, complexes of heavy metals with thiol compounds are stored in chloroplasts and mitochondria (Perales-Vela et al. 2006). Chloroplastic metal storage was also observed for the green algae *Oocystis nephrocytioides* (Soldo et al. 2005). The chelation of heavy metal ions by GSH or tripeptide Arg-Arg-Glu was described for certain algal species (Perales-Vela et al. 2006). Under phosphate surplus conditions, many algae are able to sequester metal cations in polyphosphate bodies. Accumulation of heavy metal ions in polyphosphate bodies was observed in species belonging to diatoms and green algae (Gaur and Rai 2001). Higher plants are known to chelate metal ions with organic acids. Such complexes were also observed in vacuoles of Cd-treated *C. reinhardtii* (Penen et al. 2017). In the cytosol of some algae, insoluble metal salts have been discovered (Perales-Vela et al. 2006). The role of free His in preventing Ni-induced toxicity in *C. reinhardtii* was reported by Zheng et al. (2013).

These various mechanisms together counteract the toxic action of heavy metal ions. Algal species displaying relatively high tolerance to these pollutants and the ability of efficient metal-binding may be very useful in phytoremediation.

Early research on the response to heavy metal-induced stress usually was aimed to assess the role of selected mechanisms. Later, a more holistic approach was applied. Varied “omics” techniques have been used to analyse algal response. Proteomic, transcriptomic and metabolomic analyses were carried out for Cd-exposed *C. reinhardtii* (Gillet et al. 2006; Jamers et al. 2013), another example is microarray study of Cu-exposed *C. reinhardtii* (Jamers et al. 2006). Recently, such an approach has become popular, as it gives wider insight into processes occurring in stress-exposed organisms. The soluble protein profile was analysed in Cu, Ni, Cd, Pb and Zn-exposed dinoflagellate *A. pacificum* (Jean et al. 2021; Wang et al. 2021a). Proteomic analysis was performed for brown macroalga *Macrocystis pyrifera* exposed to Cr (Wang et al. 2021b). Metabolomic and transcriptomic profiling were recently carried out for Cd-exposed *Dunalliela salina* (Zhu et al. 2021). The analysis of gene transcription and lipid profile in Cd-exposed *A. protothecoides* was carried out to provide insight into the role of GSH in the regulation of triacylglycerol synthesis in stressed algae (Xing et al. 2021). An extensive review summarizing the results of transcriptomic analyses of heavy metal detoxification in microalgae has been recently published (Tripathi and Poluri 2021). The metabolome changes were assessed in *C. reinhardtii* exposed to inorganic HgCl_2_ and methyl-Hg (Slaveykova et al. 2021). Lipidomic analysis was carried out for Pb-treated *C. sorokiniana* (Nanda et al. 2021).

## Conclusions and future perspectives

The research on the antioxidant response in heavy metal-exposed algae has not been as extensive as in the case of higher plants, but a substantial amount of data has been collected during the last 30 years. The role of low-molecular-weight antioxidants and ROS-detoxifying enzymes in the acclimation to heavy metal-induced stress has been proved.

An important research direction takes advantage of “omics” techniques and aims at obtaining an integrated picture of the response to heavy metal-induced stress. Now, when we know which processes are induced in stressed algae, it is crucial to understand their interrelations and the signalling pathways responsible for the stress perception and the induction of protective mechanisms.

Green algae are the clade most researched for their response to heavy metals, whereas the experiments carried out on other algal groups are less numerous. Therefore, it is important to expand our knowledge concerning the toxicity and tolerance to heavy metals in other important systematic groups, such as diatoms, dinoflagellates and haptophytes.

Another valuable approach concerns the assessment of the impact of combined stress factors, such as mixtures of heavy metals, heavy metals + xenobiotics, heavy metals + light. Such experiments better reflect the situation in nature. Considering experiments in which the conditions are more similar to those occurring in the environment, the usage of low concentrations of heavy metal salts is also important (Expósito et al. 2021).

Algae have great potential for application in wastewater treatment, therefore further research on their ability for biosorption and bioremediation is crucial. A better understanding of mechanisms of heavy metal toxicity and tolerance will enable the selection of the strains most suitable for removing these pollutants. The transgenic approach is also promising, because it makes it possible to manipulate traits to provide enhanced tolerance to heavy metals.

## Data Availability

Not applicable.

## Abbreviations

APX: Ascorbate peroxidase
Asc: Ascorbate
CAT: Catalase
Chl: Chlorophyll
DHA: Dehydroascorbate
DHAR: Dehydroascorbate reductase
GPX: Glutathione peroxidase
GR: Glutathione reductase
GRX: Glutaredoxin
GSH: Glutathione
GSSG: Glutathione disulphide
GST: Glutathione-S-transferase
HL: High light
HSP: Heat shock protein
MDA: Malonyldialdehyde
MDHA: Monodehydroascorbate
MDHAR: Monodehydroascorbate reductase
NO: Nitric oxide
PC-8: Plastochromanol-8
PQ: Plastoquinone
PQH_2_: Plastoquinol
PRX: Peroxiredoxin
Pro: Proline
PS I: Photosystem I
PS II: Photosystem II
PUFA: Polyunsaturated fatty acid
R^•^: Carbon-centered radical
RO^•^: Alkoxy radical
ROO^•^: Peroxy radical
ROOH: Organic hydroperoxide
ROS: Reactive oxygen species
SOD: Superoxide dismutase
T_3_: Tocotrienol
Toc: Tocopherol
α-Toc: α-Tocopherol
TRX: Thioredoxin

## References

[CR1] Abassi S, Wang H, Ponmani T, Ki JS (2019). Small heat shock protein genes of the green algae *Closterium ehrenbergii*: cloning and differential expression under heat and heavy metal stresses. Environ Toxicol.

[CR2] Ackova DG (2018). Heavy metals and their general toxicity on plants. Plant Sci Today.

[CR3] Agati G, Azzarello E, Pollastri S, Tattini M (2012). Flavonoids as antioxidants in plants: Location and functional significance. Plant Sci.

[CR4] Ahmad P, Jaleel CA, Salem MA (2010). Roles of enzymatic and nonenzymatic antioxidants in plants during abiotic stress. Crit Rev Biotechnol.

[CR5] Ajitha V, Sreevidya CP, Sarasan M (2021). Effects of zinc and mercury on ROS-mediated oxidative stress-induced physiological impairments and antioxidant responses in the microalga *Chlorella vulgaris*. Environ Sci Pollut Res.

[CR6] Alscher RG, Erturk N, Heath LS (2002). Role of superoxide dismutases (SODs) in controlling oxidative stress in plants. J Exp Bot.

[CR7] Anjum NA, Ahmad I, Mohmood I (2012). Modulation of glutathione and its related enzymes in plants’ responses to toxic metals and metalloids-A review. Environ Exp Bot.

[CR8] Anjum NA, Sharma P, Gill SS (2016). Catalase and ascorbate peroxidase - representative H_2_O_2_-detoxifying heme enzymes in plants. Environ Sci Pollut Res.

[CR9] Antia NJ, Desai ID, Romilly MJ (1970). The tocopherol, vitamin K, and related isoprenoid quinone composition of a unicellular red alga (*Porphyridium cruentum*). J Phycol.

[CR10] Anu PR, Bijoy Nandan S, Jayachandran PR, Don Xavier ND (2016). Toxicity effects of copper on the marine diatom, *Chaetoceros calcitrans*. Reg Stud Mar Sci.

[CR11] Arias DG, Marquez VE, Beccaria AJ (2010). Purification and characterization of a glutathione reductase from *Phaeodactylum tricornutum*. Protist.

[CR12] Arun N, Vidyalaxmi, Singh DP (2014). Chromium (VI) induced oxidative stress in halotolerant alga Dunaliella salina and D. tertiolecta isolated from sambhar salt lake of Rajasthan (India). Cell Mol Biol.

[CR13] Arunakumara K, Zhang X (2008). Heavy metal bioaccumulation and toxicity with special reference to microalgae. J Ocean Univ China.

[CR14] Asada K, Kanematsu S, Uchida K (1977). Superoxide dismutases in photosynthetic organisms: absence of the cuprozinc enzyme in eukaryotic algae. Arch Biochem Biophys.

[CR15] Azevedo R, Rodriguez E (2012). Phytotoxicity of mercury in plants: a review. J Bot.

[CR16] Balasaraswathi K, Jayaveni S, Sridevi J (2017). Cr–induced cellular injury and necrosis in *Glycine max* L.: biochemical mechanism of oxidative damage in chloroplast. Plant Physiol Biochem.

[CR17] Balzano S, Sardo A, Blasio M (2020). Microalgal metallothioneins and phytochelatins and their potential use in bioremediation. Front Microbiol.

[CR18] Banerjee A, Roychoudhury A, Hasanuzzaman M, Fotopoulos V, Nahar K, Fujita M (2019). Role of glutathione in plant abiotic stress tolerance. Reactive oxygen, nitrogen and sulfur species in plants: production, metabolism, signaling and defense mechanisms.

[CR19] Barondeau DP, Kassmann CJ, Bruns CK (2004). Nickel superoxide dismutase structure and mechanism. Biochemistry.

[CR20] Barrett J, Girr P, Mackinder LCM (2021). Pyrenoids: CO_2_-fixing phase separated liquid organelles. Biochim Biophys Acta – Mol Cell Res.

[CR21] Benavides MP, Gallego SM, Tomaro ML (2005). Cadmium toxicity in plants. Brazillian J Plant Physiol.

[CR22] Bilodeau C, Chevrier N (1998). Endogenous ascorbate level modulates ozone tolerance in *Euglena gracilis* cells. Plant Physiol Biochem.

[CR23] Boening DW (2000). Ecological effects, transport, and fate of mercury: a general review. Chemosphere.

[CR24] Broadley MR, White PJ, Hammond JP (2007). Zinc in plants: Tansley review. New Phytol.

[CR25] Brown MR, Miller KA (1992). The ascorbic acid content of eleven species of microalgae used in mariculture. J Appl Phycol.

[CR26] Burda K, Kruk J, Schmid GH, Strzalka K (2003). Inhibition of oxygen evolution in Photosystem II by Cu(II) ions is associated with oxidation of cytochrome *b559*. Biochem J.

[CR27] Castro-Guerrero NA, Rodríguez-Zavala JS, Marín-Hernández A (2008). Enhanced alternative oxidase and antioxidant enzymes under Cd^2+^ stress in *Euglena*. J Bioenerg Biomembr.

[CR28] Caverzan A, Passaia G, Barcellos Rosa S (2012). Plant responses to stresses: role of ascorbate peroxidase in the antioxidant protection. Genet Mol Biol.

[CR29] Cervantes C, Campos-García J, Devars S (2001). Interactions of chromium with microorganisms and plants. FEMS Microbiol Rev.

[CR30] Chang YC, Lee TM (1999). High temperature-induced free proline accumulation in *Gracilaria tenuistipitata* (Rhodophyta). Bot Bull Acad Sin.

[CR31] Chekroun KB, Baghour M (2013). The role of algae in phytoremediation of heavy metals: a review. J Mater Environ Sci.

[CR32] Cheloni G, Cosio C, Slaveykova VI (2014). Antagonistic and synergistic effects of light irradiation on the effects of copper on *Chlamydomonas reinhardtii*. Aquat Toxicol.

[CR33] Chen J, Yang ZM (2012). Mercury toxicity, molecular response and tolerance in higher plants. Biometals.

[CR34] Chen Z, Song S, Wen Y (2016). Toxicity of Cu (II) to the green alga *Chlorella vulgaris*: a perspective of photosynthesis and oxidant stress. Environ Sci Pollut Res.

[CR35] Cheng J, Qiu H, Chang Z (2016). The effect of cadmium on the growth and antioxidant response for freshwater algae *Chlorella vulgaris*. Springerplus.

[CR36] Collén J, Pinto E, Pedersén M, Colepicolo P (2003). Induction of oxidative stress in the red macroalga *Gracilaria tenuistipitata* by pollutant metals. Arch Environ Contam Toxicol.

[CR37] Collin VC, Eymery F, Genty B (2008). Vitamin E is essential for the tolerance of *Arabidopsis thaliana* to metal-induced oxidative stress. Plant, Cell Environ.

[CR38] Colmano G (1973). Molybdenum toxicity: Abnormal cellular division of teratogenic appearance in *Euglena gracilis*. Bull Environ Contam Toxicol.

[CR39] Connan S, Stengel DB (2011). Impacts of ambient salinity and copper on brown algae: 2. Interactive effects on phenolic pool and assessment of metal binding capacity of phlorotannin. Aquat Toxicol.

[CR40] Contreras-Porcia L, Dennett G, Gonzalez A (2011). Identification of copper-induced genes in the marine alga *Ulva compressa* (Chlorophyta). Mar Biotechnol.

[CR41] Contreras L, Mella D, Moenne A, Correa JA (2009). Differential responses to copper-induced oxidative stress in the marine macroalgae *Lessonia nigrescens* and *Scytosiphon lomentaria* (Phaeophyceae). Aquat Toxicol.

[CR42] Contreras L, Moenne A, Gaillard F (2010). Proteomic analysis and identification of copper stress-regulated proteins in the marine alga *Scytosiphon gracilis* (Phaeophyceae). Aquat Toxicol.

[CR43] Coulombier N, Jauffrais T, Lebouvier N (2021). Antioxidant compounds from microalgae: A review. Mar Drugs.

[CR44] Couturier J, Jacquot JP, Rouhier N (2009). Evolution and diversity of glutaredoxins in photosynthetic organisms. Cell Mol Life Sci.

[CR45] Cruces E, Rautenberger R, Rojas-Lillo Y (2017). Physiological acclimation of *Lessonia spicata* to diurnal changing PAR and UV radiation: differential regulation among down-regulation of photochemistry, ROS scavenging activity and phlorotannins as major photoprotective mechanisms. Photosynth Res.

[CR46] DalCorso G, Furini A (2012). Heavy metal toxicity in plants. Plants and heavy metals.

[CR47] Danouche M, El Ghachtouli N, El Arroussi H (2021). Phycoremediation mechanisms of heavy metals using living green microalgae: physicochemical and molecular approaches for enhancing selectivity and removal capacity. Heliyon.

[CR48] Danouche M, El Ghachtouli N, El Baouchi A, El Arroussi H (2020). Heavy metals phycoremediation using tolerant green microalgae: enzymatic and non-enzymatic antioxidant systems for the management of oxidative stress. J Environ Chem Eng.

[CR49] Das K, Roychoudhury A (2014). Reactive oxygen species (ROS) and response of antioxidants as ROS-scavengers during environmental stress in plants. Front Environ Sci.

[CR50] Dayer R, Fischer BB, Eggen RIL, Lemaire SD (2008). The peroxiredoxin and glutathione peroxidase families in *Chlamydomonas reinhardtii*. Genetics.

[CR51] Del Río LA, Sandalio LM, Corpas FJ (2006). Reactive oxygen species and reactive nitrogen species in peroxisomes. Production, scavenging, and role in cell signaling. Plant Physiol.

[CR52] Dewez D, Geoffroy L, Vernet G, Popovic R (2005). Determination of photosynthetic and enzymatic biomarkers sensitivity used to evaluate toxic effects of copper and fludioxonil in alga *Scenedesmus obliquus*. Aquat Toxicol.

[CR53] Díaz S, de Francisco P, Olsson S (2020). Toxicity, physiological, and ultrastructural effects of arsenic and cadmium on the extremophilic microalga *Chlamydomonas acidophila*. Int J Environ Res Public Health.

[CR54] Dietz KJ (2005) Plant thiol enzymes and thiol homeostasis in relation to thiol-dependent redox regulation and oxidative stress. In: Smirnoff N (ed) Antioxidants and reactive oxygen species in plants. Blackwell Publishing Ltd, pp 25–52

[CR55] Dietz KJ (2011). Peroxiredoxins in plants and cyanobacteria. Antioxidants Redox Signal.

[CR56] Dittami SM, Gravot A, Renault D (2011). Integrative analysis of metabolite and transcript abundance during the short-term response to saline and oxidative stress in the brown alga *Ectocarpus siliculosus*. Plant, Cell Environ.

[CR57] Dos Santos CV, Rey P (2006). Plant thioredoxins are key actors in the oxidative stress response. Trends Plant Sci.

[CR58] Edreva A (2005). Generation and scavenging of reactive oxygen species in chloroplasts: a submolecular approach. Agric Ecosyst Environ.

[CR59] El-Jaoual T, Cox DA (1998). Manganese toxicity in plants. J Plant Nutr.

[CR60] El-Naggar AH, Sheikh HM (2014). Response of the green microalga *Chlorella vulgaris* to the oxidative stress caused by some heavy metals. Life Sci J.

[CR61] Elbaz A, Wei YY, Meng Q (2010). Mercury-induced oxidative stress and impact on antioxidant enzymes in *Chlamydomonas reinhardtii*. Ecotoxicology.

[CR62] Evans LJ, Barabash SJ (2010) Molybdenum, silver, thallium and vanadium. In: Hooda PS (ed) Trace elements in soils. Wiley, pp 516–549

[CR63] Expósito N, Carafa R, Kumar V (2021). Performance of *Chlorella vulgaris* exposed to heavy metal mixtures: Linking measured endpoints and mechanisms. Int J Environ Res Public Health.

[CR64] Fageria NK, Santos AB, Barbosa Filho MP, Guimarães CM (2008). Iron toxicity in lowland rice. J Plant Nutr.

[CR65] Farooq MA, Islam F, Ali B (2016). Arsenic toxicity in plants: cellular and molecular mechanisms of its transport and metabolism. Environ Exp Bot.

[CR66] Feierabend J (2005) Catalases in plants: molecular and functional properties and role in stress defence. In: Smirnoff N (ed) Antioxidants and reactive oxygen species in plants. Blackwell Publishing Ltd, pp 101–140

[CR67] Fernandes JC, Henriques FS (1991). Biochemical, physiological, and structural effects of excess copper in plants. Bot Rev.

[CR68] Fernandez-Panchon MS, Villano D, Troncoso AM, Garcia-Parrilla MC (2008). Antioxidant activity of phenolic compounds: from in vitro results to in vivo evidence. Crit Rev Food Sci Nutr.

[CR69] Filová A, Fargašová A, Molnárová M (2021). Cu, Ni, and Zn effects on basic physiological and stress parameters of *Raphidocelis subcapitata* algae. Environ Sci Pollut Res.

[CR70] Flemming CA, Trevors JT (1989). Copper toxicity and chemistry in the environment: a review. Water Air Soil Pollut.

[CR71] Foo SC, Yusoff FM, Ismail M (2017). Antioxidant capacities of fucoxanthin-producing algae as influenced by their carotenoid and phenolic contents. J Biotechnol.

[CR72] Foyer CH, Gomez LD, van Heerden PDR (2005) Glutathione. In: Smirnoff N (ed) Antioxidants and reactive oxygen species in plants. Blackwell Publishing Ltd, pp 1–24

[CR73] Foyer CH, Noctor G (2005). Oxidant and antioxidant signalling in plants: a re-evaluation of the concept of oxidative stress in a physiological context. Plant Cell Environ.

[CR74] Fu HY, Liu SL, Chiang YR (2020). Biosynthesis of ascorbic acid as a glucose-induced photoprotective process in the extremophilic red alga *Galdieria partita*. Front Microbiol.

[CR75] García-Caparrós P, De Filippis L, Gul A, et al (2020) Oxidative stress and antioxidant metabolism under adverse environmental conditions: a review. Bot Rev 1–4610.1007/s12229-020-09231-1

[CR76] Gaur JP, Rai LC, Gaur JP, Rai LC (2001). Heavy metal tolerance in algae. Algal adaptation to environmental stresses.

[CR77] Gechev TS, Van Breusegem F, Stone JM (2006). Reactive oxygen species as signals that modulate plant stress responses and programmed cell death. BioEssays.

[CR78] Geigenberger P, Thormählen I, Daloso DM, Fernie AR (2017). The unprecedented versatility of the plant thioredoxin system. Trends Plant Sci.

[CR79] Geng A, Wang X, Wu L (2017). Arsenic accumulation and speciation in rice grown in arsanilic acid-elevated paddy soil. Ecotoxicol Environ Saf.

[CR80] Geng W, Xiao X, Zhang L, et al (2021) Response and tolerance ability of *Chlorella vulgaris* to cadmium pollution stress. Environ Technol (United Kingdom) 1–1110.1080/09593330.2021.1950841 10.1080/09593330.2021.1950841 34278946

[CR81] Gest N, Gautier H, Stevens R (2013). Ascorbate as seen through plant evolution: the rise of a successful molecule?. J Exp Bot.

[CR82] Gill SS, Anjum NA, Hasanuzzaman M (2013). Glutathione and glutathione reductase: a boon in disguise for plant abiotic stress defense operations. Plant Physiol Biochem.

[CR83] Gillet S, Decottignies P, Chardonnet S, Le Maréchal P (2006). Cadmium response and redoxin targets in *Chlamydomonas reinhardtii*: a proteomic approach. Photosynth Res.

[CR84] González A, de los Ángeles Cabrera M, Josefa Henríquez M (2012). Cross talk among calcium, hydrogen peroxide, and nitric oxide and activation of gene expression involving calmodulins and calcium-dependent protein kinases in *Ulva compressa* exposed to copper excess. Plant Physiol.

[CR85] Gonzalez A, Vera J, Castro J (2010). Co-occurring increases of calcium and organellar reactive oxygen species determine differential activation of antioxidant and defense enzymes in *Ulva compressa* (Chlorophyta) exposed to copper excess. Plant, Cell Environ.

[CR86] Goswami RK, Agrawal K, Shah MP, Verma P (2021) Bioremediation of heavy metals from wastewater: a current perspective on microalgae-based future. Lett. Appl. Microbiol. 1–17 10.1111/lam.13564 34562022

[CR87] Gouveia C, Kreusch M, Schmidt EC (2013). The effects of lead and copper on the cellular architecture and metabolism of the red alga *Gracilaria domingensis*. Microsc Microanal.

[CR88] Grace SC (1990). Phylogenetic distribution of superoxide dismutase supports an endosymbiotic origin for chloroplasts and mitochondria. Life Sci.

[CR89] Gruszka J, Pawlak A, Kruk J (2008). Tocochromanols, plastoquinol, and other biological prenyllipids as singlet oxygen quenchers-determination of singlet oxygen quenching rate constants and oxidation products. Free Radic Biol Med.

[CR90] Guo R, Ki JS (2013). Characterization of a novel catalase-peroxidase (KATG) gene from the dinoflagellate *Prorocentrum minimum*. J Phycol.

[CR91] Habibi G, Ahmad P (2014). Hydrogen peroxide (H_2_O_2_) generation, scavenging and signaling in plants. Oxidative damage to plants: antioxidant networks and signaling.

[CR92] Hajiboland R, Ahmad P (2014). Reactive oxygen species and photosynthesis. Oxidative damage to plants: antioxidant networks and signaling.

[CR93] Halliwell B (2006). Reactive species and antioxidants. Redox biology is a fundamental theme of aerobic life. Plant Physiol.

[CR94] Hamed SM, Selim S, Klöck G, AbdElgawad H (2017). Sensitivity of two green microalgae to copper stress: growth, oxidative and antioxidants analyses. Ecotoxicol Environ Saf.

[CR95] Hamed SM, Zinta G, Klöck G (2017). Zinc-induced differential oxidative stress and antioxidant responses in *Chlorella sorokiniana* and *Scenedesmus acuminatus*. Ecotoxicol Environ Saf.

[CR96] Hansel CM, Diaz JM (2021). Production of extracellular reactive oxygen species by marine biota. Ann Rev Mar Sci.

[CR97] Harris ZL, Gitlin JD (1996). Genetic and molecular basis for copper toxicity. Am J Clin Nutr.

[CR98] Hasan MK, Cheng Y, Kanwar MK (2017). Responses of plant proteins to heavy metal stress - a review. Front Plant Sci.

[CR99] He S, Yang X, He Z, Baligar VC (2017). Morphological and physiological responses of plants to cadmium toxicity: a review. Pedosphere.

[CR100] Hossain MA, Hoque MA, Burritt DJ, Fujita M, Ahmad P (2014). Proline protects plants against abiotic oxidative stress: biochemical and molecular mechanisms. Oxidative damage to plants: Antioxidant networks and signaling.

[CR101] Howe G, Merchant S (1992). Heavy metal-activated synthesis of peptides in *Chlamydomonas reinhardtii*. Plant Physiol.

[CR102] Hu S, Lau KWK, Wu M (2001). Cadmium sequestration in *Chlamydomonas reinhardtii*. Plant Sci.

[CR103] Huang H, Liang J, Wu X (2013). Comparison in copper accumulation and physiological responses of *Gracilaria lemaneiformis* and *G. lichenoides* (Rhodophyceae). Chinese J Oceanol Limnol.

[CR104] Imahori Y (2014) Role of ascorbate peroxidase in postharvest treatments of horticultural crops. In: Ahmad P (ed) Oxidative damage to plants: antioxidant networks and signaling. Academic Press, pp 425–451

[CR105] Imtiaz M, Rizwan MS, Xiong S (2015). Vanadium, recent advancements and research prospects: a review. Environ Int.

[CR106] Ishikawa T, Shigeoka S (2008). Recent advances in ascorbate biosynthesis and the physiological significance of ascorbate peroxidase in photosynthesizing organisms. Biosci Biotechnol Biochem.

[CR107] Ishikawa T, Tamaki S, Maruta T, Shigeoka S, Schwartzbach SD, Shigeoka S (2017). Biochemistry and physiology of reactive oxygen species in *Euglena*. Advances in Experimental Medicine and Biology.

[CR108] Ismael MA, Elyamine AM, Moussa MG (2019). Cadmium in plants: uptake, toxicity, and its interactions with selenium fertilizers. Metallomics.

[CR109] Jamers A, Blust R, De Coen W (2013). An omics based assessment of cadmium toxicity in the green alga *Chlamydomonas reinhardtii*. Aquat Toxicol.

[CR110] Jamers A, Van der Ven K, Moens L (2006). Effect of copper exposure on gene expression profiles in *Chlamydomonas reinhardtii* based on microarray analysis. Aquat Toxicol.

[CR111] James AM, Smith RAJ, Murphy MP (2004). Antioxidant and prooxidant properties of mitochondrial Coenzyme Q. Arch Biochem Biophys.

[CR112] Jayasree V, Solimabi, Kamat SY (1985). Distribution of tocopherol (vitamin E) in marine algae from Goa, West coast of India. Indian J Geo-Marine Sci.

[CR113] Jean N, Perié L, Dumont E, et al (2021) Metal stresses modify soluble proteomes and toxin profiles in two Mediterranean strains of the distributed dinoflagellate *Alexandrium pacificum*. Sci Total Environ 151680 10.1016/j.scitotenv.2021.151680 34793790

[CR114] Jervis L, Rees-Naesborg R, Brown M (1997). Biochemical responses of the marine macroalgae *Ulva lactuca* and *Fucus vesiculosus* to cadmium and copper - from sequestration to oxidative stress. Biochem Soc Trans.

[CR115] Jiang Y, Zhu Y, Hu Z (2016). Towards elucidation of the toxic mechanism of copper on the model green alga *Chlamydomonas reinhardtii*. Ecotoxicology.

[CR116] Kalaivanan D, Ganeshamurthy AN, Srinivasa Rao NK, Shivashankara KS, Laxman RH (2016). Mechanisms of heavy metal toxicity in plants. Abiotic stress physiology of horticultural crops.

[CR117] Kanematsu S, Asada K (1979). Ferric and manganic superoxide dismutases in *Euglena gracilis*. Arch Biochem Biophys.

[CR118] Kato J, Yamahara T, Tanaka K (1997). Characterization of catalase from green algae *Chlamydomonas reinhardtii*. J Plant Physiol.

[CR119] Kato N, Nelson G, Lauersen KJ (2021). Subcellular localizations of catalase and exogenously added fatty acid in *Chlamydomonas reinhardtii*. Cells.

[CR120] Kaul S, Sharma SS, Mehta IK (2008). Free radical scavenging potential of L-proline: evidence from in vitro assays. Amino Acids.

[CR121] Kaur G (2014). Pb-induced toxicity in plants: effect on growth, development, and biochemical attributes. J Glob Biosci.

[CR122] Kaur R, Nayyar H (2014) Ascorbic acid: a potent defender against environmental stresses. In: Ahmad P (ed) Oxidative damage to plants: antioxidant networks and signaling. Academic Press, pp 235–287

[CR123] Keeling PJ (2004). Diversity and evolutionary history of plastids and their hosts. Am J Bot.

[CR124] Khan T, Mazid M, Mohammad F (2011). A review of ascorbic acid potentialities against oxidative stress induced in plants. J Agrobiol.

[CR125] Khatiwada B, Hasan MT, Sun A (2020). Proteomic response of *Euglena gracilis* to heavy metal exposure – identification of key proteins involved in heavy metal tolerance and accumulation. Algal Res.

[CR126] Knauert S, Knauer K (2008). The role of reactive oxygen species in copper toxicity to two freshwater green algae. J Phycol.

[CR127] Kohen R, Nyska A (2002). Oxidation of biological systems: oxidative stress phenomena, antioxidants, redox reactions, and methods for their quantification. Toxicol Pathol.

[CR128] Korkaric M, Behra R, Fischer BB (2015). Multiple stressor effects in *Chlamydomonas reinhardtii* - toward understanding mechanisms of interaction between effects of ultraviolet radiation and chemical pollutants. Aquat Toxicol.

[CR129] Kováčik J, Klejdus B, Babula P, Hedbavny J (2015). Nitric oxide donor modulates cadmium-induced physiological and metabolic changes in the green alga *Coccomyxa subellipsoidea*. Algal Res.

[CR130] Kováčik J, Rotková G, Bujdoš M (2017). Ascorbic acid protects *Coccomyxa subellipsoidea* against metal toxicity through modulation of ROS/NO balance and metal uptake. J Hazard Mater.

[CR131] Kowalczyk N, Rappaport F, Boyen C (2013). Photosynthesis in *Chondrus crispus*: The contribution of energy spill-over in the regulation of excitonic flux. Biochim Biophys Acta - Bioenerg.

[CR132] Kozak A, Unal D, Tuney-Kizilkaya I (2020). Seasonal variations of epiphytic flora, abscisic acid production and physiological response in the brown alga *Cystoseira foeniculacea* (Linnaeus) Greville. Cah Biol Mar.

[CR133] Krieger-Liszkay A (2005). Singlet oxygen production in photosynthesis. J Exp Bot.

[CR134] Kruk J, Szymańska R, Cela J, Munne-Bosch S (2014). Plastochromanol-8: Fifty years of research. Phytochemistry.

[CR135] Kruk J, Szymańska R, Nowicka B, Dłużewska J (2016). Function of isoprenoid quinones and chromanols during oxidative stress in plants. N Biotechnol.

[CR136] Kumar M, Kumari P, Gupta V (2010). Biochemical responses of red alga *Gracilaria corticata* (Gracilariales, Rhodophyta) to salinity induced oxidative stress. J Exp Mar Bio Ecol.

[CR137] Kumar M, Kumari P, Gupta V (2010). Differential responses to cadmium induced oxidative stress in marine macroalga *Ulva lactuca* (Ulvales, Chlorophyta). Biometals.

[CR138] Kuo EYH, Cai MS, Lee TM (2020). Ascorbate peroxidase 4 plays a role in the tolerance of Chlamydomonas reinhardtii to photo-oxidative stress. Sci Rep.

[CR139] Küpper H, Sigel A, Sigel H, Sigel RKO (2017). Lead toxicity in plants. Metal ions in life sciences.

[CR140] Küpper H, Andresen E (2016). Mechanisms of metal toxicity in plants. Metallomics.

[CR141] Kusmic C, Barsacchi R, Barsanti L (1998). *Euglena gracilis* as source of the antioxidant vitamin E. Effects of culture conditions in the wild strain and in the natural mutant WZSL. J Appl Phycol.

[CR142] Lane TW, Morel FMM (2000). A biological function for cadmium in marine diatoms. Proc Natl Acad Sci U S A.

[CR143] Larsson MA, Baken S, Gustafsson JP (2013). Vanadium bioavailability and toxicity to soil microorganisms and plants. Environ Toxicol Chem.

[CR144] Latowski D, Szymańska R, Strzałka K (2014) Carotenoids involved in antioxidant system of chloroplasts. In: Ahmad P (ed) Oxidative damage to plants: antioxidant networks and signaling. Academic P, pp 289–319

[CR145] Lee MY, Shin HW (2003). Cadmium-induced changes in antioxidant enzymes from the marine alga *Nannochloropsis oculata*. J Appl Phycol.

[CR146] Lemaire S, Keryer E, Stein M (1999). Heavy-metal regulation of thioredoxin gene expression in *Chlamydomonas reinhardtii*. Plant Physiol.

[CR147] Lemaire SD (2004). The glutaredoxin family in oxygenic photosynthetic organisms. Photosynth Res.

[CR148] León-Vaz A, León R, Giráldez I (2021). Impact of heavy metals in the microalga *Chlorella sorokiniana* and assessment of its potential use in cadmium bioremediation. Aquat Toxicol.

[CR149] Li H, Yao J, Duran R (2021). Toxic response of the freshwater green algae *Chlorella pyrenoidosa* to combined effect of flotation reagent butyl xanthate and nickel. Environ Pollut.

[CR150] Li HF, Gray C, Mico C (2009). Phytotoxicity and bioavailability of cobalt to plants in a range of soils. Chemosphere.

[CR151] Li J, Jia Y, Dong R (2019). Advances in the mechanisms of plant tolerance to manganese toxicity. Int J Mol Sci.

[CR152] Li M, Hu C, Zhu Q (2006). Copper and zinc induction of lipid peroxidation and effects on antioxidant enzyme activities in the microalga *Pavlova viridis* (Prymnesiophyceae). Chemosphere.

[CR153] Li M, Zhu Q, Hu C, Wei (2007). Cobalt and manganese stress in the microalga *Pavlova viridis* (Prymnesiophyceae): Effects on lipid peroxidation and antioxidant enzymes. J Environ Sci.

[CR154] Li N, Qin L, Jin M, et al (2021b) Extracellular adsorption, intracellular accumulation and tolerance mechanisms of *Cyclotella* sp. to Cr(VI) stress. Chemosphere 270 10.1016/j.chemosphere.2020.128662 10.1016/j.chemosphere.2020.128662 33127109

[CR155] Liang X, Zhang L, Natarajan SK, Becker DF (2013). Proline mechanisms of stress survival. Antioxidants Redox Signal.

[CR156] Lin ST, Chiou CW, Chu YL (2016). Enhanced ascorbate regeneration via dehydroascorbate reductase confers tolerance to photo-oxidative stress in *Chlamydomonas reinhardtii*. Plant Cell Physiol.

[CR157] Lin TH, Rao MY, Lu HW (2018). A role for glutathione reductase and glutathione in the tolerance of *Chlamydomonas reinhardtii* to photo-oxidative stress. Physiol Plant.

[CR158] Liu C, Wang X (2020). Superoxide dismutase and ascorbate peroxidase genes in Antarctic endemic brown alga *Ascoseira mirabilis* (Ascoseirales, Phaeophyceae): data mining of a de novo transcriptome. Bot Mar.

[CR159] Liu J, Reid RJ, Smith FA (2000). The mechanism of cobalt toxicity in mung beans. Physiol Plant.

[CR160] Lu L, Wu Y, Ding H, Zhang W (2015). The combined and second exposure effect of copper (II) and chlortetracycline on fresh water algae, *Chlorella pyrenoidosa* and *Microcystis aeruginosa*. Environ Toxicol Pharmacol.

[CR161] Lu MM, Gao F, Li C, Yang HL (2021). Response of microalgae *Chlorella vulgaris* to Cr stress and continuous Cr removal in a membrane photobioreactor. Chemosphere.

[CR162] Luis P, Behnke K, Toepel J, Wilhelm C (2006). Parallel analysis of transcript levels and physiological key parameters allows the identification of stress phase gene markers in *Chlamydomonas reinhardtii* under copper excess. Plant, Cell Environ.

[CR163] Ma X, Chen Y, Liu F (2021). Enhanced tolerance and resistance characteristics of *Scenedesmus obliquus* FACHB-12 with K3 carrier in cadmium polluted water. Algal Res.

[CR164] Machado MD, Soares EV (2016). Short- and long-term exposure to heavy metals induced oxidative stress response in *Pseudokirchneriella subcapitata*. Clean - Soil Air Water.

[CR165] Macomber L, Hausinger RP (2011). Mechanisms of nickel toxicity in microorganisms. Metallomics.

[CR166] Madejón P, Alloway BJ (2013). Vanadium. Heavy metals in soils: trace metals and metalloids in soils and their bioavailability.

[CR167] Maharana D, Jena K, Pise NM, Jagtap TG (2010). Assessment of oxidative stress indices in a marine macro brown alga *Padina tetrastromatica* (Hauck) from comparable polluted coastal regions of the Arabian Sea, west coast of India. J Environ Sci.

[CR168] Mahbub KR, Krishnan K, Naidu R (2017). Mercury toxicity to terrestrial biota. Ecol Indic.

[CR169] Mallick N (2004). Copper-induced oxidative stress in the chlorophycean microalga *Chlorella vulgaris*: response of the antioxidant system. J Plant Physiol.

[CR170] Mallick N, Mohn FH (2000). Reactive oxygen species: response of algal cells. J Plant Physiol.

[CR171] Manimaran K, Karthikeyan P, Ashokkumar S (2012). Effect of copper on growth and enzyme activities of marine diatom, *Odontella mobiliensis*. Bull Environ Contam Toxicol.

[CR172] Marambio-Jones C, Hoek EMV (2010). A review of the antibacterial effects of silver nanomaterials and potential implications for human health and the environment. J Nanoparticle Res.

[CR173] Martins N, Barros L, Ferreira ICFR (2016). In vivo antioxidant activity of phenolic compounds: Facts and gaps. Trends Food Sci Technol.

[CR174] Maruta T, Sawa Y, Shigeoka S, Ishikawa T (2016). Diversity and evolution of ascorbate peroxidase functions in chloroplasts: More than just a classical antioxidant enzyme?. Plant Cell Physiol.

[CR175] Mc Gee D, Gillespie E, Ramawat KG (2019). The bioactivity and chemotaxonomy of microalgal carotenoids. Biodiversity and chemotaxonomy.

[CR176] McGrath SP, Micó C, Curdy R, Zhao FJ (2010). Predicting molybdenum toxicity to higher plants: Influence of soil properties. Environ Pollut.

[CR177] Meena M, Divyanshu K, Kumar S (2019). Regulation of L-proline biosynthesis, signal transduction, transport, accumulation and its vital role in plants during variable environmental conditions. Heliyon.

[CR178] Mehta SK, Gaur JP (1999). Heavy metal-induced proline accumulation and its role in ameliorating metal toxicity in *Chlorella vulgaris*. New Phytol.

[CR179] Mellado M, Contreras RA, González A (2012). Copper-induced synthesis of ascorbate, glutathione and phytochelatins in the marine alga *Ulva compressa* (Chlorophyta). Plant Physiol Biochem.

[CR180] Mène-Saffrané L, DellaPenna D (2010). Biosynthesis, regulation and functions of tocochromanols in plants. Plant Physiol Biochem.

[CR181] Mhamdi A, Queval G, Chaouch S (2010). Catalase function in plants: A focus on *Arabidopsis* mutants as stress-mimic models. J Exp Bot.

[CR182] Michelet L, Roach T, Fischer BB (2013). Down-regulation of catalase activity allows transient accumulation of a hydrogen peroxide signal in *Chlamydomonas reinhardtii*. Plant, Cell Environ.

[CR183] Millaleo R, Reyes-Díaz M, Ivanov AG (2010). Manganese as essential and toxic element for plants: transport, accumulation and resistance mechanisms. J Soil Sci Plant Nutr.

[CR184] Mittler R, Poulos TL (2005) Ascorbate peroxidase. In: Smirnoff N (ed) Antioxidants and reactive oxygen species in plants. Blackwell Publishing Ltd, pp 87–100

[CR185] Mittler R, Vanderauwera S, Gollery M, Van Breusegem F (2004). Reactive oxygen gene network of plants. Trends Plant Sci.

[CR186] Moenne A, González A, Sáez CA (2016). Mechanisms of metal tolerance in marine macroalgae, with emphasis on copper tolerance in Chlorophyta and Rhodophyta. Aquat Toxicol.

[CR187] Mondal S, Kumar V, Singh SP (2020). Phylogenetic distribution and structural analyses of cyanobacterial glutaredoxins (Grxs). Comput Biol Chem.

[CR188] Morelli E, Scarano G (2004). Copper-induced changes of non-protein thiols and antioxidant enzymes in the marine microalga *Phaeodactylum tricornutum*. Plant Sci.

[CR189] Moreno-Garrido I, Pérez S, Blasco J (2015). Toxicity of silver and gold nanoparticles on marine microalgae. Mar Environ Res.

[CR190] Mourato M, Reis R, Martins LL (2012) Characterization of plant antioxidative system in response to abiotic stresses: a focus on heavy metal toxicity. In: Montanaro G, Dichio B (eds) Advances in selected plant physiology aspects. Intech Open, pp 23–44

[CR191] Moussa ID-B, Athmouni K, Chtourou H (2018). Phycoremediation potential, physiological, and biochemical response of *Amphora subtropica* and *Dunaliella* sp. to nickel pollution. J Appl Phycol.

[CR192] Munné-Bosch S (2005). The role of α-tocopherol in plant stress tolerance. J Plant Physiol.

[CR193] Nagajyoti PC, Lee KD, Sreekanth TVM (2010). Heavy metals, occurrence and toxicity for plants: a review. Environ Chem Lett.

[CR194] Nagalakshmi N, Prasad MNV (2001). Responses of glutathione cycle enzymes and glutathione metabolism to copper stress in *Scenedesmus bijugatus*. Plant Sci.

[CR195] Nagalakshmi N, Prasad MNV (1998). Copper-induced oxidative stress in *Scenedesmus bijugatus*: protective role of free radical scavengers. Bull Environ Contam Toxicol.

[CR196] Nanda M, Jaiswal KK, Kumar V (2021). Micro-pollutant Pb(II) mitigation and lipid induction in oleaginous microalgae *Chlorella sorokiniana* UUIND6. Environ Technol Innov.

[CR197] Navrot N, Rouhier N, Gelhaye E, Jacquot JP (2007) Reactive oxygen species generation and antioxidant systems in plant mitochondria. In: Physiologia Plantarum. pp 185–195

[CR198] Nazar R, Iqbal N, Masood A (2012). Cadmium toxicity in plants and role of mineral nutrients in its alleviation. Am J Plant Sci.

[CR199] Neff JM (1997). Ecotoxicology of arsenic in the marine environment. Environ Toxicol Chem.

[CR200] Nguyen-Deroche TLN, Caruso A, Le TT (2012). Zinc affects differently growth, photosynthesis, antioxidant enzyme activities and phytochelatin synthase expression of four marine diatoms. Sci World J.

[CR201] Nielsen HD, Nielsen SL (2010). Adaptation to high light irradiances enhances the photosynthetic Cu^2+^ resistance in Cu^2+^ tolerant and non-tolerant populations of the brown macroalgae *Fucus serratus*. Mar Pollut Bull.

[CR202] Nies DH (1999). Microbial heavy-metal resistance. Appl Microbiol Biotechnol.

[CR203] Niki E (2009). Lipid peroxidation: physiological levels and dual biological effects. Free Radic Biol Med.

[CR204] Nikookar K, Moradshahi A, Hosseini L (2005). Physiological responses of *Dunaliella salina* and *Dunaliella tertiolecta* to copper toxicity. Biomol Eng.

[CR205] Noctor G (2006). Metabolic signalling in defence and stress: the central roles of soluble redox couples. Plant, Cell Environ.

[CR206] Nowicka B, Fesenko T, Walczak J, Kruk J (2020). The inhibitor-evoked shortage of tocopherol and plastoquinol is compensated by other antioxidant mechanisms in *Chlamydomonas reinhardtii* exposed to toxic concentrations of cadmium and chromium ions. Ecotoxicol Environ Saf.

[CR207] Nowicka B, Gruszka J, Kruk J (2013). Function of plastochromanol and other biological prenyllipids in the inhibition of lipid peroxidation - a comparative study in model systems. Biochim Biophys Acta - Biomembr.

[CR208] Nowicka B, Kruk J (2016). Powered by light: Phototrophy and photosynthesis in prokaryotes and its evolution. Microbiol Res.

[CR209] Nowicka B, Kruk J (2013). Reaktywne formy tlenu w roślinach - więcej niż trucizna [Reactive oxygen species - far more than just a poison]. Kosmos.

[CR210] Nowicka B, Kruk J (2010). Occurrence, biosynthesis and function of isoprenoid quinones. Biochim Biophys Acta - Bioenerg.

[CR211] Nowicka B, Pluciński B, Kuczyńska P, Kruk J (2016). Physiological characterization of *Chlamydomonas reinhardtii* acclimated to chronic stress induced by Ag, Cd, Cr, Cu and Hg ions. Ecotoxicol Environ Saf.

[CR212] Nowicka B, Pluciński B, Kuczyńska P, Kruk J (2016). Prenyllipid antioxidants participate in response to acute stress induced by heavy metals in green microalga *Chlamydomonas reinhardtii*. Environ Exp Bot.

[CR213] Nowicka B, Zyzik M, Kapsiak M (2021). Oxidative stress limits growth of *Chlamydomonas reinhardtii* (Chlorophyta, Chlamydomonadales) exposed to copper ions at the early stage of culture growth. Phycologia.

[CR214] Okamoto OK, Asano CS, Aidar E, Colepicolo P (1996). Effects of cadmium on growth and superoxide dismutase activity of the marine microalga *Tetraselmis gracilis* (Prasinophyceae). J Phycol.

[CR215] Okamoto OK, Colepicolo P (1998). Response of superoxide dismutase to pollutant metal stress in the marine dinoflagellate *Gonyaulax polyedra*. Comp Biochem Physiol - C Pharmacol Toxicol Endocrinol.

[CR216] Okamoto OK, Pinto E, Latorre LR (2001). Antioxidant modulation in response to metal-induced oxidative stress in algal chloroplasts. Arch Environ Contam Toxicol.

[CR217] Okamoto OK, Robertson DL, Fagan TF (2001). Different regulatory mechanisms modulate the expression of a dinoflagellate iron-superoxide dismutase. J Biol Chem.

[CR218] Palit S, Sharma A, Talukder G (1994). Effects of cobalt on plants. Bot Rev.

[CR219] Papini A, Gonnelli C, Tani C (2018). Autophagy induced by heavy metal and starvation stress in microalgae. Phytomorphology.

[CR220] Parmar P, Kumari N, Sharma V (2013). Structural and functional alterations in photosynthetic apparatus of plants under cadmium stress. Bot Stud.

[CR221] Paschke MW, Valdecantos A, Redente EF (2005). Manganese toxicity thresholds for restoration grass species. Environ Pollut.

[CR222] Passaia G, Margis-Pinheiro M (2015). Glutathione peroxidases as redox sensor proteins in plant cells. Plant Sci.

[CR223] Passardi F, Bakalovic N, Teixeira FK (2007). Prokaryotic origins of the non-animal peroxidase superfamily and organelle-mediated transmission to eukaryotes. Genomics.

[CR224] Patra M, Sharma A (2000). Mercury toxicity in plants. Bot Rev.

[CR225] Penen F, Isaure MP, Dobritzsch D (2017). Pools of cadmium in *Chlamydomonas reinhardtii* revealed by chemical imaging and XAS spectroscopy. Metallomics.

[CR226] Perales-Vela HV, Peña-Castro JM, Cañizares-Villanueva RO (2006). Heavy metal detoxification in eukaryotic microalgae. Chemosphere.

[CR227] Peterson RL, Galaleldeen A, Villarreal J (2016). The phylogeny and active site design of eukaryotic copper-only superoxide dismutases. J Biol Chem.

[CR228] Pikula KS, Zakharenko AM, Aruoja V (2019). Oxidative stress and its biomarkers in microalgal ecotoxicology. Curr Opin Toxicol.

[CR229] Pillai S, Behra R, Nestler H (2014). Linking toxicity and adaptive responses across the transcriptome, proteome, and phenotype of *Chlamydomonas reinhardtii* exposed to silver. Proc Natl Acad Sci U S A.

[CR230] Pinto E, Sigaud-Kutner TCS, Leitão MAS (2003). Heavy metal-induced oxidative stress in algae. J Phycol.

[CR231] Piotrowska-Niczyporuk A, Bajguz A, Talarek M (2015). The effect of lead on the growth, content of primary metabolites, and antioxidant response of green alga *Acutodesmus obliquus* (Chlorophyceae). Environ Sci Pollut Res.

[CR232] Piotrowska-Niczyporuk A, Bajguz A, Zambrzycka E, Godlewska-Żyłkiewicz B (2012). Phytohormones as regulators of heavy metal biosorption and toxicity in green alga *Chlorella vulgaris* (Chlorophyceae). Plant Physiol Biochem.

[CR233] Pourrut B, Shahid M, Dumat C, Whitacre DM (2011). Lead uptake, toxicity, and detoxification in plants. Reviews of environmental contamination and toxicology.

[CR234] Prasad MNV (1995). Cadmium toxicity and tolerance in vascular plants. Environ Exp Bot.

[CR235] Purcell TW, Peters JJ (1998). Sources of silver in the environment. Environ Toxicol Chem.

[CR236] Qian H, Li J, Pan X (2011). Combined effect of copper and cadmium on heavy metal ion bioaccumulation and antioxidant enzymes induction in *Chlorella vulgaris*. Bull Environ Contam Toxicol.

[CR237] Rai UN, Singh NK, Upadhyay AK, Verma S (2013). Chromate tolerance and accumulation in *Chlorella vulgaris* L.: role of antioxidant enzymes and biochemical changes in detoxification of metals. Bioresour Technol.

[CR238] Rao ASVC, Reddy AR, Khan NA, Singh S, Umar S (2008). Glutathione reductase: a putative redox regulatory system in plant cells. Sulfur assimilation and abiotic stress in plants.

[CR239] Raskin I, Kumar PN, Dushenkov S, Salt DE (1994). Bioconcentration of heavy metals by plants. Curr Opin Biotechnol.

[CR240] Ratte HT (1999). Bioaccumulation and toxicity of silver compounds: a review. Environ Toxicol Chem.

[CR241] Rehder D (2015). The role of vanadium in biology. Metallomics.

[CR242] Rejeb KB, Abdelly C, Savouré A (2014). How reactive oxygen species and proline face stress together. Plant Physiol Biochem.

[CR243] Rezayian M, Niknam V, Ebrahimzadeh H (2019). Oxidative damage and antioxidative system in algae. Toxicol Reports.

[CR244] Rice-Evans CA, Miller NJ, Paganga G (1997). Antioxidant properties of phenolic compounds. Trends Plant Sci.

[CR245] Rijstenbil JW, Derksen JWM, Gerringa LJA (1994). Oxidative stress induced by copper: defense and damage in the marine planktonic diatom *Ditylum brightwellii*, grown in continuous cultures with high and low zinc levels. Mar Biol.

[CR246] Rijstenbil JW, Sandee A, Van Drie J, Wijnholds JA (1994). Interaction of toxic trace metals and mechanisms of detoxification in the planktonic diatoms *Ditylum brightwellii* and *Thalassiosira pseudonana*. FEMS Microbiol Rev.

[CR247] Ritter A, Dittami SM, Goulitquer S (2014). Transcriptomic and metabolomic analysis of copper stress acclimation in *Ectocarpus siliculosus* highlights signaling and tolerance mechanisms in brown algae. BMC Plant Biol.

[CR248] Rouhier N, Jacquot JP (2002). Plant peroxiredoxins: alternative hydroperoxide scavenging enzymes. Photosynth Res.

[CR249] Rouhier N, Lemaire SD, Jacquot JP (2008). The role of glutathione in photosynthetic organisms: emerging functions for glutaredoxins and glutathionylation. Annu Rev Plant Biol.

[CR250] Rowley DA, Halliwell B (1983). Superoxide-dependent and ascorbate-dependent formation of hydroxyl radicals in the presence of copper salts: a physiologically significant reaction?. Arch Biochem Biophys.

[CR251] Sabatini SE, Juárez ÁB, Eppis MR (2009). Oxidative stress and antioxidant defenses in two green microalgae exposed to copper. Ecotoxicol Environ Saf.

[CR252] Sáez CA, Roncarati F, Moenne A (2015). Copper-induced intra-specific oxidative damage and antioxidant responses in strains of the brown alga *Ectocarpus siliculosus* with different pollution histories. Aquat Toxicol.

[CR253] Safafar H, Van WJ, Møller P, Jacobsen C (2015). Carotenoids, phenolic compounds and tocopherols contribute to the antioxidative properties of some microalgae species grown on industrial wastewater. Mar Drugs.

[CR254] Sánchez-Machado DI, López-Hernández J, Paseiro-Losada P (2002). High-performance liquid chromatographic determination of α-tocopherol in macroalgae. J Chromatogr A.

[CR255] Sarkar A, Ravindran G, Krishnamurthy V (2013). A brief review on the effect of cadmium toxicity: from cellular to organ level. Int J Bio-Technology Res.

[CR256] Sathasivam R, Ki JS (2019). Heat shock protein genes in the green alga *Tetraselmis suecica* and their role against redox and non-redox active metals. Eur J Protistol.

[CR257] Sauser KR, Liu JK, Wong TY (1997). Identification of a copper-sensitive ascorbate peroxidase in the unicellular green alga *Selenastrum capricornutum*. Biometals.

[CR258] Schmidt A, Gube M, Schmidt A, Kothe E (2009). In silico analysis of nickel containing superoxide dismutase evolution and regulation. J Basic Microbiol.

[CR259] Schoepp-Cothenet B, Van Lis R, Atteia A (2013). On the universal core of bioenergetics. Biochim Biophys Acta - Bioenerg.

[CR260] Schützendübel A, Polle A (2002). Plant responses to abiotic stresses: heavy metal-induced oxidative stress and protection by mycorrhization. J Exp Bot.

[CR261] Seregin IV, Kozhevnikova AD (2006). Physiological role of nickel and its toxic effects on higher plants. Russ J Plant Physiol.

[CR262] Serrano A, Llobell A (1993). Occurrence of two isoforms of glutathione reductase in the green alga *Chlamydomonas reinhardtii*. Planta.

[CR263] Shahzad B, Tanveer M, Rehman A (2018). Nickel; whether toxic or essential for plants and environment - a review. Plant Physiol Biochem.

[CR264] Shanker AK, Cervantes C, Loza-Tavera H, Avudainayagam S (2005). Chromium toxicity in plants. Environ Int.

[CR265] Sharma P, Dubey RS (2005). Lead toxicity in plants. Brazilian J Plant Physiol.

[CR266] Sharma SS, Dietz KJ (2009). The relationship between metal toxicity and cellular redox imbalance. Trends Plant Sci.

[CR267] Shigeoka S, Onishi T, Nakano Y, Kitaoka S (1987). Characterization and physiological function of glutathione reductase in *Euglena gracilis* z. Biochem J.

[CR268] Shigeoka S, Yasumoto R, Onishi T (1987). Properties of monodehydroascorbate reductase and dehydroascorbate reductase and their participation in the regeneration of ascorbate in *Euglena gracilis*. Microbiology.

[CR269] Shim J-B, Klochkova TA, Han J-W (2011). Comparative proteomics of the mixotrophic dinoflagellate *Prorocentrum micans* growing in different trophic modes. Algae.

[CR270] Shrivastava S, Shrivastav A, Sharma J (2015). Detoxification mechanisms of mercury toxicity in plants: A review. Recent Adv Biol Med.

[CR271] Singh AL, Jat RS, Chaudhari V (2010). Toxicities and tolerance of mineral elements boron, cobalt, molybdenum and nickel in crop plants. Plant Stress.

[CR272] Singh HP, Mahajan P, Kaur S (2013). Chromium toxicity and tolerance in plants. Environ Chem Lett.

[CR273] Singh R, Upadhyay AK, Chandra P, Singh DP (2018). Sodium chloride incites reactive oxygen species in green algae *Chlorococcum humicola* and *Chlorella vulgaris*: implication on lipid synthesis, mineral nutrients and antioxidant system. Bioresour Technol.

[CR274] Sirikhachornkit A, Niyogi KK, Rebeiz CA, Benning C, Bohnert HJ (2010). Antioxidants and photo-oxidative stress responses in plants and algae. The chloroplast.

[CR275] Siripornadulsil S, Traina S, Verma DPS, Sayre RT (2002). Molecular mechanisms of proline-mediated tolerance to toxic heavy metals in transgenic microalgae. Plant Cell.

[CR276] Škodová-Sveráková I, Záhonová K, Bučková B (2020). Catalase and ascorbate peroxidase in euglenozoan protists. Pathogens.

[CR277] Slaveykova VI, Majumdar S, Regier N (2021). Metabolomic responses of green alga *Chlamydomonas reinhardtii* exposed to sublethal concentrations of inorganic and methylmercury. Environ Sci Technol.

[CR278] Smirnoff N (2005) Ascorbate, tocopherol and carotenoids: metabolism, pathway engineering and functions. In: Smirnoff N (ed) Antioxidants and reactive oxygen species in plants. Blackwell Publishing Ltd, pp 53–86

[CR279] Smith KL, Hann AC, Harwood JL (1986). The subcellular localisation of absorbed copper in *Fucus*. Physiol Plant.

[CR280] Soldo D, Hari R, Sigg L, Behra R (2005). Tolerance of *Oocystis nephrocytioides* to copper: Intracellular distribution and extracellular complexation of copper. Aquat Toxicol.

[CR281] Sreekanth TVM, Nagajyothi PC, Lee KD, Prasad TNVKV (2013). Occurrence, physiological responses and toxicity of nickel in plants. Int J Environ Sci Technol.

[CR282] Stahl-Rommel S, Kalra I, D’Silva S (2021). Cyclic electron flow (CEF) and ascorbate pathway activity provide constitutive photoprotection for the photopsychrophile, Chlamydomonas sp. UWO 241 (renamed Chlamydomonas priscuii). Photosynth Res.

[CR283] Stohs SJ, Bagchi D (1995). Oxidative mechanisms in the toxicity of metal ions. Free Radic Biol Med.

[CR284] Stoiber TL, Shafer MM, Armstrong DE (2013). Induction of reactive oxygen species in *Chlamydomonas reinhardtii* in response to contrasting trace metal exposures. Environ Toxicol.

[CR285] Štork F, Bačkor M, Klejdus B (2013). Changes of metal-induced toxicity by H_2_O_2_/NO modulators in *Scenedesmus quadricauda* (Chlorophyceae). Environ Sci Pollut Res.

[CR286] Sunda W, Kieber DJ, Kiene RP, Huntsman S (2002). An antioxidant function for DMSP and DMS in marine algae. Nature.

[CR287] Sytar O, Kumar A, Latowski D (2013). Heavy metal-induced oxidative damage, defense reactions, and detoxification mechanisms in plants. Acta Physiol Plant.

[CR288] Szőllősi R (2014) Superoxide dismutase (SOD) and abiotic stress tolerance in plants: an overview. In: Ahmad P (ed) Oxidative damage to plants: antioxidant networks and signaling. Academic Press, pp 89–129

[CR289] Szymańska R, Nowicka B, Kruk J (2017). Vitamin E - occurrence, biosynthesis by plants and functions in human nutrition. Mini-Reviews Med Chem.

[CR290] Takaichi S (2011). Carotenoids in algae: distributions, biosyntheses and functions. Mar Drugs.

[CR291] Takami R, Almeida JV, Vardaris CV (2012). The interplay between thiol-compounds against chromium (VI) in the freshwater green alga *Monoraphidium convolutum*: Toxicology, photosynthesis, and oxidative stress at a glance. Aquat Toxicol.

[CR292] Takeda T, Yoshimura K, Ishikawa T, Shigeoka S (1998). Purification and characterization of ascorbate peroxidase in *Chlorella vulgaris*. Biochimie.

[CR293] Tamaki S, Mochida K, Suzuki K (2021). Diverse biosynthetic pathways and protective functions against environmental stress of antioxidants in microalgae. Plants.

[CR294] Tran TA, Popova P (2013) Functions and toxicity of cadmium in plants: recent advances and future prospects. Turk J Botany 1–1310.3906/bot-1112-16

[CR295] Triantaphylidès C, Havaux M (2009). Singlet oxygen in plants: production, detoxification and signaling. Trends Plant Sci.

[CR296] Triantaphylidès C, Krischke M, Hoeberichts FA (2008). Singlet oxygen is the major reactive oxygen species involved in photooxidative damage to plants. Plant Physiol.

[CR297] Tripathi BN, Mehta SK, Amar A, Gaur JP (2006). Oxidative stress in *Scenedesmus* sp. during short- and long-term exposure to Cu^2+^ and Zn^2+^. Chemosphere.

[CR298] Tripathi S, Arora N, Pruthi V, Poluri KM (2021). Elucidating the bioremediation mechanism of Scenedesmus sp. IITRIND2 under cadmium stress. Chemosphere.

[CR299] Tripathi S, Poluri KM (2021). Heavy metal detoxification mechanisms by microalgae: Insights from transcriptomics analysis. Environ Pollut.

[CR300] Tsonev T, Cebola Lidon FJ (2012). Zinc in plants - an overview. Emirates J Food Agric.

[CR301] Ugya AY, Imam TS, Li A (2020). Antioxidant response mechanism of freshwater microalgae species to reactive oxygen species production: a mini review. Chem Ecol.

[CR302] Van Breusegem F, Bailey-Serres J, Mittler R (2008). Unraveling the tapestry of networks involving reactive oxygen species in plants. Plant Physiol.

[CR303] Van Breusegem F, Vranová E, Dat JF, Inzé D (2001). The role of active oxygen species in plant signal transduction. Plant Sci.

[CR304] Venkatesh J, Park SW (2014). Role of L-ascorbate in alleviating abiotic stresses in crop plants. Bot Stud.

[CR305] Verbruggen N, Hermans C (2008). Proline accumulation in plants: a review. Amino Acids.

[CR306] Verbruggen N, Hermans C, Schat H (2009). Mechanisms to cope with arsenic or cadmium excess in plants. Curr Opin Plant Biol.

[CR307] Vranová E, Inzé D, Van Breusegem F (2002). Signal transduction during oxidative stress. J Exp Bot.

[CR308] Wakao S, Niyogi KK (2021). Chlamydomonas as a model for reactive oxygen species signaling and thiol redox regulation in the green lineage. Plant Physiol.

[CR309] Wang H, Abassi S, Ki JS (2019). Origin and roles of a novel copper-zinc superoxide dismutase (CuZnSOD) gene from the harmful dinoflagellate *Prorocentrum minimum*. Gene.

[CR310] Wang H, Ebenezer V, Ki JS (2018). Photosynthetic and biochemical responses of the freshwater green algae *Closterium ehrenbergii* Meneghini (Conjugatophyceae) exposed to the metal coppers and its implication for toxicity testing. J Microbiol.

[CR311] Wang H, Ki JS (2020). Molecular identification, differential expression and protective roles of iron/manganese superoxide dismutases in the green algae *Closterium ehrenbergii* against metal stress. Eur J Protistol.

[CR312] Wang H, Ki JS (2020). Molecular characterization and expression analysis of copper-zinc superoxide dismutases from the freshwater alga *Closterium ehrenbergii* under metal stress. Environ Toxicol.

[CR313] Wang H, Kim H, Ki JS (2021). Transcriptome survey, molecular identification, and expression analysis of stress-responsive genes in the toxic dinoflagellate *Alexandrium pacificum* under algicidal agents and metal stresses. J Appl Phycol.

[CR314] Wang L, Kang Y, Liang S (2018). Synergistic effect of co-exposure to cadmium (II) and 4-n-nonylphenol on growth inhibition and oxidative stress of *Chlorella sorokiniana*. Ecotoxicol Environ Saf.

[CR315] Wang L, Yu DD, Xu D, Li YX (2021). Physiological and proteomic alterations in *Macrocystis pyrifera* under chromium(VI) stress. Russ J Mar Biol.

[CR316] Wang Y, Wang S, Xu P (2015). Review of arsenic speciation, toxicity and metabolism in microalgae. Rev Environ Sci Biotechnol.

[CR317] Waśkiewicz A, Beszterda M, Goliński P (2014a) Nonenzymatic antioxidants in plants. In: Ahmad P (ed) Oxidative damage to plants: antioxidant networks and signaling. Academic Press, pp 201–234

[CR318] Waśkiewicz A, Gładysz O, Szentner K, Goliński P (2014b) Role of glutathione in abiotic stress tolerance. In: Ahmad P (ed) Oxidative damage to plants: antioxidant networks and signaling. Academic Press, pp 149–181

[CR319] Wei YY, Zheng Q, Liu ZP, Yang ZM (2011). Regulation of tolerance of *Chlamydomonas reinhardtii* to heavy metal toxicity by heme oxygenase-1 and carbon monoxide. Plant Cell Physiol.

[CR320] Wever R, Kustin K (1990). Vanadium: A biologically relevant element. Adv Inorg Chem.

[CR321] Wheeler G, Ishikawa T, Pornsaksit V, Smirnoff N (2015). Evolution of alternative biosynthetic pathways for vitamin C following plastid acquisition in photosynthetic eukaryotes. Elife.

[CR322] Whittaker JW (2012). Non-heme manganese catalase - the “other” catalase. Arch Biochem Biophys.

[CR323] Wolfe-Simon F, Grzebyk D, Schofield O, Falkowski PG (2005). The role and evolution of superoxide dismutases in algae. J Phycol.

[CR324] Wood JM, Wang H-K (1983). Microbial resistance to heavy metals. Environ Sci Technol.

[CR325] Wu J-T, Chang S-J, Chou T-L (1995). Intracellular proline accumulation in some algae exposed to copper and cadmium. Bot Bull Acad Sin.

[CR326] Wu T-M, Hsu Y-T, Lee T-M (2009). Effects of cadmium on the regulation of antioxidant enzyme activity, gene expression, and antioxidant defenses in the marine macroalga *Ulva fasciata*. Bot Stud.

[CR327] Wu TM, Lee TM (2008). Regulation of activity and gene expression of antioxidant enzymes in *Ulva fasciata* Delile (Ulvales, Chlorophyta) in response to excess copper. Phycologia.

[CR328] Wu Y, Wang WX (2014). Intracellular speciation and transformation of inorganic mercury in marine phytoplankton. Aquat Toxicol.

[CR329] Xing C, Li J, Lam SM (2021). The role of glutathione-mediated triacylglycerol synthesis in the response to ultra-high cadmium stress in *Auxenochlorella protothecoides*. J Environ Sci (china).

[CR330] Yadav SK (2010). Heavy metals toxicity in plants: an overview on the role of glutathione and phytochelatins in heavy metal stress tolerance of plants. South African J Bot.

[CR331] Yan A, Chen Z (2019). Impacts of silver nanoparticles on plants: a focus on the phytotoxicity and underlying mechanism. Int J Mol Sci.

[CR332] Yeh HL, Lin TH, Chen CC (2019). Monodehydroascorbate reductase plays a role in the tolerance of *Chlamydomonas reinhardtii* to photooxidative stress. Plant Cell Physiol.

[CR333] Yruela I (2009). Copper in plants: acquisition, transport and interactions. Funct Plant Biol.

[CR334] Yruela I (2005). Copper in plants. Brazilian J Plant Physiol.

[CR335] Yun CJ, Hwang KO, Han SS, Ri HG (2019). The effect of salinity stress on the biofuel production potential of freshwater microalgae *Chlorella vulgaris* YH703. Biomass Bioenerg.

[CR336] Zaffagnini M, Bedhomme M, Groni H (2012). Glutathionylation in the photosynthetic model organism *Chlamydomonas reinhardtii*: a proteomic survey. Mol Cell Proteomics.

[CR337] Zámocký M, Gasselhuber B, Furtmüller PG, Obinger C (2012). Molecular evolution of hydrogen peroxide degrading enzymes. Arch Biochem Biophys.

[CR338] Zechmann B (2018). Compartment-specific importance of ascorbate during environmental stress in plants. Antioxidants Redox Signal.

[CR339] Zhang CY, Chen GF, Wang YY (2019). Molecular characterization of heat shock protein 90 from the dinoflagellate *Prorocentrum donghaiense* and its transcriptional response to thermal, copper and nutrient stresses. Mar Biol Res.

[CR340] Zhang LP, Mehta SK, Liu ZP, Yang ZM (2008). Copper-induced proline synthesis is associated with nitric oxide generation in *Chlamydomonas reinhardtii*. Plant Cell Physiol.

[CR341] Zhang S, He Y, Sen B, Wang G (2020). Reactive oxygen species and their applications toward enhanced lipid accumulation in oleaginous microorganisms. Bioresour Technol.

[CR342] Zhao Z, Xu L, Wang Y (2021). Toxicity mechanism of silver nanoparticles to *Chlamydomonas reinhardtii*: photosynthesis, oxidative stress, membrane permeability, and ultrastructure analysis. Environ Sci Pollut Res.

[CR343] Zheng Q, Cheng ZZ, Yang ZM (2013). HISN3 mediates adaptive response of *Chlamydomonas reinhardtii* to excess nickel. Plant Cell Physiol.

[CR344] Zheng Q, Meng Q, Wei YY, Yang ZM (2011). Alleviation of copper-induced oxidative damage in *Chlamydomonas reinhardtii* by carbon monoxide. Arch Environ Contam Toxicol.

[CR345] Zhu Q, Zhang M, Bao J, Liu J (2021). Physiological, metabolomic, and transcriptomic analyses reveal the dynamic redox homeostasis upon extended exposure of *Dunaliella salina* GY-H13 cells to Cd. Ecotoxicol Environ Saf.

[CR346] Zolotareva EK, Mokrosnop VM, Stepanov SS (2019). Polyphenol compounds of macroscopic and microscopic algae. Int J Algae.

